# Supplementary material for: Genetic effects on the skin methylome in healthy older twins
Source: Am J Hum Genet. 2024 Aug 12;111(9):1932–52. doi: 10.1016/j.ajhg.2024.07.010 (PMC11393713; doi:10.1016/j.ajhg.2024.07.010)
Supplement: Document S1. Figures S1–S17 [file mmc1.pdf]

The American Journal of Human Genetics, Volume *111*

Supplemental information

Genetic effects on the skin methylome in healthy older twins

Christopher J. Shore, Sergio Villicaña, Julia S. El-Sayed Moustafa, Amy L. Roberts, David A. Gunn, Veronique Bataille, Panos Deloukas, Tim D. Spector, Kerrin S. Small, and Jordana T. Bell

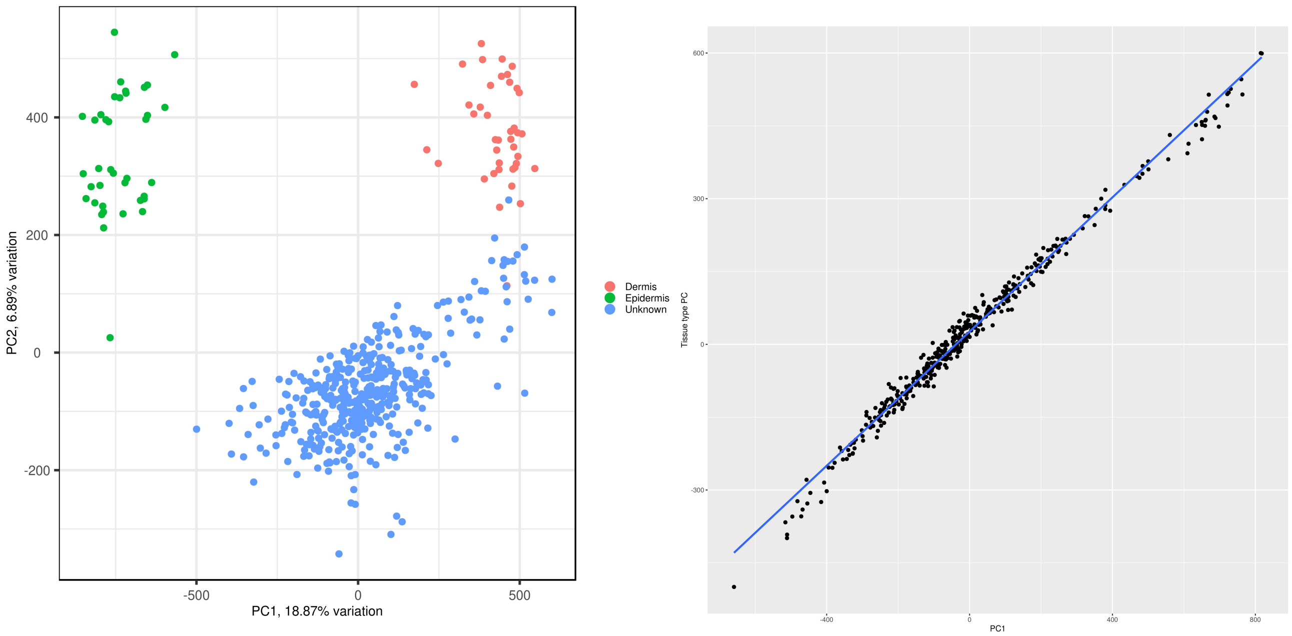

Figure S1. (a) Biplot of PC1 and PC2 from PCA of whole skin DNA methylation data from 414 samples, with dermis and epidermis DNA methylation data from 92 samples from Vandiver et al.[1] (b) Scatter plot of PC1 from S1a ("Tissue Type PC") with PC1 from PCA on just the 414 whole skin DNA methylation data.

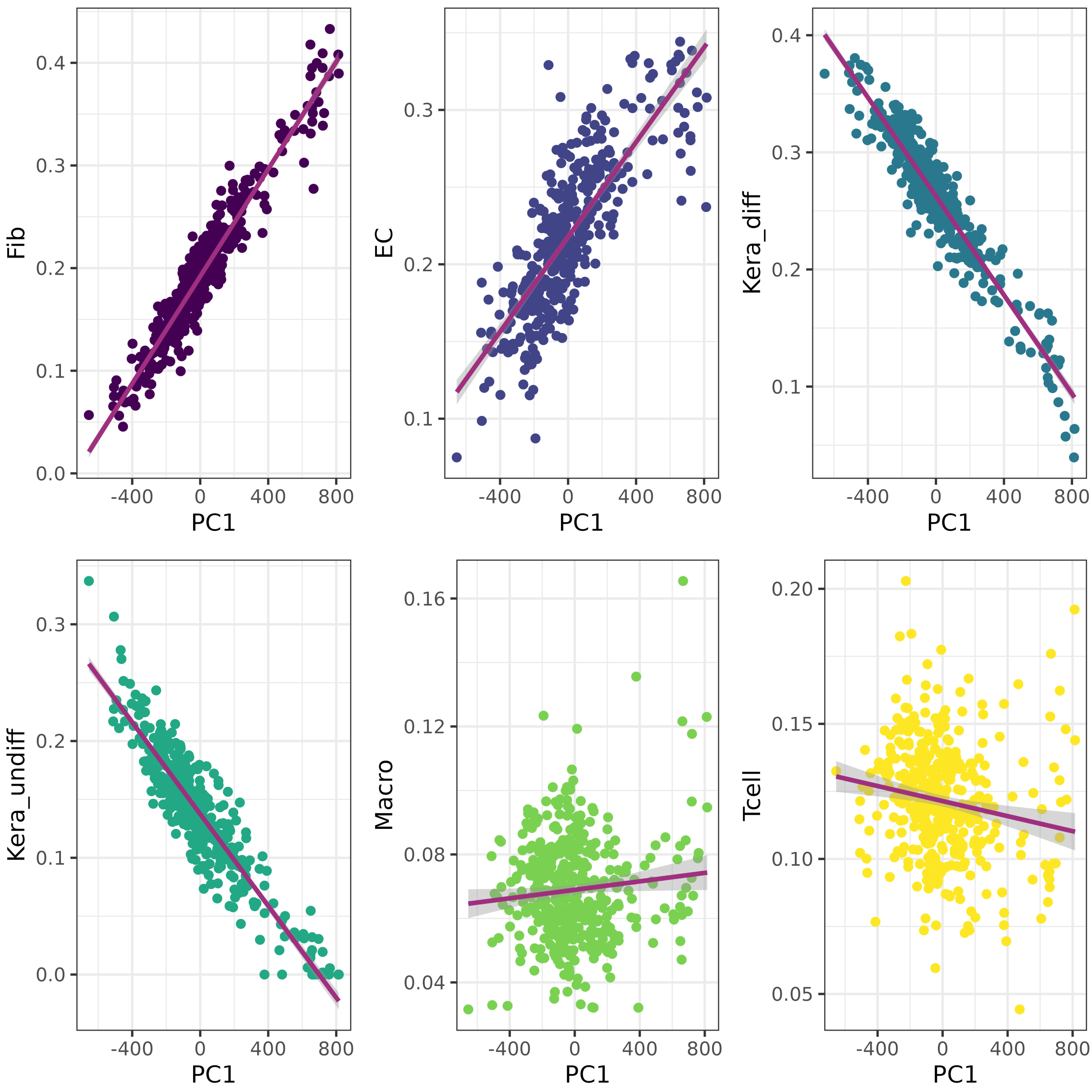

Figure S2. Scatter plots of PC1 from PCA of whole skin DNA methylation data from 414 samples vs EpiSCORE cell-type estimates for each sample.

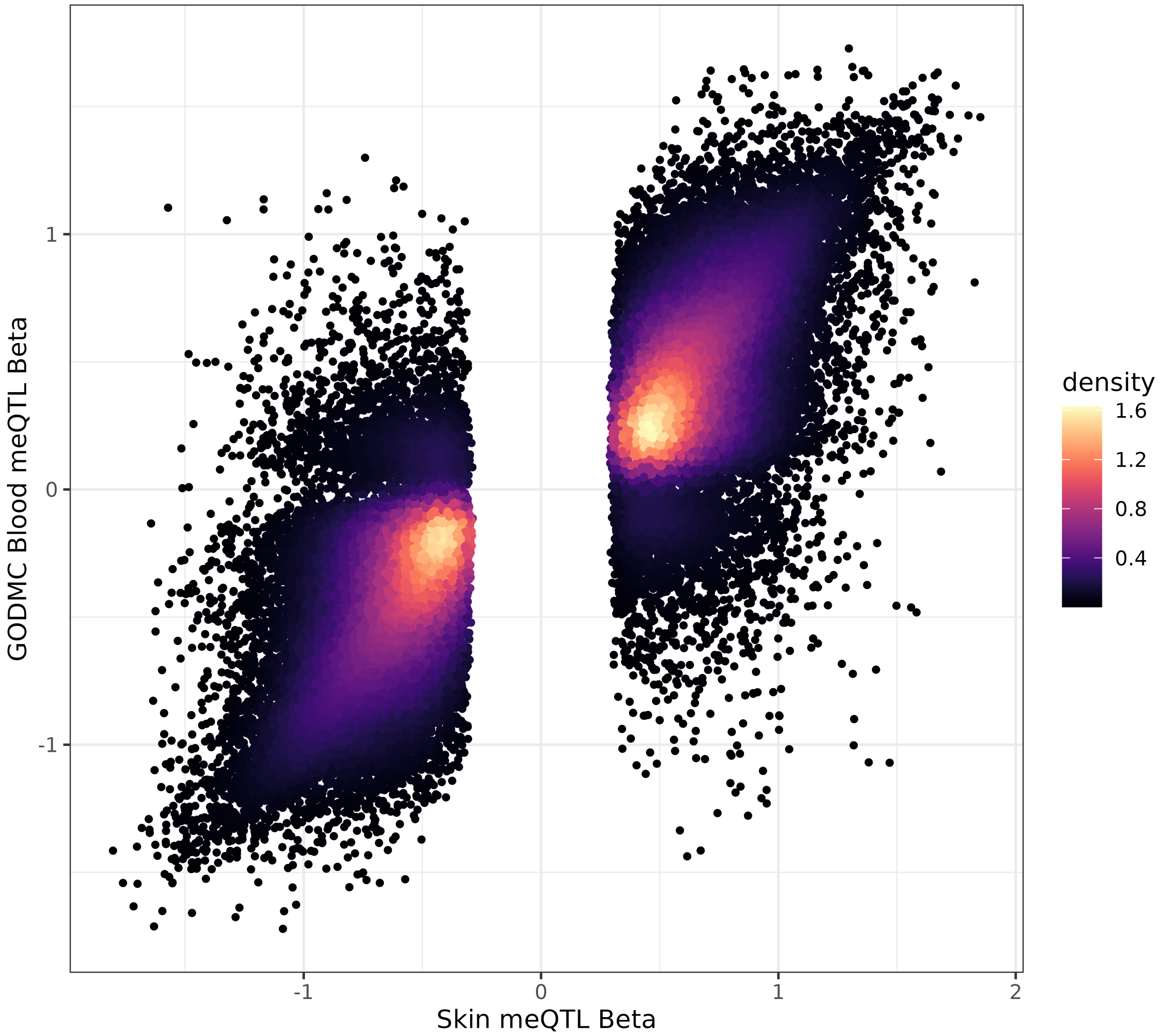

Figure S3. Scatter plot of significant whole skin *cis*-meQTL effect betas against blood *cis*-meQTL effect betas from Min et al.,[2], coloured by the density of points.

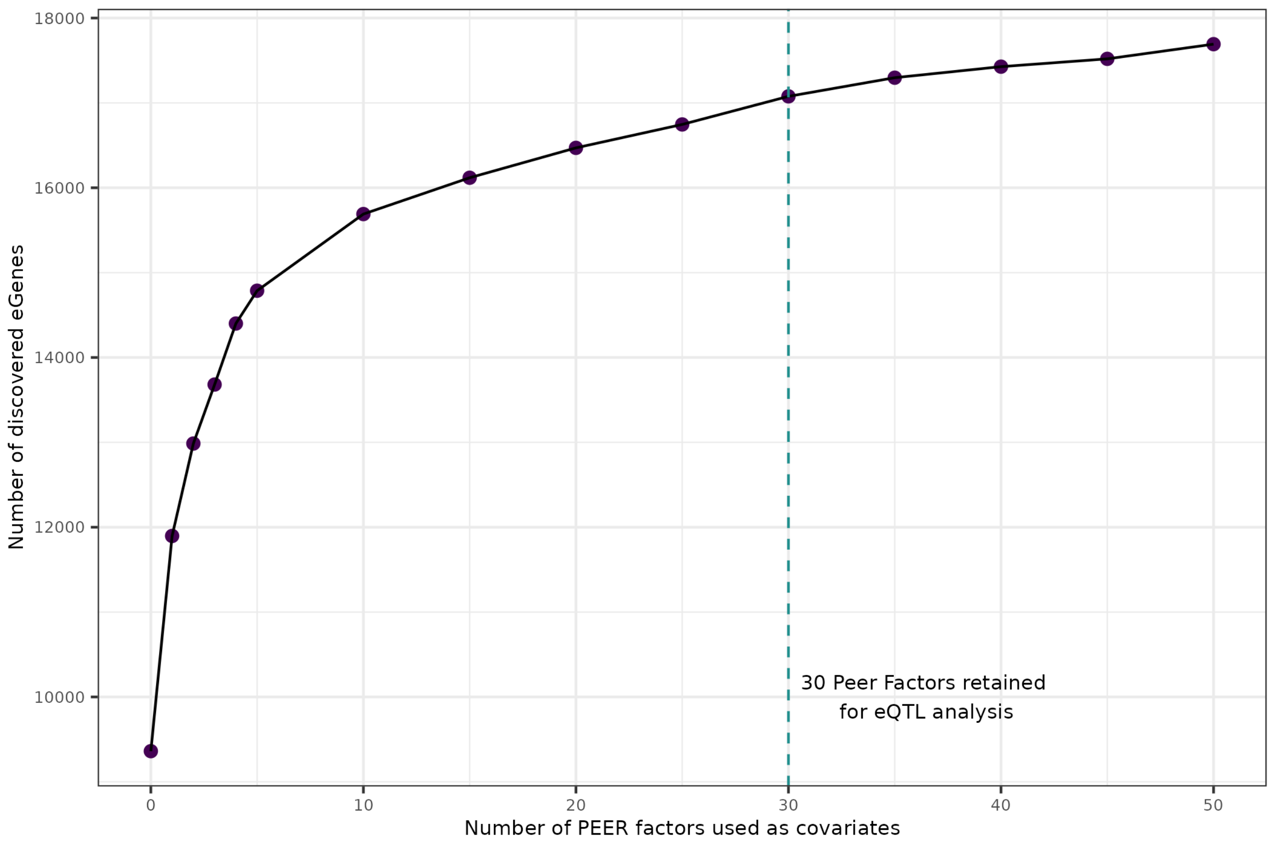

Figure S4. Number of discovered eGenes in whole skin at a Benjamini-Hochberg FDR of 5% in cis-eQTL analyses with different numbers of PEER factors included as covariates. 30 PEER factors were retained for our primary cis-eQTL analysis, to maximise eGene discovery whilst avoiding over-fitting.

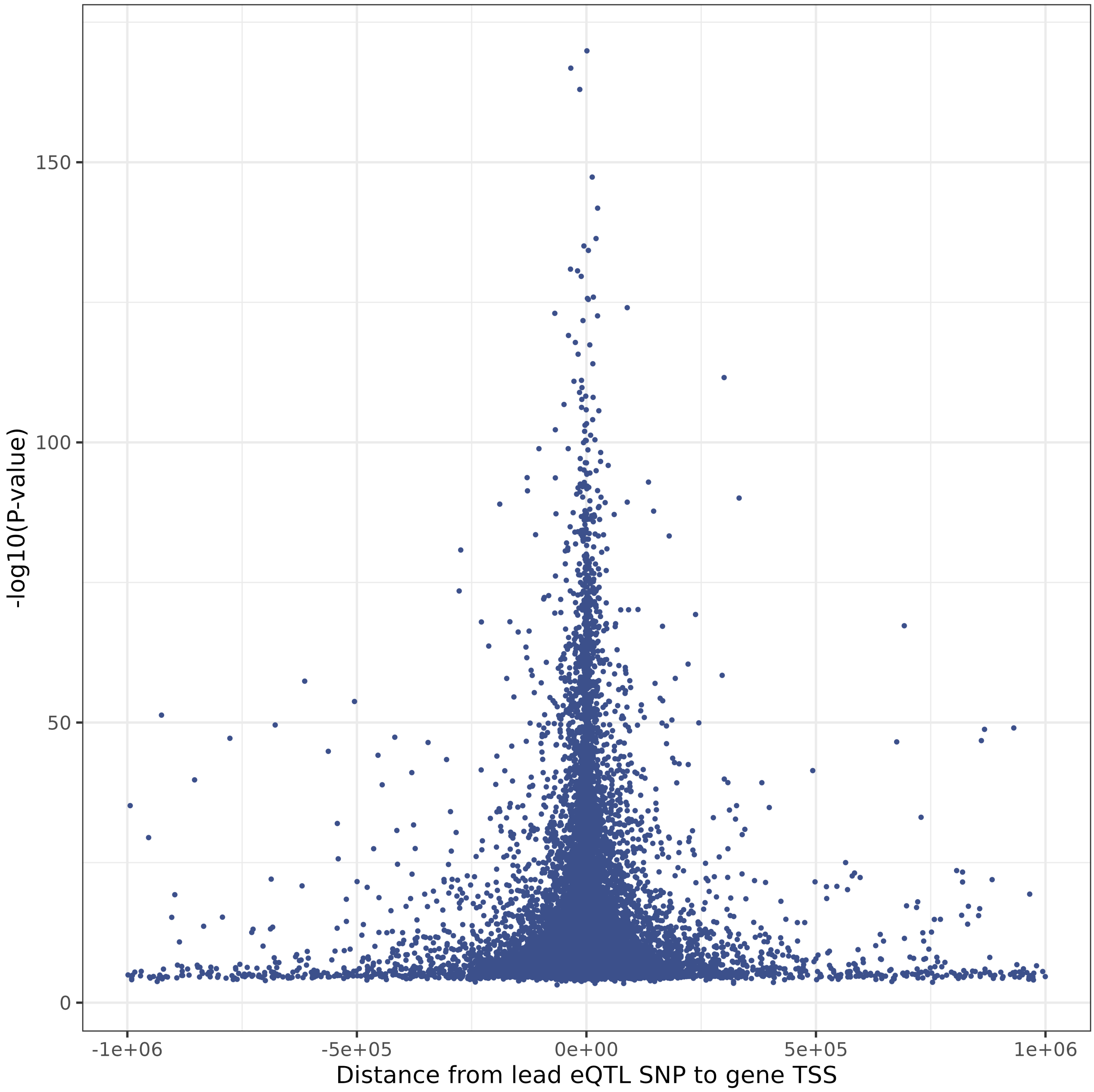

Figure S5. Distance from lead eQTL SNP from conditional analysis to TSS of its eGene vs -log10(P-value) of the eQTL association.

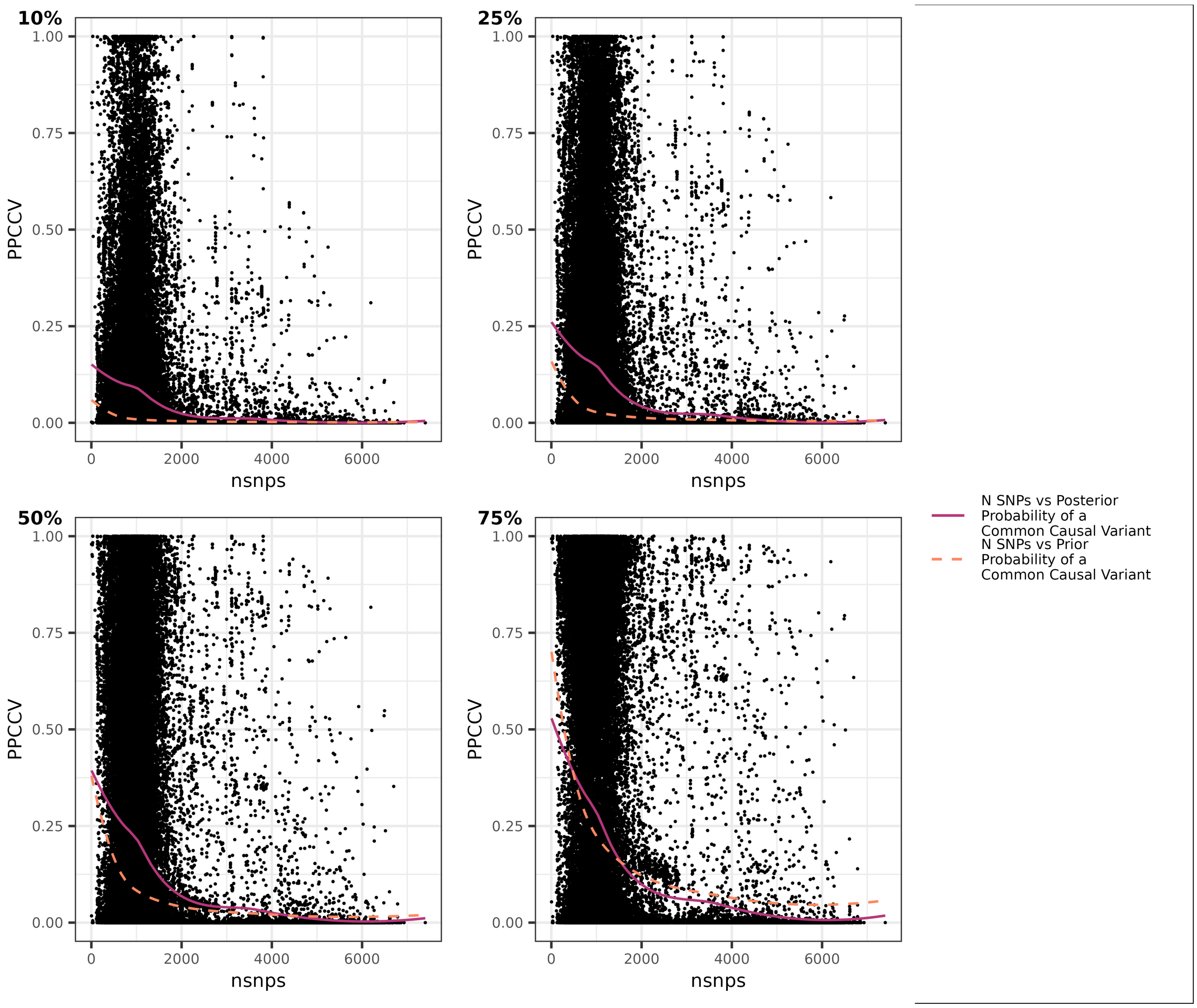

Figure S6. Plots used to find the optimal value of p12 in eQTL-meQTL co-localisation analysis. NSNPs vs observed Posterior Probability of a Common Causal Variant for p12 values corresponding to 10%, 25%, 50%, and 75% probability of a causal cis-eQTL SNP also being a causal cis-meQTL SNP. The purple curve is the loess smoothed curve of these points. The orange dashed line is the loess smoothed curve of NSNPs vs the Prior Probability of a Common Causal Variant (not plotted), calculated using the formula described by Guo *et al.*,[3].

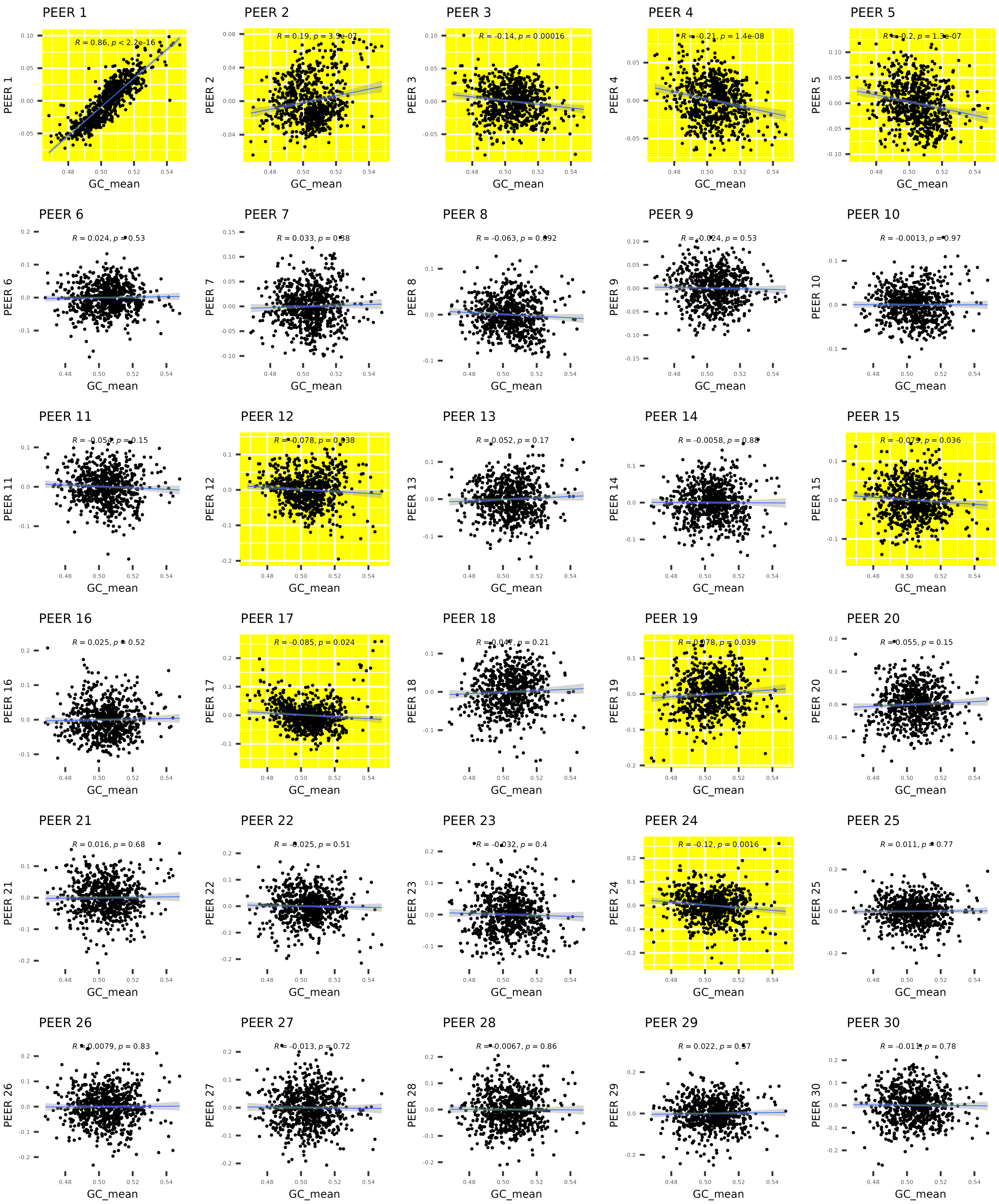

Figure S7. Scatter plots showing the correlation between the 30 PEER factors used as covariates in the skin eQTL analysis and the mean GC content per RNA sample. A yellow background indicates a nominally significant correlation (Pearson P<0.05).

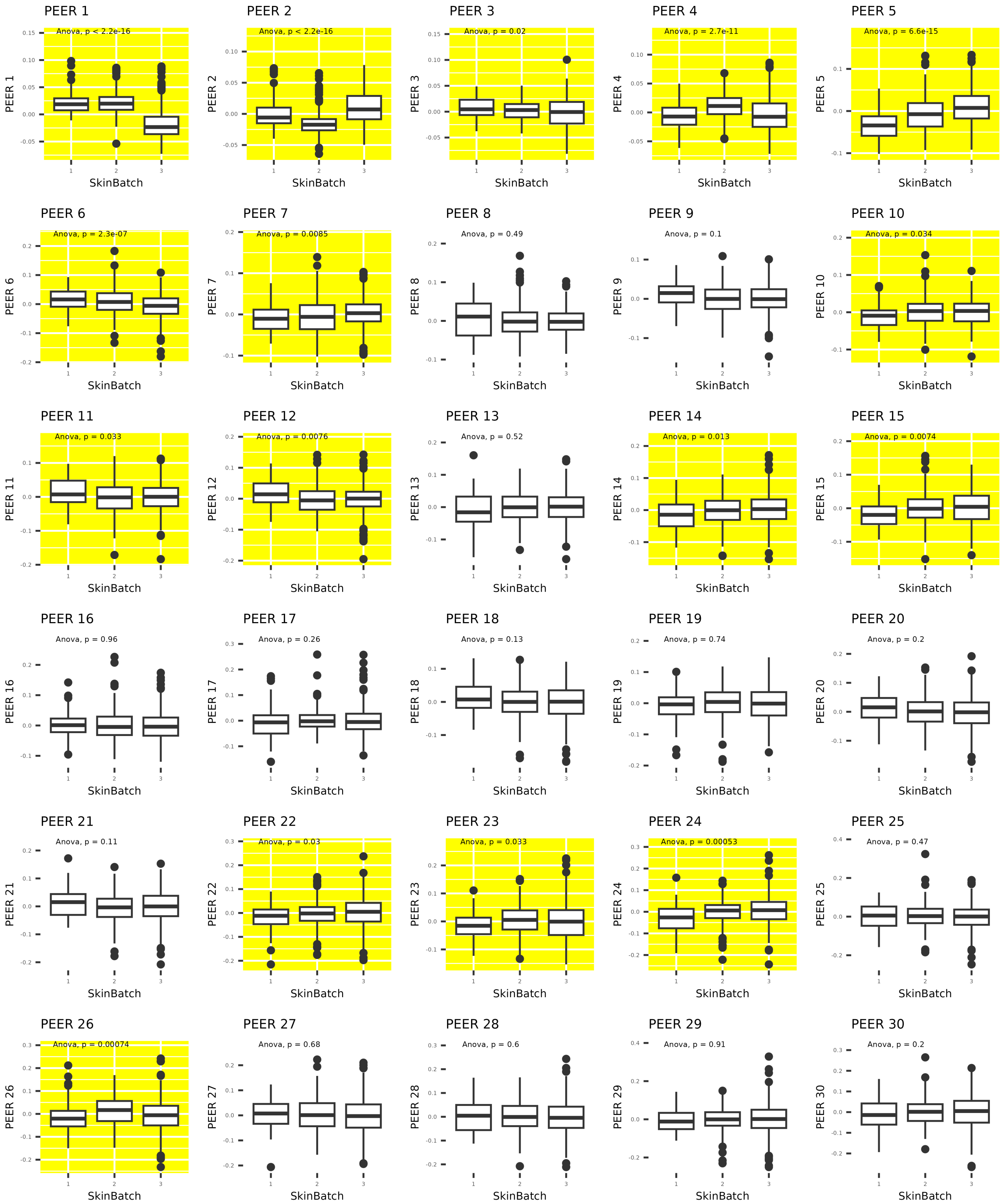

Figure S8. Box plots showing the correlation between the 30 PEER factors used as covariates in the skin eQTL analysis and the RNAseq batch. A yellow background indicates a nominally significant difference between groups (Anova P<0.05).

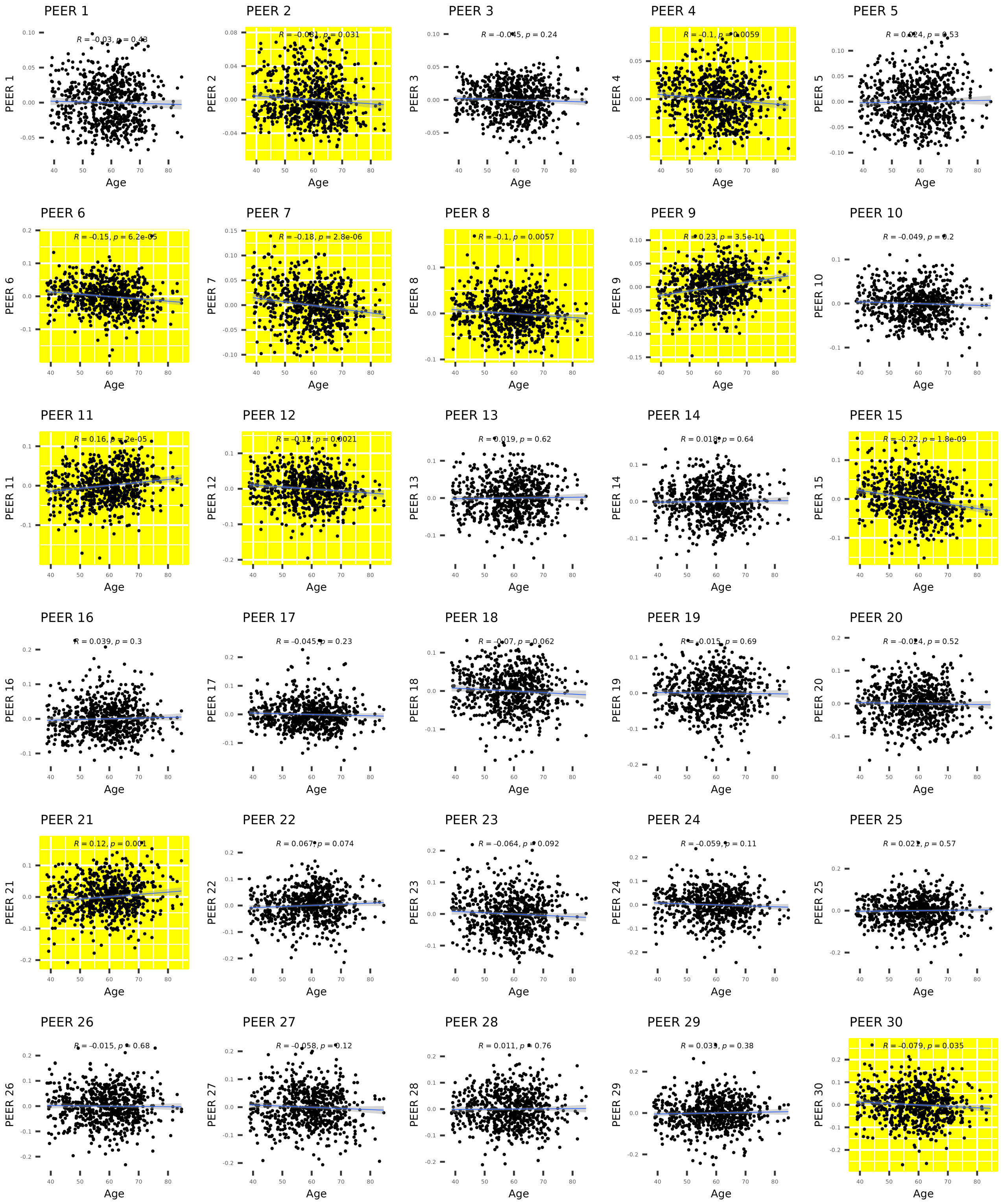

Figure S9. Scatter plots showing the correlation between the 30 PEER factors used as covariates in the skin eQTL analysis and chronological age. A yellow background indicates a nominally significant correlation (Pearson P<0.05).

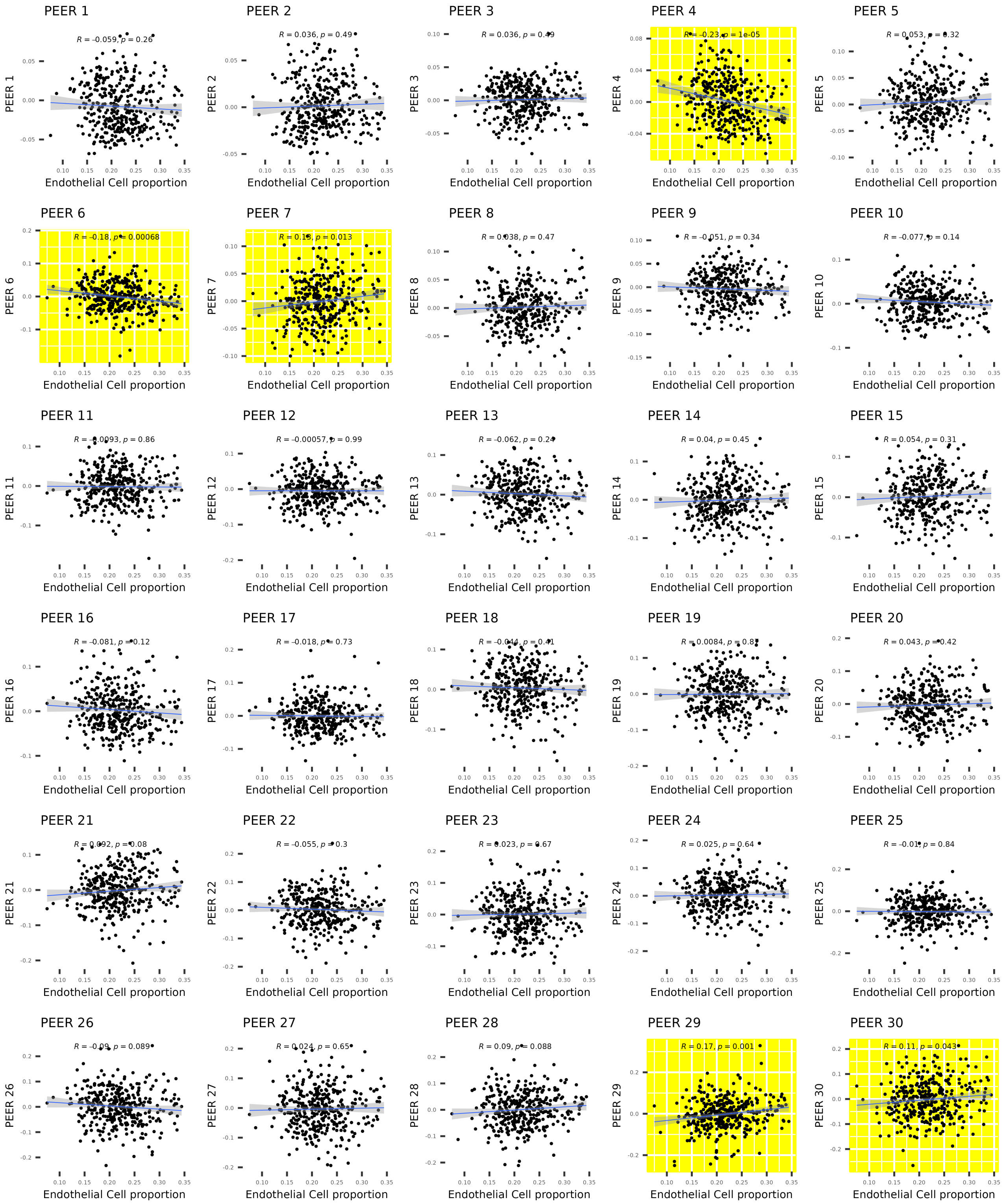

Figure S10. Scatter plots showing the correlation between the 30 PEER factors used as covariates in the skin eQTL analysis and Endothelial Cell proportion as estimated from DNA methylation data. A yellow background indicates a nominally significant correlation (Pearson P<0.05).

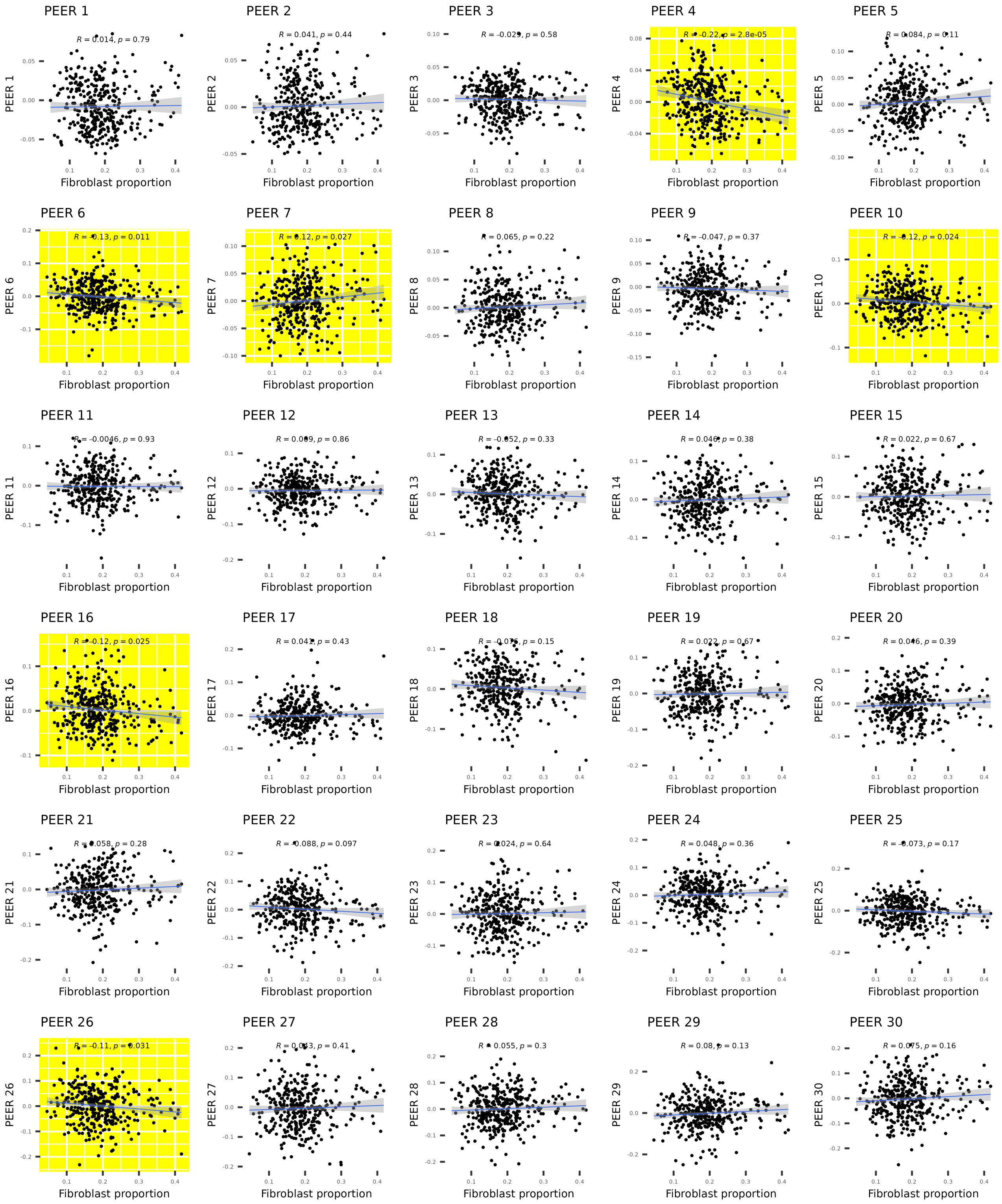

Figure S11. Scatter plots showing the correlation between the 30 PEER factors used as covariates in the skin eQTL analysis and Fibroblast Cell proportion as estimated from DNA methylation data. A yellow background indicates a nominally significant correlation (Pearson P<0.05).

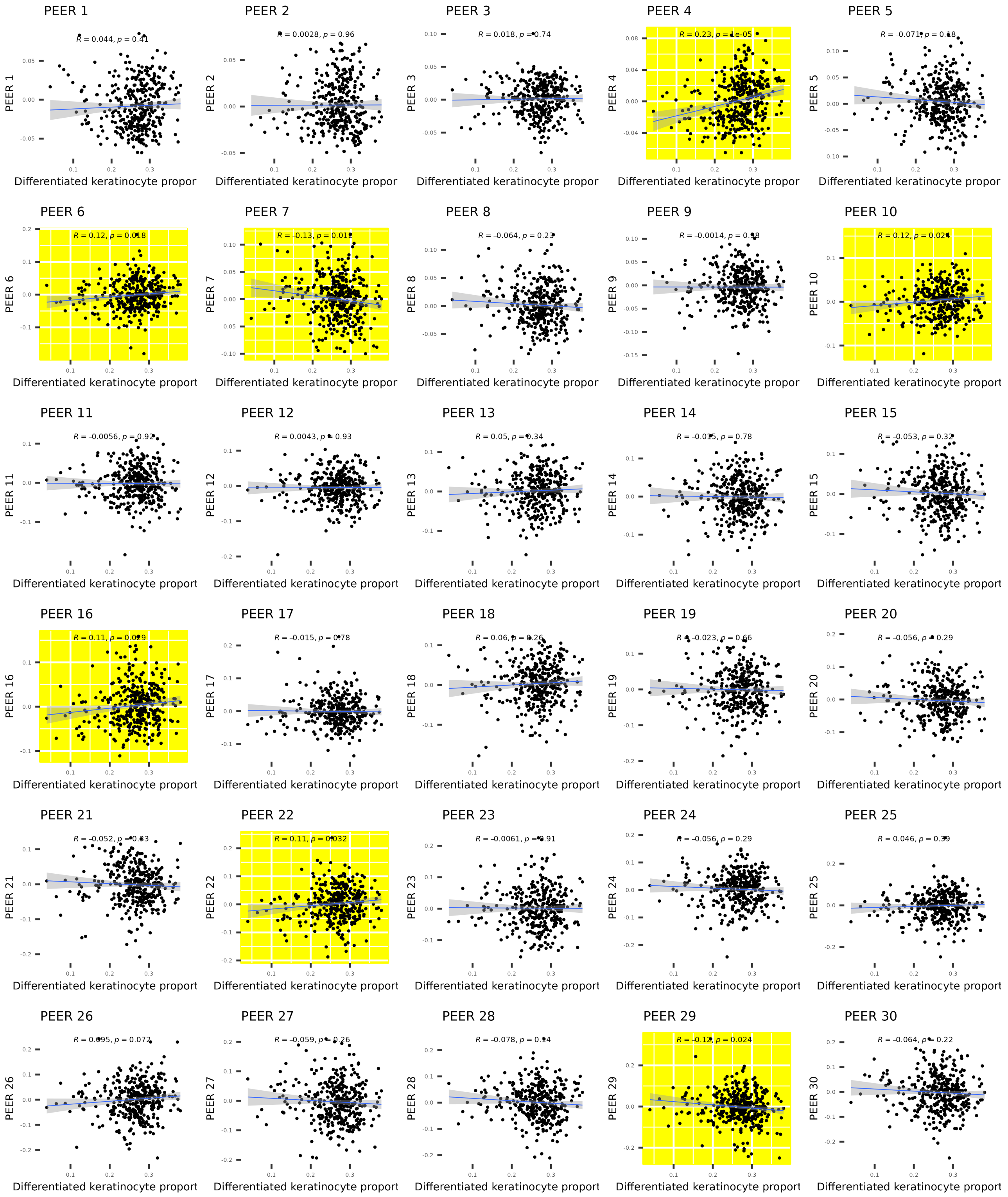

Figure S12. Scatter plots showing the correlation between the 30 PEER factors used as covariates in the skin eQTL analysis and Differentiated Keratinocyte Cell proportion as estimated from DNA methylation data. A yellow background indicates a nominally significant correlation (Pearson P<0.05).

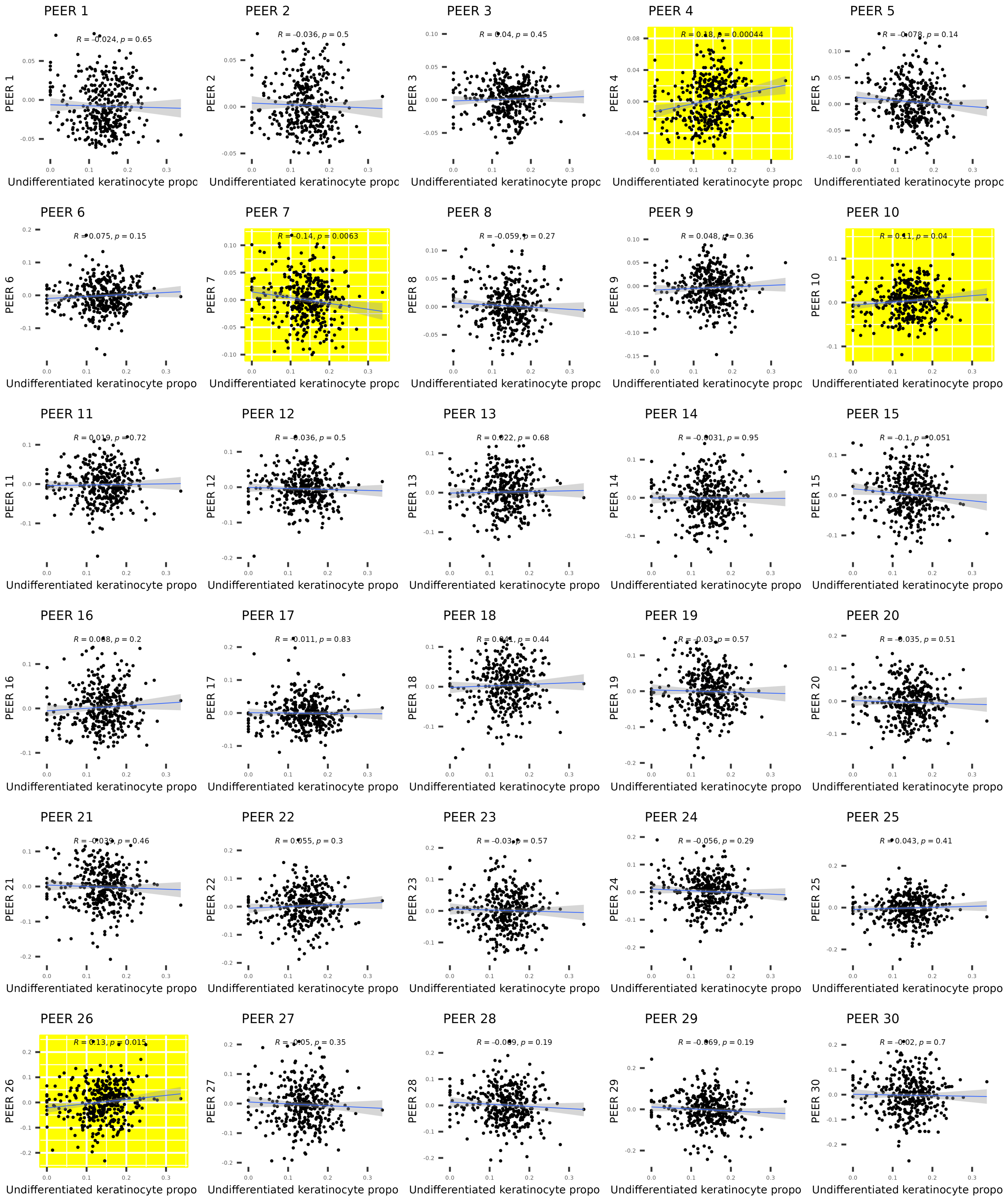

Figure S13. Scatter plots showing the correlation between the 30 PEER factors used as covariates in the skin eQTL analysis and Undifferentiated Keratinocyte Cell proportion as estimated from DNA methylation data. A yellow background indicates a nominally significant correlation (Pearson P<0.05).

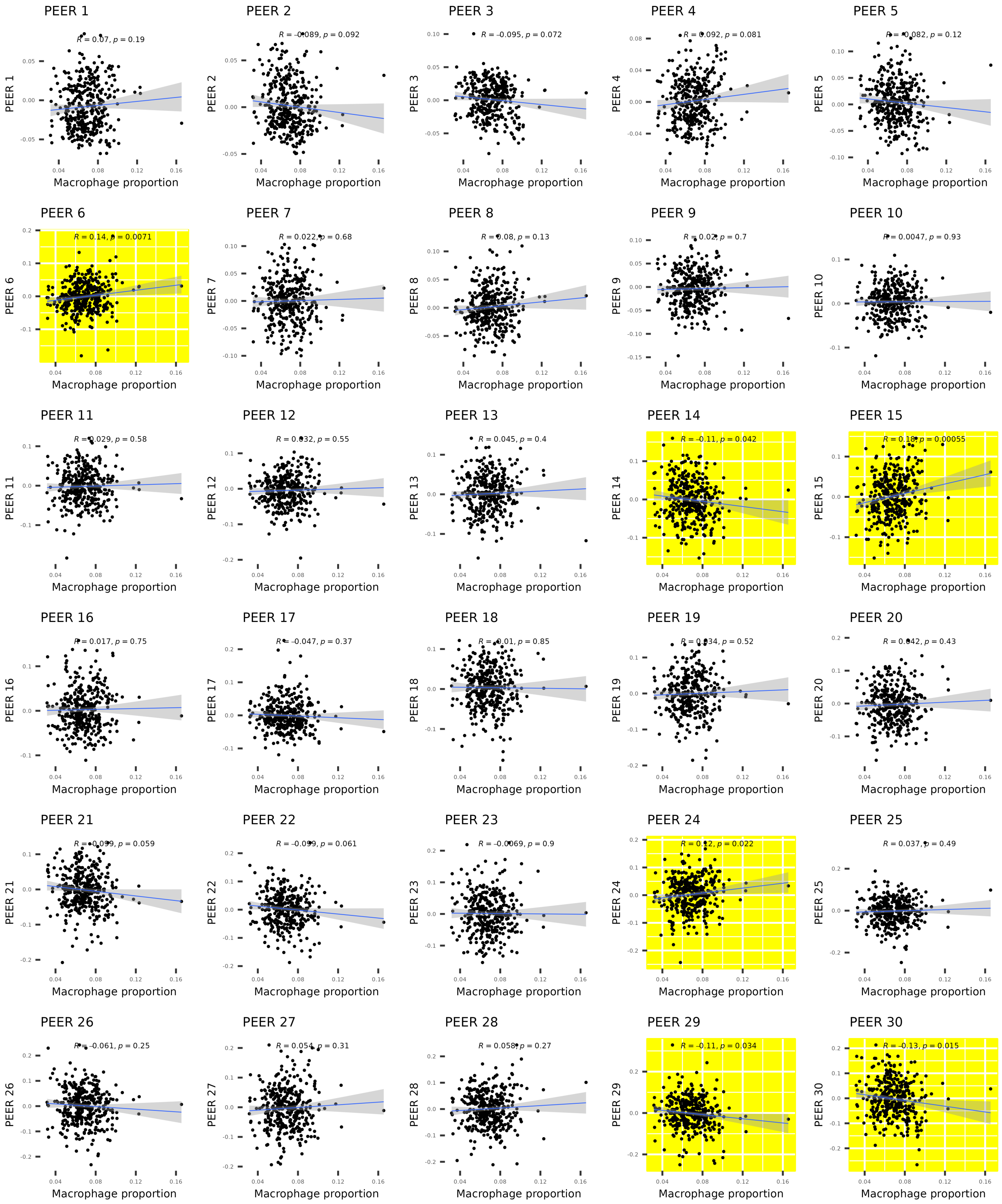

Figure S14. Scatter plots showing the correlation between the 30 PEER factors used as covariates in the skin eQTL analysis and Macrophage Cell proportion as estimated from DNA methylation data. A yellow background indicates a nominally significant correlation (Pearson P<0.05).

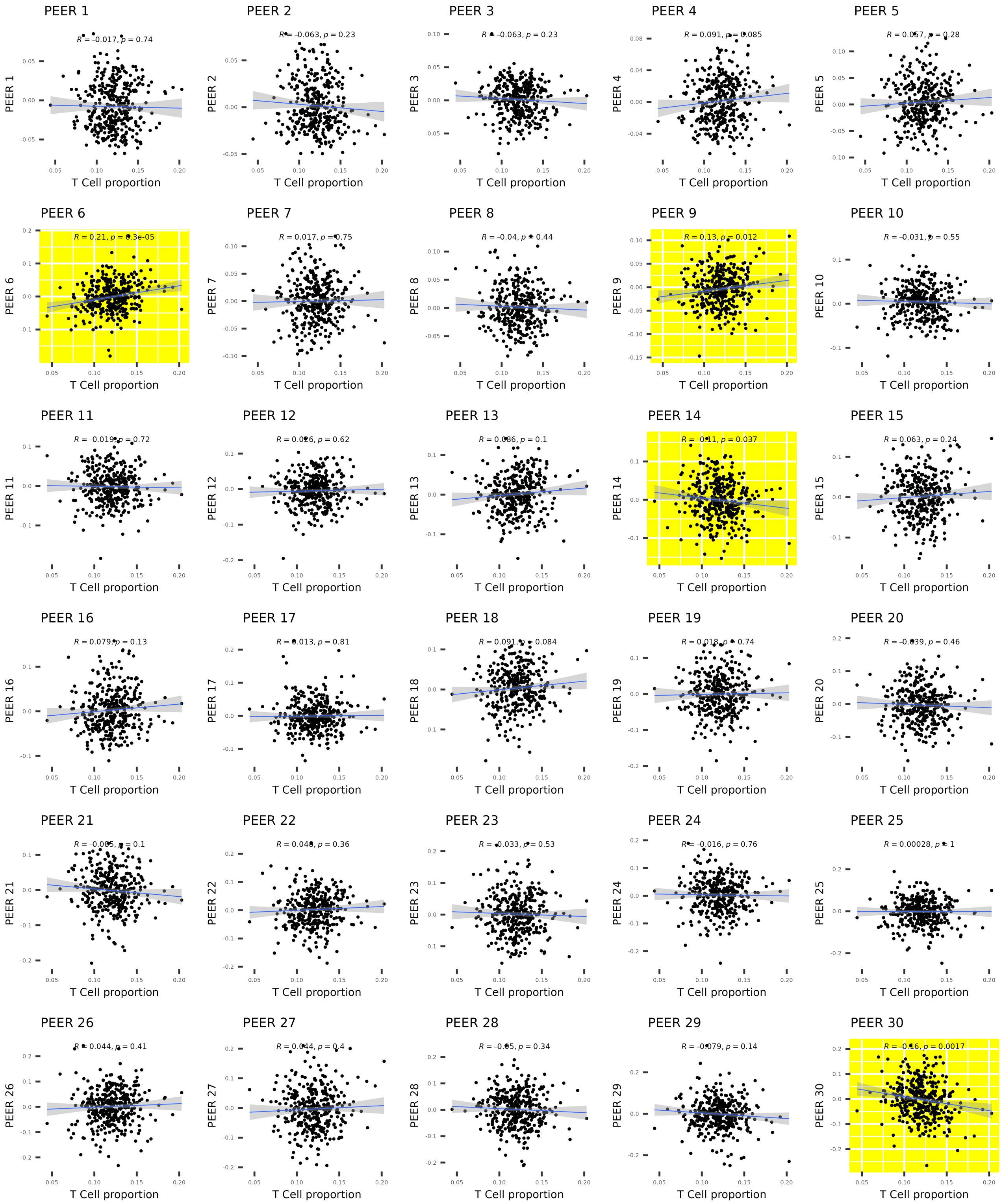

Figure S15. Scatter plots showing the correlation between the 30 PEER factors used as covariates in the skin eQTL analysis and T-Cell proportion as estimated from DNA methylation data. A yellow background indicates a nominally significant correlation (Pearson P<0.05).

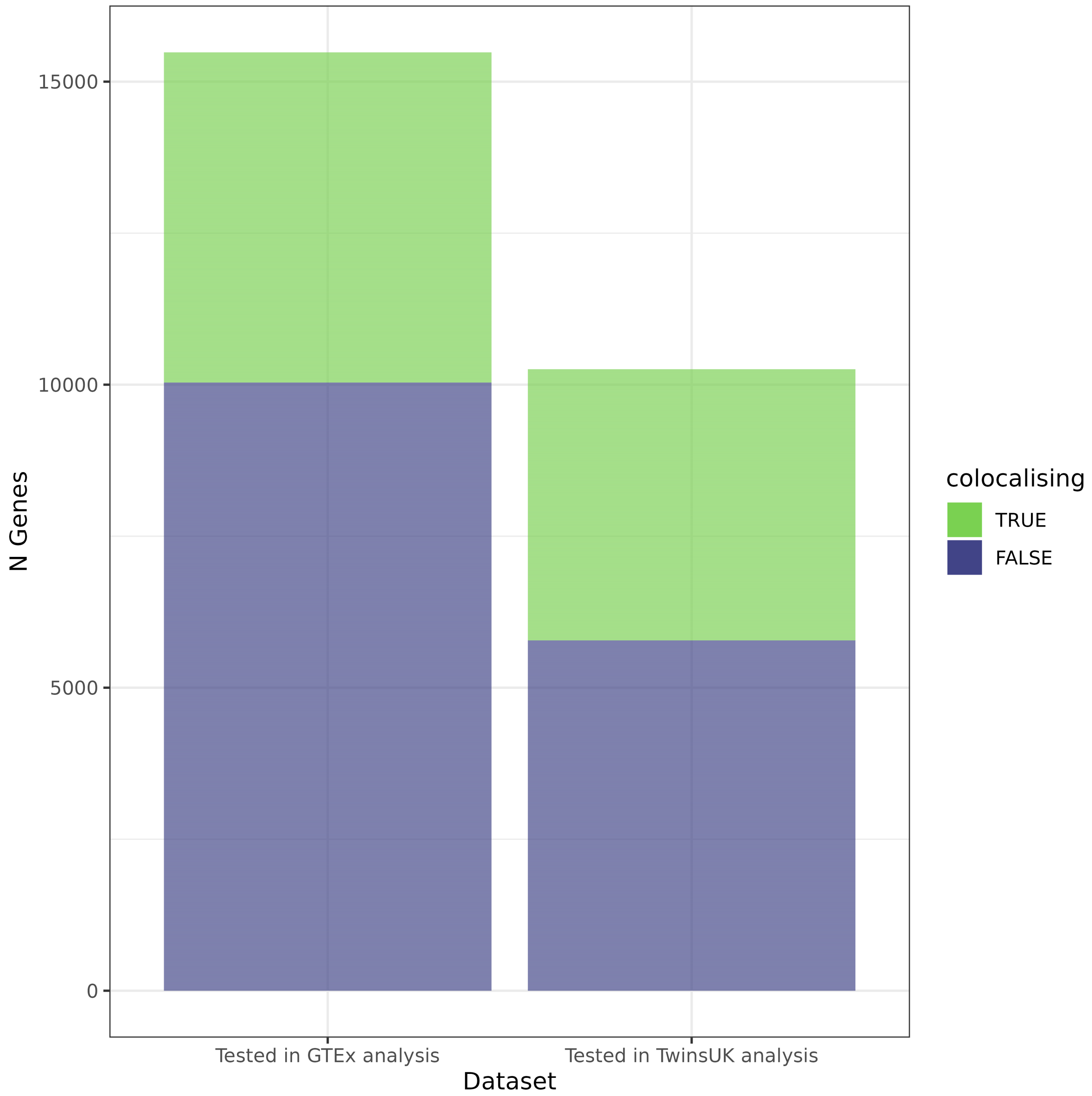

Figure S16. Proportion of eGenes colocalised in both the original meQTL-eQTL colocalisation analysis using TwinsUK data, and the validation analysis using the suprapubic skin GTEx eQTL data with TwinsUK meQTL data.

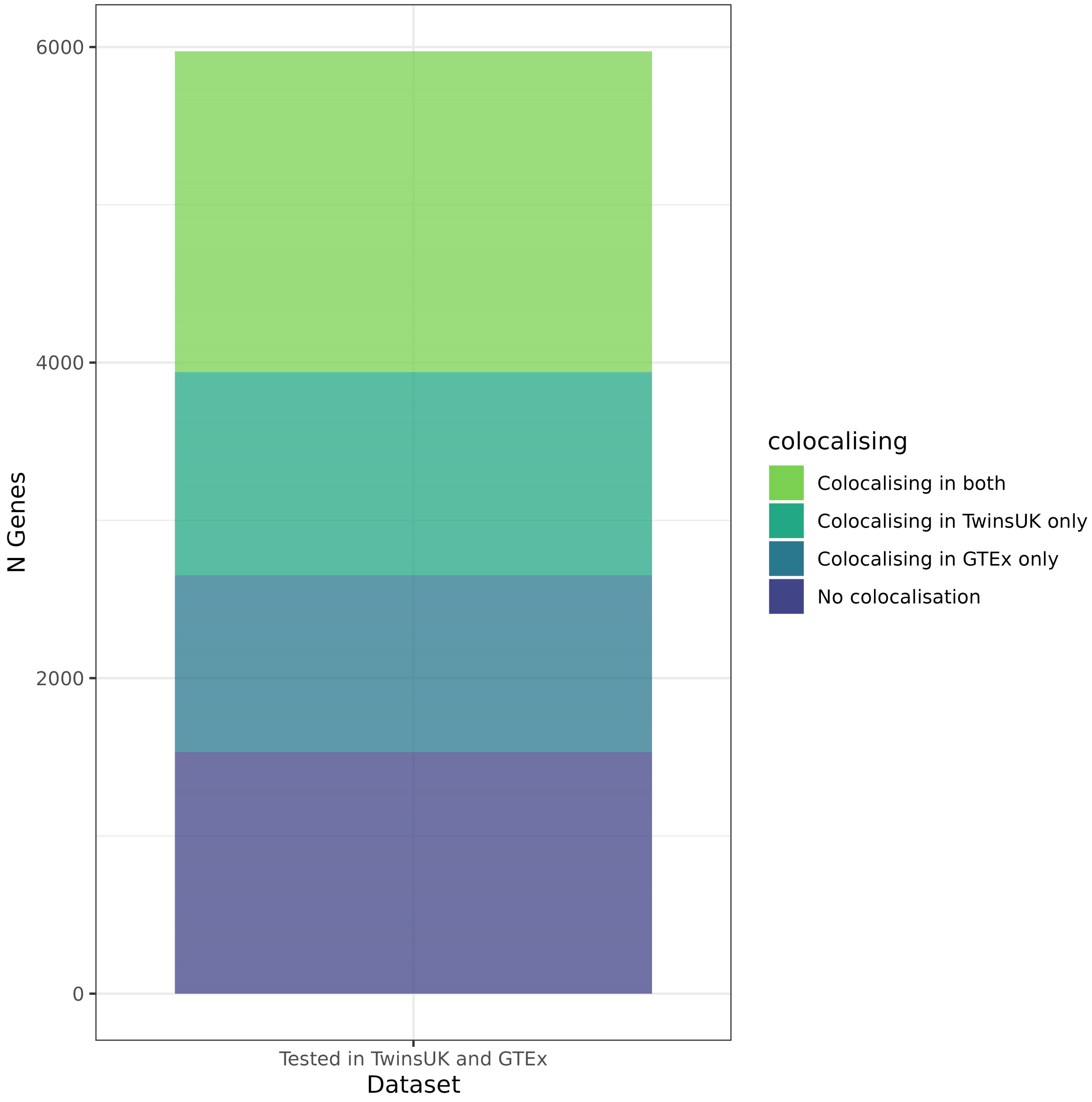

Figure S17. Proportion of eGenes tested in both the original meQTL-eQTL colocalisation analysis using TwinsUK data, and the validation analysis using the suprapubic skin GTEx eQTL data with TwinsUK meQTL data, which colocalise in each analysis.

Supplemental References

[1] Vandiver, A. R., Irizarry, R. A., Hansen, K. D., Garza, L. A., Runarsson, A., Li, X., Chien, A. L., Wang, T. S., Leung, S. G., Kang, S., et al. (2015). Age and Sun Exposure-Related Widespread Genomic Blocks of Hypomethylation in Nonmalignant Skin. Genome Biology. 16, 80.10.1186/s13059-015-0644-y.
[2] Min, J. L., Hemani, G., Hannon, E., Dekkers, K. F., Castillo-Fernandez, J., Luijk, R., Carnero-Montoro, E., Lawson, D. J., Burrows, K., Suderman, M., et al. (2021). Genomic and Phenotypic Insights from an Atlas of Genetic Effects on DNA Methylation. Nature Genetics. 53, 1311–1321.750.10.1038/s41588-021-00923-x.
[3] Guo, H., Fortune, M. D., Burren, O. S., Schofield, E., Todd, J. A., and Wallace, C. (2015). Integration of Disease Association and eQTL Data Using a Bayesian Colocalisation Approach Highlights Six Candidate Causal Genes in Immune-Mediated Diseases. Human Molecular Genetics. 24, 3305–3313.10.1093/hmg/ddv077.
