# Supplementary material for: Genetic effects on the skin methylome in healthy older twins
Source: Am J Hum Genet. 2024 Aug 12;111(9):1932–52. doi: 10.1016/j.ajhg.2024.07.010 (PMC11393713; doi:10.1016/j.ajhg.2024.07.010)
Supplement: Document S2. Article plus supplemental information [file mmc3.pdf]

Genetic effects on the skin methylome in healthy older twins

Authors

Christopher J. Shore, Sergio Villicaña,
Julia S. El-Sayed Moustafa, ..., Tim D. Spector,
Kerrin S. Small, Jordana T. Bell

Correspondence

christopher.shore@kcl.ac.uk (C.J.S.),
jordana.bell@kcl.ac.uk (J.T.B.)

We estimate the heritability of the human skin DNA methylome, followed by identification of specific genetic effects underlying skin DNA methylation levels. We also explored shared genetic effects influencing both DNA methylation and gene expression levels in skin, including in genetic loci linked to skin diseases and phenotypes.

Shore et al., 2024, The American Journal of Human Genetics *111*, 1932–1952
September 5, 2024 © 2024 The Authors.
https://doi.org/10.1016/j.ajhg.2024.07.010

CellPress

ARTICLE

Genetic effects on the skin methylome in healthy older twins

Christopher J. Shore,[1,*] Sergio Villicaña,[1] Julia S. El-Sayed Moustafa,[1] Amy L. Roberts,[1] David A. Gunn,[2] Veronique Bataille,[1] Panos Deloukas,[3] Tim D. Spector,[1] Kerrin S. Small,[1] and Jordana T. Bell[1,*]

Summary

Whole-skin DNA methylation variation has been implicated in several diseases, including melanoma, but its genetic basis has not yet been fully characterized. Using bulk skin tissue samples from 414 healthy female UK twins, we performed twin-based heritability and methylation quantitative trait loci (meQTL) analyses for >400,000 DNA methylation sites. We find that the human skin DNA methylome is on average less heritable than previously estimated in blood and other tissues (mean heritability: 10.02%). meQTL analysis identified local genetic effects influencing DNA methylation at 18.8% (76,442) of tested CpG sites, as well as 1,775 CpG sites associated with at least one distal genetic variant. As a functional follow-up, we performed skin expression QTL (eQTL) analyses in a partially overlapping sample of 604 female twins. Colocalization analysis identified over 3,500 shared genetic effects affecting thousands of CpG sites (10,067) and genes (4,475). Mediation analysis of putative colocalized gene-CpG pairs identified 114 genes with evidence for eQTL effects being mediated by DNA methylation in skin, including in genes implicating skin disease such as *ALOX12* and *CSPG4*. We further explored the relevance of skin meQTLs to skin disease and found that skin meQTLs and CpGs under genetic influence were enriched for multiple skin-related genome-wide and epigenome-wide association signals, including for melanoma and psoriasis. Our findings give insights into the regulatory landscape of epigenomic variation in skin.

Introduction

DNA methylation of cystosine residues at CpG dinucleotides is one of the most well-studied epigenetic DNA modifications. The number of diseases and phenotypes associated with changes in DNA methylation highlights the importance of understanding both the sources of DNA methylation variation and the biological impact of DNA methylation changes.

Although changes in DNA methylation have been attributed to a plethora of environmental factors, heritability and quantitative trait locus (QTL) analyses show a substantial genetic contribution to variation in the DNA methylome. Indeed, twin-based heritability studies of DNA methylation in whole blood show that the majority of DNA methylation variation in >10% of measured CpG sites is attributable to narrow-sense heritability.[1] More recently, a study by the Genetics of DNA Methylation Consortium (GoDMC) performed DNA methylation QTL (meQTL) analyses in blood samples from more than 32,000 participants and found that over 45% of all tested CpG sites were associated with at least one meQTL,[2] emphasizing the impact of genetic effects on the DNA methylome. These results are in line with other genetic studies of the blood methylome, both based on the Illumina Infinium HumanMethylation 450 BeadChip Array (Illumina 450k) platform, assaying 2% of the DNA methylome,[2–11] and more recently on the Illumina Infinium MethylationEPIC BeadChip platform, assaying 3.7% of the DNA methylome.[12,13] meQTLs have also been found to overlap with other molecular QTLs, including expression QTLs (eQTLs). Some meQTL studies have explored this further, showing evidence that eQTL effects can be mediated by meQTLs and vice versa.[14–16] In some cases, there is evidence to suggest such mediated effects are involved in disease mechanisms and may explain genetic variant-disease associations identified in genome-wide association studies (GWASs).[4,14–17]

Although the largest meQTL studies to date have been carried out in blood, meQTL studies in disease-relevant tissues identify substantial tissue-specific meQTL effects, for example, in heart, adipose, lung, ovary, kidney, prostate, cerebellar, cortical, and pons tissues.[6,8–11,18–21] As an example, Schulz et al.[7] found that 66% of hippocampal *cis*-meQTL effects were replicated in whole blood. Similarly, Min et al.[2] found that blood *cis*-meQTL effects were only partially shared with adipose and brain *cis*-meQTL effects ($r_b = 0.73$ and 0.59, respectively) while Lin et al.[22] found that CpGs associated with meQTLs in pre-frontal cortex, saliva, and blood samples showed overlaps of only 31%–68%.

Skin plays a vital role in human health, acting as a protective barrier against infection, physical and chemical injury, preventing loss of moisture, and providing sensory inputs of touch and heat. Thus, when these functions are impaired by disease, injury, or aging, there can be systemic effects on the body. DNA methylation in skin has been associated with numerous traits such as melanoma risk,[4,23] nevus count,[24] and skin aging markers,[25–28] all of which have also been associated with genetic variants in

[1]Department of Twin Research and Genetic Epidemiology, King's College London, London, UK; [2]Unilever R&D, King's College London, London, UK; [3]William Harvey Research Institute, Barts and the London School of Medicine and Dentistry, Queen Mary University of London, London, UK
*Correspondence: christopher.shore@kcl.ac.uk (C.J.S.), jordana.bell@kcl.ac.uk (J.T.B.)
https://doi.org/10.1016/j.ajhg.2024.07.010.
© 2024 The Authors. This is an open access article under the CC BY license (http://creativecommons.org/licenses/by/4.0/).

multiple GWAS.[29–34] Greater understanding of the genetic basis of the skin methylome may improve our knowledge of the mechanisms underlying skin traits and may potentially reveal new therapeutic targets for the treatment of skin diseases and attenuation of skin aging. However, although meQTL effects have been detected in primary melanocyte and melanoma samples,[4] to date, no comprehensive genome-wide analyses of DNA methylation heritability or meQTL analyses have been performed in whole skin. Changes in skin DNA methylation associated with nevus count have been linked to targeted genetic variants, and meQTL effects involving genetic variants linked to melanoma biology have been identified in melanocytes, warranting further exploration of such genetic impacts on the human skin methylome at genome-wide resolution.[4,24]

To better understand the processes underlying DNA methylation variation in skin, we explored the genetic basis of the skin methylome using a 2-fold approach. First, we performed genome-wide heritability analyses of skin DNA methylation on the Illumina 450K array using a twin-based study design. Secondly, we carried out genome-wide meQTL detection in skin tissue, identifying tens of thousands of meQTL-CpG associations. Follow-up analyses explored the genomic distribution of these meQTL effects and their colocalization with eQTLs to assess evidence for shared genetic basis of the skin methylome and transcriptome. We also considered the impact of skin meQTLs on human skin phenotypes by integrating our results with previous epigenome-wide association studies (EWASs) and GWAS findings for skin-related phenotypes. Our findings provide genome-wide identification of a robust effect of genetic variation on the whole-skin methylome with insights for human health and disease.

Material and methods

Sample and phenotype collection

Skin DNA methylation and gene expression profiles were explored in 414 and 706 female twins, respectively (overlap = 361), from the TwinsUK Adult Twin Registry.[35] The sample consisted of predominantly older (mean age of 59, age range of 38–84) female individuals of self-reported European ancestry, which informed choice of imputation reference panel and validation datasets. DNA methylation was profiled in 70 paired monozygotic (MZ) twins, 140 paired dizygotic (DZ) twins, and 204 singletons. Gene expression was profiled in 220 paired MZ twins, 336 paired DZ twins, and 150 singletons. These individuals were not selected for disease, and the TwinsUK cohort has representative means and ranges of quantitative phenotypes to an age-matched population in the UK.[36]

Sample collection, DNA methylation profiling, and gene expression profiling have previously been described for subsets of these data.[24,37] In brief, punch biopsies (8 mm) were taken from a relatively photo-protected region adjacent and inferior to the umbilicus, and fat tissue was mechanically separated from the skin biopsy. DNA and RNA were extracted from the whole-skin tissue section, and DNA methylation and gene expression profiling were then performed. Written informed consent was obtained, and the procedures were in accordance with the ethical standards of the St. Thomas' Research Ethics Committee (REC reference 07/H0802/84).

Genome-wide skin DNA methylation profiles

DNA samples were extracted from the skin biopsies as previously described,[24] and bisulfite converted in preparation for DNA methylation profiling. Genome-wide DNA methylation profiling of the bisulfite converted skin tissue DNA samples was carried out using the Illumina Infinium HumanMethylation450 BeadChip (Illumina, San Diego, CA). At each CpG-site, the resulting DNA methylation levels were quantified as beta-values, which at an individual CpG site represents the ratio of intensity signal from the methylated probes over the sum of intensity signals from both unmethylated and methylated probes plus 100. Multiple measurements of analytical quality were then applied. Probes were removed from downstream analysis if they failed detection in at least one sample or had a bead count less than 3 in more than 1% of the samples. Multi-mapping probes were removed, as well as those recommended for exclusion by Zhou, Laird, and Shen,[38] including probes overlapping common SNPs in dbSNP (minor allele frequency [MAF] > 1%).

A total of 407,348 CpG probes were retained for downstream analyses.

Sample identity was verified by comparing genotype data to genotypes estimated from the 57 autosomal SNP probes included as control probes on the Illumina 450K chip. Overall intensity signal and bisulfite conversion efficiency were assessed, and the data were inspected visually for outliers using beta density plots generated in ENmix.[39] Overall, 414 samples passed quality control assessment and were then normalized using the Regional Regression on Correlated Probes with quantile normalization algorithm to correct for probe-type bias and reduce technical variation.[40]

Skin-cell-type composition estimates were generated using the EpiSCORE R package.[41,42] We used the EpiSCORE skin-cell-type DNAm reference matrix included in the package to obtain skin-cell composition estimates for fibroblasts, endothelial cells, macrophages, T cells, melanocytes, and differentiated and undifferentiated keratinocytes. Melanocyte cell-type proportion estimates were null in every sample and thus excluded from future analyses.

Principal component analysis (PCA) was performed on the normalized methylation betas after removal of the X and Y chromosome probes and rank-based inverse normal transformation (to N[0,1]) at each probe using the PCAtools R package (https://github.com/kevinblighe/PCAtools). The first 10 principal components (PCs), together explaining

34% of the total methylation variance, were retained and tested for associations with potential covariates. Covariates included age, BMI, smoking status, skin-cell-type composition estimates, chip, position on chip, median intensity signal, batch, and bisulfite conversion efficiency. Robust associations ($p < 0.001$) with PCs were identified for fibroblast, endothelial cell, macrophage, T cell, and differentiated and undifferentiated keratinocyte cell proportions, as well as BMI, chip, batch, and bisulfite conversion efficiency.

Skin gene expression profiles

Skin gene expression profiling has been described previously.[37,43] TwinsUK skin RNA-seq data are deposited in the European Genome-phenome Archive under EGA: EGAS00001000805. In brief, the Illumina TruSeq sample preparation protocol was used to generate the cDNA libraries for sequencing, and the Illumina HiSeq 2000 machine was used to generate 49 bp paired-end reads. Samples were removed if they failed library preparation, had < 10 million reads, or had sequence data that did not match directly genotyped data (where available). RNA-seq reads were then aligned to an hg19 reference genome using STAR[44] version 2.4.0.1, and gene-level quantification was performed using the QTLtools quan function[45] and Gencode version 19.[46]

The gene expression data were filtered to only include genes with 5 or more counts per million (CPM) in at least 25% of samples, and gene counts were transformed to trimmed mean of M-values (TMM). TMM-transformed gene CPMs were then inverse-normalized prior to downstream analyses. In total, quantifications of 23,838 genes in 706 individual whole-skin samples were available for downstream analysis.

Genotypes

TwinsUK genotype data were available for 394 of the 414 individuals with skin DNA methylation profiles and for 664 of the 706 of the individuals with skin gene expression profiles. Genotyping was performed as previously described.[47] Briefly, samples were genotyped using a combination of Illumina HumanHap300, HumanHap610Q, 1M-Duo, or 1.2M-Duo custom arrays, and the normalized intensity data for each array were pooled separately. The Illuminus calling algorithm was then used to assign genotypes in the pooled data.[48] The HRC/1KG Imputation Preparation and Checking Tool (version 4.2.5) was used to check input data for accuracy relative to expected Haplotype Reference Consortium (HRC) or 1000 Genomes Project (1000G) inputs prior to imputation, and identified errors (including incorrect ref/alt allele designations, incorrect strand designations, extreme deviations from expected allele frequencies, and palindromic SNPs with allele frequencies near 0.5) were removed or corrected. Imputation was performed on the Michigan Imputation Server with reference panel HRC r1.1 2016, Eagle v2.3 phased output, EUR population, and quality control and imputation mode.

Where one twin in an MZ pair was not genotyped, duplicate genotype data from their co-twin was used. Variants were filtered for MAF (>0.05), imputation quality (info score > 0.8), Hardy-Weinberg equilibrium ($p > 1 \times 10^{-6}$), and minimum genotyping rate (>95%). Additionally, only autosomal single-nucleotide polymorphism (SNP) variants were retained. Altogether, 5,253,496 SNPs were included in the downstream meQTL analyses, and 5,275,301 SNPs were included in the downstream eQTL analyses. We report all analysis results using the GRCh37/hg19 reference genome. Individual-level genotype data can be applied for through the TwinsUK data access committee (https://twinsuk.ac.uk/resources-for-researchers/access-our-data/).

Estimating the heritability of the skin methylome

We assessed the narrow-sense heritability of skin DNA methylation at each measured CpG site by use of the twin-based heritability model. The model estimated the proportion of variance attributable to additive (or narrow sense) heritability (a^2 or A), common (shared between twins in a pair) environmental effects (c^2 or C), and unshared (between twins in a pair) environmental effects (e^2 or E). Because this ACE model generates estimates using pairs of MZ twins and DZ twins, we excluded singletons from this analysis, leaving 70 paired MZ and 140 paired DZ twins for the ACE model. We used the R package OpenMX[49] to construct an ACE model for each CpG site, and report the maximum likelihood estimates of the best fitting values for A, C, and E for the methylation. We assumed equal means and variances between zygosity groups.

Identification of skin meQTLs

In estimating meQTLs in skin tissue, we followed the meQTL discovery pipeline outlined by GoDMC[2] (https://github.com/MRCIEU/godmc) where possible. To this end, we first generated a pedigree genetic relatedness matrix (GRM) using the 394 imputed genotypes. Using a subset of common HapMap3 SNPs (MAF > 0.2) that excluded long-range linkage disequilibrium (LD) regions, we calculated the first 20 genetic PCs using the R package GENESIS.[50] The PCA results confirm that none of the 394 individuals were ancestry outliers (>7 SDs from the mean for any of the first 20 genetic PCs).

We then carried out a number of control checks and adjustments on the DNA methylation data. First, we explored DNA methylation outliers. For each probe, we removed DNA methylation values that were outliers (>10 SDs from the mean, performed in three iterations). The remaining methylation values were rank-based inverse normal transformed at each probe in downstream analysis. Second, to reduce non-genetic variation in the DNA methylation data as much as possible, the normalized methylation values at each probe were then fit against age, BMI, chip, position on chip, and family relatedness (estimated from the pedigree GRM) in a linear mixed model (LMM) using the R package GenABEL version 1.851.[51] Rank-based

inverse normal transformed residuals from this linear model were retained. All methylation values removed as outliers in the previous step were then set to the probe mean. Third, a PCA was performed on the most variable 20,000 probes from the adjusted methylation data. GWASs were then performed for the first 20 methylation PCs. None of the first 20 methylation PCs were under strong genetic effects ($p < 1 \times 10^{-7}$). Therefore, all 20 were retained as further potential covariates. Fourth, the methylation residuals from the second step were then regressed on to the 20 methylation PCs. The resulting residuals were retained and rank-based inverse transformed to N(0,1). These adjusted methylation data were then directly used in the downstream meQTL analysis.

We used the R package MatrixEQTL version 2.3[52] to perform association analyses between all retained adjusted methylation residuals and genetic variants using a linear model. We retained all associations in *cis* (variant <1 Mbp from CpG site) that surpassed a nominal p value threshold of $p < 1 \times 10^{-3}$ and all associations in *trans* (>1 Mbp from CpG site or different chromosome) that surpassed a nominal p value threshold of $p < 1 \times 10^{-5}$. Association regression coefficients are given with respect to the minor allele. To correct for multiple testing, we estimated genome-wide false discovery rate (FDR) thresholds using a permutation approach. We performed 20 genome-wide permutations of the genotype data where twin pairs were shuffled together within zygosity groups. We then estimated the *cis* and *trans* FDR thresholds using the best *cis* or *trans* association for each CpG probe in the observed association analysis and across all permutations. The resulting permutation-based genome-wide FDR 5% thresholds were estimated for both *cis* (nominal $p <$ 1.67e-5) and *trans* (nominal $p < 8.17 \times 10^{-11}$) results.

LD across genetic variants can generate redundant associations with variants in LD with true meQTLs. To identify true causal genetic variants, we also performed conditional analyses for each CpG probe. This was carried out using the –cojo-slct function in GCTA.[53,54] GCTA –cojo-slct uses a stepwise model selection procedure to select independently associated variants based on strength of association. Conditional analysis indicated that 414 detected *trans* associations were in fact confounded by LD with *cis* associations.

Genomic and functional enrichment of meQTL-CpG pairs

We explored whether meQTLs and associated CpG sites were more or less likely to fall in specific genomic regions. To this end, we used BEDTools version 2.27.1[55] and CpG island and UCSC refGene annotations from UCSC[56] to generate annotations for CpG shores (2 kbp regions flanking CpG islands), CpG shelves (2 kbp regions flanking CpG shores), and open seas (any part of the genome > 4 kbp from a CpG island). RefGene annotations were used for exons only, whole gene bodies, 3′ untranslated regions (UTRs), 5′ UTRs, and regions 200 and 200–1,500 bp upstream of transcription start sites (TSS200 and TSS1500 regions, respectively). We also created annotation only for the first exon of each gene, as DNA methylation in first exons has been linked to regulation of gene expression.[57] We tested for enrichment and depletion of meQTLs and CpGs associated with meQTLs in each annotation category using an adaptation of the R software package LOLA.[58] Our approach uses LOLA output to perform a Fisher's exact test for significant enrichment or depletion of features of interest (i.e., meQTLs or CpGs associated with meQTLs) in different genomic regions when compared to a set of background features (i.e., all tested genetic variants and all tested CpG sites).

We also explored the distribution of meQTL-CpG pairs in different chromatin state regions in skin. To this end we used annotation tracks for chromatin states predicted using an 18-state ChromHMM model (from the Roadmap Epigenomics Consortium[59]) in leg skin from the EpiMap repository.[60] We used LOLA to test for enrichment or depletion of meQTLs and CpGs associated with meQTLs in these regions compared to a background of all tested genetic variants and CpGs, respectively.

Transcription factor binding at transcription factor binding sites (TFBSs) could represent both a mechanism of meQTL effects and mechanisms by which meQTL effects might alter gene expression phenotypes. We used annotations for CTCF and POL2RA binding from chromatin immunoprecipitation followed by sequencing (ChIP-seq) experiments in two female suprapubic skin samples (ENCODE: ENCBS825XXY and ENCBS296VML) from the EpiMap repository.[60] We merged bed files from each sample for each transcription factor using BEDTools version 2.27.1,[55] and enrichment analyses for regions with CTCF and POL2RA binding were performed using LOLA as described previously for enrichment in ChromHMM state annotated regions.

Enrichment of meQTL effects in skin disease and trait loci

We explored the enrichment of CpGs associated with meQTLs in EWASs. Association statistics for EWAS signals were downloaded from the EWAS Catalog[61] and the EWAS Atlas.[62,63] We filtered for studies of skin-related diseases and traits (Tables S2 and S3) with at least five signals present in our whole-skin DNA methylation data. The associations from the EWAS Catalog and EWAS Atlas were then merged into a single list for each trait, and trait names were harmonized (e.g., "age," "Age," and "aging" were set to "Aging"). Fisher's exact tests were used to assess the enrichment of *cis*- and *trans*-CpGs in each trait compared to a background of all tested CpGs. Significant enrichment was defined as an odds ratio > 1, Benjamini-Hochberg FDR < 0.05, and at least five EWAS signals in the tested set of CpGs.

The enrichment of meQTLs in GWAS results was also tested. We obtained association statistics from the GWAS Catalog[64] and filtered for studies of skin-related diseases

(Table S4) and traits with at least five significant signals present in our imputed genotype data. Fisher's exact tests were used to assess the enrichment of all variants associated with a CpG in *cis* or *trans* in each trait compared to a background of all variants that were not associated with DNA methylation in *cis* or *trans*, respectively. Again, significant enrichment was defined as an odds ratio > 1, Benjamini-Hochberg FDR < 0.05, and at least five GWAS signals in the tested set of genetic variants.

Tissue specificity of skin meQTL effects

We investigated the specificity of the whole-skin meQTL effects by comparing our results to blood meQTL effects from Min et al.[2] and to melanocyte meQTL effects from Zhang et al.[4] For *cis*-meQTL effects, we used the π_1 statistic[65] to estimate the validation rate of blood *cis*-meQTLs in whole skin, as well as to estimate the validation rate of whole-skin *cis*-meQTLs in melanocytes. Because calculating the π_1 statistic would not be feasible for *trans*-meQTL effects, we used the number of overlapping significant effects in both datasets to estimate the validation rate of whole-skin *trans*-meQTL effects in blood and melanocytes. In addition, we estimated the correlation of skin *cis*- and *trans*-meQTL effects with available overlapping *cis*- and *trans*-meQTL effects in blood using the r_b statistic.The r_b correlation statistic accounts for error in the estimation of meQTL effects, reducing under-estimation of the true correlation of meQTL effects.[66] Effect alleles from each effect in each dataset were harmonized prior to analyses.

Identification of local skin eQTLs

We explored local eQTL effects in whole-skin gene expression in 664 twin samples. We first aimed to minimize the effect of non-genetic variation in our skin gene expression data. To this end, we fit the TMM-transformed expression estimates (CPM) of each gene to an LMM with BMI as a fixed effect and family and zygosity as random effects. The rank-normal transformed residuals from this regression were then used to generate 50 probabilistic estimation of expression residuals (PEER) factors.[67] These PEER factors represent hidden factors underlying variability in the expression data not related to BMI or family structure. In downstream analyses, we used these PEER factors with no BMI or family-structure effects.

We next sought to determine the number of PEER factors to include as covariates in the eQTL analysis such that eQTL discovery would be maximized while avoiding over fitting the model using an approach similar to that used by Aguet et al.[68] We first generated a separate set of rank-normal transformed residuals by fitting TMM-transformed expression estimates (CPM) to an LMM with family and zygosity as random effects. BMI was not included as a covariate in this second model. Using these residuals for 664 samples with matching genotype data, we performed seven nominal-pass *cis*-eQTL analyses using QTLTools[45] version 1.2, increasing the number of PEER factors included as covariates in each analysis. Genotyping chip and BMI were included as covariates in all seven analyses. We then observed the number of discovered genes with eQTLs (eGenes) at a Benjamini-Hochberg FDR of 5% in each of the analyses. We determined that including the first 30 PEER factors as covariates in our primary eQTL analysis would be optimal (Figure S4).

The 30 PEER factors to be included as covariates in our primary eQTL analysis were explored for associations with technical factors, age, and cell-type proportions (estimated from DNA methylation data). Mean guanine-cytosine (GC) content was found to correlate strongly with the first five PEER factors and with five more of the first 30 factors (Figure S7) and batch effects correlated with 16 PEER factors (Figure S8). Age was found to correlate significantly, albeit weakly with 11 PEER factors (Figure S9). In a subset of the data ($n = 361$), DNAm-estimated cell-type proportions showed significant but weak correlations with up to seven of the first 30 PEER factors (Figures S10–S15). We concluded that these 30 PEER factors captured a substantial proportion of non-genetic variability in the gene expression data.

We then performed eQTL association testing of all genetic variant-gene pairs in *cis* (variant < 1 Mbp from gene start site) by linear regression using QTLtools[45] version 1.2, including BMI, genotyping chip, and 30 PEER factors as covariates. Genome-wide FDR 5% significance was estimated using QTLtools, where for each gene the empirical significance of the most associated variant was calculated using a beta distribution fitted to 1,000 permutations, as described in Delaneau et al.[45] These empirical *p* values were then corrected for multiple testing using the Storey-Tibshirani procedure[65] and used to identify gene-level FDR 5% thresholds. All variant-gene associations with *p* values below this gene-level threshold were considered significant *cis*-eQTL effects. Association beta values are given with respect to the minor allele.

As with meQTL effects, LD structures can cause redundant associations between genetic variants in LD with true eQTLs. We again used the –cojo-slct function in GCTA[53,54] to perform conditional analysis, identifying independent eQTL effects in skin.

Sharing of skin meQTLs and skin eQTLs

We performed colocalization analyses to determine if skin *cis*-meQTLs and proximal skin *cis*-eQTLs shared causal variants. We paired each *cis*-eGene with any *cis*-CpG that was significantly associated with any lead eQTL variants for that gene in *cis*, resulting in a list of 83,529 gene-CpG pairs, including 8,200 *cis*-eGenes and 25,037 *cis*-CpGs. We then flipped all eQTL effect betas and alleles when the effect allele was different between the eQTL and meQTL analyses. Each of the 83,529 gene-CpG pairs was then tested for colocalization of the meQTL and eQTL effects using the coloc R package.[69] Because a substantial proportion of *cis*-eGenes and *cis*-CpGs were associated with multiple distinct QTLs, we used the Sum of Single Effects (SuSiE) coloc method[70] to relax the assumption of a single causal variant.

Prior probabilities for a variant being associated with just the eGene (*p1*), just the *cis*-CpG (*p2*), or with both (*p12*) were chosen using the method outlined by Pierce et al.[14] and Guo et al.[71] Briefly, the prior probability of a variant being associated with the eGene ($p1 + p12$) was set to 0.0026 (i.e., the ratio of independent *cis*-eQTL variants [13,550] to the total number of variants tested in the eQTL analysis [5,275,301]), and the prior probability of a variant being associated with the *cis*-CpG ($p2 + p12$) was set to 0.0122 (i.e., the ratio of independent *cis*-meQTL variants [63,976] to the total number of variants tested in the meQTL analysis [5,253,496]). We then tested four different values for p12, corresponding to probabilities of 10%, 25%, 50%, and 75% that an eGene variant was also a lead meQTL variant. Guo et al.[71] propose that best value for *p12* is the value for which the posterior expectation of colocalization is similar to the prior expectation of colocalization. In our analysis, we found that a value of 0.0019 for *p12* (corresponding to a 75% probability that an eQTL was also a meQTL) gave posterior expectations of colocalization that matched prior expectations better than the other three tested values (Figure S6). All downstream analyses were performed with results from the colocalization analyses with $p12 = 0.0019$.

To validate the twin-based whole-skin meQTL eQTL colocalization analysis, we also carried out colocalization analyses using published eQTL summary statistics obtained from 517 suprapubic skin samples from version 8 of the Genotype-Tissue Expression (GTEx) project[72] (accessed on the GTEx portal on January 25, 2024). We followed the colocalization analysis pipeline as outlined above but only performed the analyses with a *p12* value that corresponded to a 75% probability that an eQTL was also a meQTL.

Using 346 samples with gene expression, DNA methylation, and genotype data available, we investigated if potentially colocalized eGene-CpG pairs were under mediation effects using mediation analyses as outlined by Pierce et al.[14] For each potentially colocalized eGene-CpG pair (posterior probability of a common causal variant > 0.8), we tested two hypotheses, first, that DNA methylation was mediating the SNP effect on gene expression (SME mediation), or second, that gene expression was mediating the SNP effect on DNA methylation (SEM mediation). For each hypothesis in each pair, we calculated the "proportion of effect mediated" as $(\beta_{\text{unadjusted}} - \beta_{\text{adjusted}})/\beta_{\text{unadjusted}}$ where, for example, in the SME case, $\beta_{\text{unadjusted}}$ is the SNP effect on gene expression, and β_{adjusted} is the SNP effect after adjusting for DNA methylation. A Sobel *p* value for mediation was then calculated as previously described by Pierce et al.[14]

Results

We explored the genetic basis of the human skin methylome in up to 414 samples from the TwinsUK population cohort. Our 2-fold approach initially estimated the heritability of the skin methylome using a twin-based heritability model and subsequently estimated skin meQTLs characterizing common genetic effects on DNA methylation in human skin. We explored the distribution of heritable effects and meQTLs across different regulatory and functional genomic regions. To investigate the mechanisms by which meQTLs may affect skin phenotypes, we also estimated skin eQTLs and performed colocalization and mediation analyses to identify shared genetic impacts on the skin methylome and transcriptome. Finally, we investigated the relevance of the skin tissue meQTLs to skin phenotypes and disease by integrating our results with published GWAS and EWAS findings and highlight examples of meQTL effects in genes relevant to skin disease.

Heritability of the skin DNA methylome

We initially assessed the relative impact of genetic and environmental effects on skin DNA methylation profiles at over 400,000 CpG sites using whole-skin samples taken from a relatively photo-protected region in 210 older female twins in complete twin pairs (35 MZ twin pairs, 70 DZ twin pairs, mean age: 58 [SD: 8.95]). Fitting twin-based ACE models to methylation levels at each CpG site allowed us to assess the narrow-sense heritability of methylation at each CpG site. As expected, the majority of variability in DNA methylation profiles was attributable to environmental effects not shared between twins (e^2, Figure 1A). However, at more than 15,000 CpG sites (3.7%), over 50% of the observed variation in skin methylation was attributable to narrow-sense heritability (a^2). Our estimate of mean narrow-sense heritability across all measured autosomal CpG sites was 10.02% (SD = 16.36%) in skin.

The mean narrow-sense heritability of skin DNA methylation (10.02%) is substantially lower than previous estimates in blood (20%)[1] also calculated using a twin-based study design, but in a larger sample. Additionally, the proportion of measured CpG sites that were highly heritable ($a^2 > 50\%$) in skin (3.7%), is smaller than that reported in blood (8.9%) for the same DNA methylation array (Illumina 450K). These comparisons suggest that the DNA methylome in skin is overall less heritable than the blood methylome, and consequently, environmental factors have a larger overall effect on the skin methylome.

We observed different patterns of skin DNA methylation heritability for CpGs across regulatory genomic regions (Figure 1B). CpGs located within 200 bp upstream of transcription start sites (TSSs) or within gene exons, which have been found to regulate gene expression,[57] were on average less heritable (mean heritability of 8.5% and 8.6%, respectively) than CpGs located in intergenic regions (mean heritability = 11.7%, Mann-Whitney U Test $p < 2.2 \times 10^{-16}$). Additionally, variable CpG sites (methylation SD > 0.05) tended to be more heritable (Figure 1C) as previously observed in blood.[1]

Local genetic effects on the skin DNA methylome

We next sought to identify common genetic variants that exhibit local effects on the skin methylome in 394 female

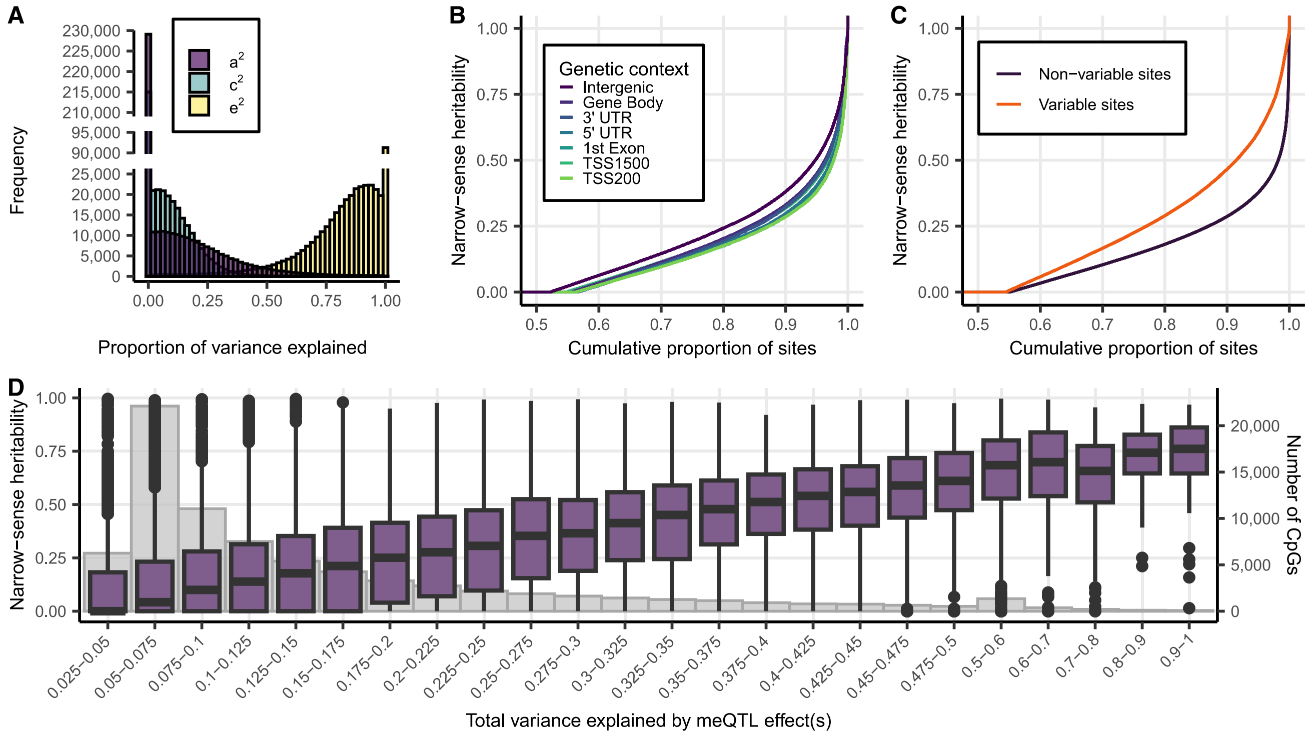

Figure 1. Heritability of the skin DNA methylome

(A) Proportion of DNA methylation variance explained for each tested CpG site by narrow-sense heritability (a^2), common environmental effects between co-twins (c^2), and environmental effects not shared between co-twins (e^2). Y axis is cut-off at 25,000-count.
(B) Cumulative distribution of narrow-sense heritability for CpG sites in different genetic contexts. UTR, untranslated region; TSS1500, 200—1,500 bp upstream of gene transcription start site (TSS); TSS200, <200 bp upstream of gene TSS.
(C) Cumulative distribution of narrow-sense heritability in variable CpG sites (SD > 0.05) vs. non-variable CpG sites.
(D) Combined r^2 of all independent skin meQTL effects per CpG vs. estimated narrow-sense heritability in skin (purple). The number of CpG sites in each binned group is shown in gray.

twins. Using genotypes imputed to the HRC reference panel, we tested the association between ~ 5,200,000 autosomal genetic variants with 407,348 skin CpG methylation levels measured using the Illumina 450K array in whole-skin samples. meQTL identification included adjustment of DNA methylation levels for multiple covariates, in line with the GoDMC pipeline,[2] followed by fitting additive genetic linear models in MatrixEQTL,[52] with a permutation-based approach to calculate FDR adjustment for multiple testing.

At a genome-wide FDR of 5% ($p < 1.56 \times 10^{-5}$), we find >7,500,000 genetic variant-CpG associations in *cis* where the genetic variant was within 1 Mbp to the CpG site. Conditional analysis of these results identified 81,994 independent variant-CpG effects in *cis* (*cis*-meQTL effects). Altogether, 76,442 CpG sites (*cis*-CpGs, 18.8% of tested CpGs) were associated with at least one of 63,976 independent genetic loci (*cis*-meQTLs).

The mean distance between lead independent *cis*-meQTL variants and *cis*-CpGs was 49.8 kb. As expected from studies in other tissues, *cis*-meQTL effects closer to the CpG site had larger effect sizes and smaller *p* values (Figure 2A). The most significant association was observed between cg01543583 and rs1253098, both located in *L3HYPDH* (Figure 2B) 2,137 bp apart.

On average, *cis*-meQTLs explained 14.8% of the variance in *cis*-CpG adjusted DNA methylation levels, ranging from 4.8% to 100%. As expected, *cis*-CpGs had greater average narrow sense heritability (22.1%) compared to non *cis*-CpGs (7.2%), and CpG sites under stronger meQTL effects have higher narrow sense heritability on average (Figure 1D). However, 26.5% (3,996) of highly heritable CpG sites (>50% of variance explained by additive heritable effects) were not associated with an meQTL effect, indicating that a substantial proportion of skin DNA methylation heritability remains unexplained.

Distal genetic effects on the skin DNA methylome

We also identified distal skin meQTL effects, or in *trans*, where the meQTL is at least 1 Mbp away from the CpG site or on a different chromosome. At a genome-wide FDR of 5% ($p < 2.63 \times 10^{-10}$), and after conditional analysis, 1,775 CpG sites were associated with at least one independent genetic variant in *trans*. However, a substantial proportion (27.1%) of these CpGs were also associated with *cis*-meQTLs. This suggests that some *trans* effects may be long-range *cis* effects, that is, driven by LD between a *cis*-meQTL and genetic variants in *trans*. To address this, the conditional analyses were repeated for each CpG site, with *cis* effects included in the analysis if the CpG site had a

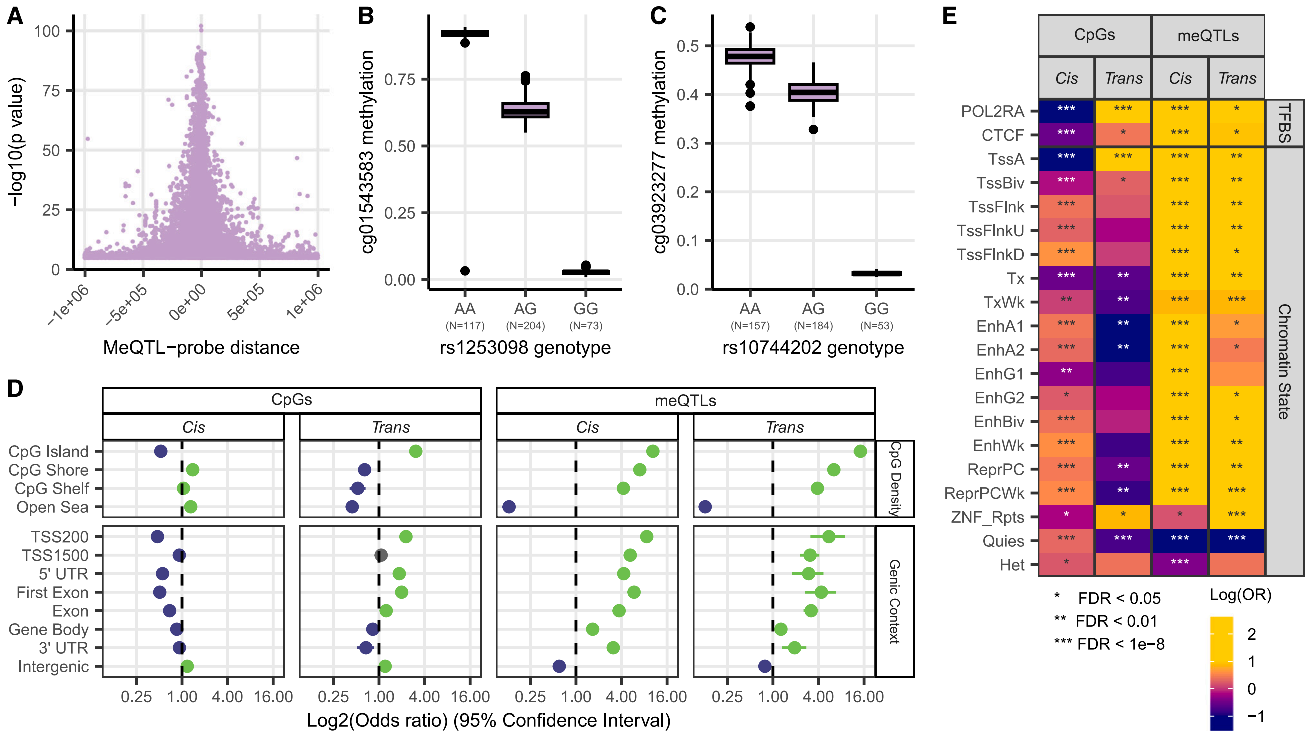

Figure 2. Genetic effects on the whole-skin methylome

(A) Distance between lead *cis*-meQTL and CpG vs. strength of association of the meQTL effect.

(B) Most associated *cis*-meQTL association between variant rs1253098 (chr14:59945536) and cg01543583 (chr14:59947673).

(C) Most associated *trans*-meQTL association between the variant rs10744202 (chr12:125800244) and cg03923277 (chr12:104359732).

(D) Enrichment and depletion of meQTL effects in different genomic contexts. Green indicates significant (FDR < 0.05) enrichment, purple indicates significant depletion. CpG islands based on UCSC refGene annotations (CpG shore, <2 kbp from CpG island; CpG shelf, 2–4 kbp from CpG island; open sea, >4 kbp from CpG island), and gene context is based on refGene annotations (TSS200, < 200 bp from transcription start site (TSS); TSS1500, 200–1,500 bp from TSS).

(E) Enrichment and depletion of meQTL effects in transcription factor binding sites (TFBSs) in suprapubic skin and genomic states in leg skin generated using an 18-state ChromHMM model from the Roadmap Epigenomics Consortium.[59] White asterisks indicate significant depletion, black asterisks indicate significant enrichment. Suprapubic skin TFBS and leg skin chromatin state annotations were obtained from the EpiMap Repository[60] (TssA, active TSS; TssBiv, bivalent/poised TSS; TssFlnk, flanking active TSS; TssFlnkU, flanking TSS upstream; TssFlnkD, flanking TSS downstream; Tx, strong transcription; TxWk, weak transcription; EnhA1, active enhancers 1; EnhA2, active enhancers 2; EnhG1, genic enhancers 1; EnhG2, genic enhancers 2; EnhBiv, bivalent enhancer; EnhWk, weak enhancer; ReprPC, repressed polycomb; ReprPCWk, weak repressed polycomb; ZNF_Rpts, ZNF genes and repeats; Quies, quiscent chromatin; Het, heterochromatin; see Kundaje et al.[59]).

cis-meQTL. This second conditional analysis identified 1,480 *trans*-meQTL effects comprised of 1,438 CpGs (*trans*-CpGs, 0.35% of tested sites) associated with 1,261 independent *trans*-meQTLs. The most significant association was between rs10744202 in *TMEM132B*, and cg03923277 in *TDG* (Figure 2C). Notably, *TDG* has previously been proposed as a therapeutic target to treat melanoma.[73]

Most distal effects were inter-chromosomal, with only a small proportion of intra-chromosomal *trans*-meQTL effects (10.5%). On average, *trans*-meQTL effects had larger effect sizes than *cis*-meQTL effects, which is likely because of increased power to detect *cis*-meQTL effects. As expected, given these larger effect sizes, *trans*-CpGs had higher average heritability (30.8%) compared to *cis*-meQTL effects (22.1%).

Distribution of *cis* and *trans* meQTL-CpG associations in regulatory and functional genomic features

We next examined the distribution of skin meQTLs and associated CpGs across regulatory and functional genomic regions. Both *cis*- and *trans*-meQTLs were significantly depleted (FDR < 0.05) in intergenic and "open sea" (> 4 kbp from a CpG island) regions. Both categories of meQTLs were also enriched in regions relevant to the regulation of gene expression, in particular CpG islands, regions <1,500 bp upstream of TSSs, and in gene exons (Figure 2D).

In contrast, skin *cis*-CpGs showed different genomic annotation patterns to *trans*-CpGs. *Cis*-CpGs were enriched in intergenic and open sea regions and were depleted in CpG islands and all genic regions, mirroring our finding that genic CpGs are less heritable. The distribution of *trans*-CpGs in regulatory regions was more complex. We observed enrichment of *trans*-CpGs in CpG islands, regions <200 bp upstream of TSSs, 5′ UTRs, and intergenic regions, but depletion in 3′ UTRs, gene bodies, and open sea regions (Figure 2D).

We next tested the enrichment of meQTLs and CpGs in functional genomic categories, using leg skin chromatin

state annotations from EpiMap,[60] predicted using an 18-state ChromHMM model. We found that *cis*-meQTLs were depleted in quiescent chromatin and heterochromatin regions and enriched in all other chromatin states. *Trans*-meQTLs were similarly depleted in quiescent chromatin regions but were neither enriched nor depleted within heterochromatin regions (Figure 2E). These distributions broadly follow the distributions of meQTLs seen in regulatory genomic regions.

We again observed contrasting patterns of enrichment for *cis*- vs. *trans*-CpGs across functional genomic categories (Figure 2E). In line with observations from regulatory regions, *cis*-CpGs were depleted in regions of active (i.e., TSSs of transcribed genes) and strong transcription (i.e., in gene bodies of transcribed genes) and enriched in quiescent chromatin and heterochromatin regions (Figure 2E). Additionally, skin *cis*-CpGs were enriched in most types of enhancers, mirroring findings from Min et al.[2] in blood but not in "genic enhancer 2" regions. This enhancer is distinct from others as it is preferentially located within transcribed gene bodies and evolutionarily conserved elements.[59,60] Interestingly, *cis*-CpGs were also enriched in regions with repressing polycomb complexes, which bind to CpG islands in a methylation-dependent manner.[74]

In contrast, the distribution of *trans*-CpGs showed depletion in strongly transcribed regions (i.e., gene bodies of transcribed genes), but enrichment in regions of active transcription (i.e., TSSs of transcribed genes). *Trans*-CpGs were also depleted in active enhancers, quiescent chromatin, and regions with repressing polycomb complexes, again in contrast to *cis*-CpGs.

The differences in the regulatory and functional genomic distribution of *cis*- and *trans*-CpGs suggests that *cis*-meQTL effects might serve a different biological function to *trans*-meQTL effects in skin, in line with findings from previous meQTL studies in non-skin tissues.[2]

Tissue and cell specificity of whole-skin meQTL effects

Previous meQTL studies have shown that a significant proportion of meQTL effects appear to be tissue specific. To explore the tissue specificity of skin meQTLs, we compared our results to meQTLs in blood from Min et al.[2] Altogether, summary statistics for 53,264 independent *cis*- and 1,246 *trans*-meQTL effects reported in whole skin in the current study were also reported in blood by Min et al.,[2] but Min et al.[2] only report meQTL effects that surpassed a nominal significance threshold. Using the π_1 statistic, 59% of previously reported blood *cis*-meQTL effects that we also tested in whole skin (>42 million associations in total, 64.3% of all reported blood CpG-SNP associations in *cis*) validated in whole skin. In total, 1,230 (98.7%) of the whole-skin *trans*-meQTL effects that were reported in blood by Min et al.[2] were validated in blood (at a Bonferroni-adjusted threshold of $p < 4.01 \times 10^{-5}$). Additionally, we observed a high correlation between these reported skin and blood CpG-SNP associations in *cis* ($r_b = 0.825$) (Figure S3) and an even stronger correlation for *trans* effects ($r_b = 0.964$). These findings indicate that most identified skin meQTL effects are not skin specific. However, because Min et al.[2] only report CpG-SNP associations that were nominally significant in the first phase of their study, we expect a bias toward increased validation rates. Despite this, the observed differences in correlation of whole-skin meQTLs with blood meQTLs for *cis* vs. *trans* effects corroborates previous findings from Min et al.[2] that *trans*-meQTL effects are more likely to be found in other tissues compared to *cis* effects.

To understand how whole-skin meQTL effects compare to meQTL effects detected in a single skin cell type, we compared the whole-skin meQTLs with previously reported melanocyte meQTL-CpG associations from Zhang et al.[4] Using the π_1 statistic, we estimated the validation rate of whole-skin *cis*-meQTLs in melanocytes to be only 43.3%. The validation rate of distal signals was also low, with only 64 whole-skin *trans*-meQTL effects validating in melanocytes. However, it should be noted that the estimates of skin cell-type composition in whole-skin sample were very low for melanocytes. Furthermore, the population was different, and sample size of the melanocyte meQTL study ($n = 106$) is lower than of the current whole-skin meQTL study ($n = 394$).

Local genetic effects on the skin transcriptome

To explore the functional impact of meQTLs, we also characterized local eQTL effects in a partially overlapping set of 664 whole-skin samples from female twins, using previously published RNA sequencing data.[37,43] To this end, we tested the association between ~5,200,000 autosomal SNPs with 23,838 RNA sequencing gene expression levels, taking into account biological and technical covariates (see material and methods).

At a genome-wide FDR of 5%, we identified >1.5 million variant-gene associations in *cis* where the genetic variant was within 1 Mbp from the gene TSS. Conditional analysis of these results identified 14,293 independent *cis*-eQTL effects. In total, the expression of 10,255 genes (*cis*-eGenes, 41.5% of tested genes) were associated with at least one of 13,550 independent genetic loci (*cis*-eQTLs).

The median distance between independent *cis*-eQTLs and *cis*-eGene TSSs was 26.3 kbp, and eQTLs closer to the eGene TSS tended to have larger effect sizes and stronger evidence for association (Figure S5). A previous eQTL analysis in a subset ($n = 370$) of this data with a different RNA-seq quantification pipeline, published in the eQTL catalog[75] identified 5,285 *cis*-eGenes at an FDR of 5% (including only genes also tested in our analysis), of which 4,865 (92.05%) were also *cis*-eGenes in our analysis.

Shared genetic effects on the skin methylome and transcriptome

We explored evidence for shared genetic effects on skin gene expression and DNA methylation using colocalization and mediation analyses. We paired *cis*-eGenes with *cis*-CpGs if the lead *cis*-eQTL variant associated with the eGene was significantly associated with the CpG in *cis*.

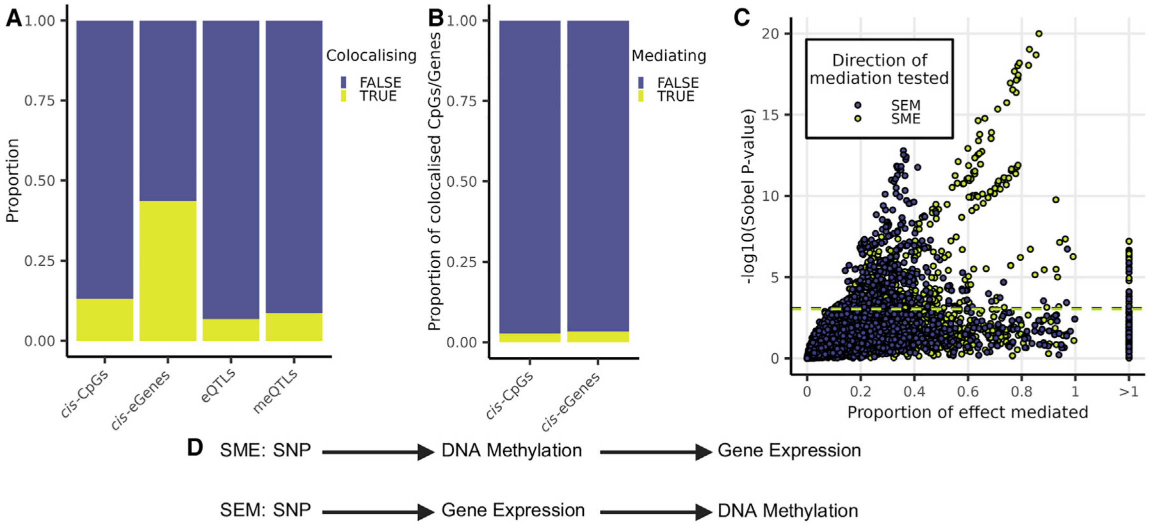

Figure 3. Colocalization and mediation between *cis*-CpGs and *cis*-eGenes

(A) The proportion of *cis*-CpGs and *cis*-eGenes associated with at least one colocalizing QTL and the proportion of *cis*-meQTLs and *cis*-eQTLs that colocalize.

(B) The proportion of *cis*-CpGs and *cis*-eGenes with at least one colocalized QTL that also show evidence of mediation via SEM or SME.

(C) Proportion of effect mediated versus significance of the mediation effect (-log10(Sobel test *p* value). Only results with a positive proportion of the effect mediated are shown. Mediation analyses under the SME model tended to have a larger proportion of effect mediated than results from the SEM model. Purple and yellow dashed lines indicate the FDR 5% threshold for SEM and SME Sobel mediation tests, respectively.

(D) SME and SEM pathways.

Using 83,530 eGene-CpG pairs (including 8,200 eGenes and 25,037 *cis*-CpGs), we performed Bayesian tests of colocalization using the SuSiE method, which relaxes the assumption that there is only a single shared causal variant.[69,70,76] Using a prior probability of a common causal variant ($p12 = 1.93 \times 10^{-3}$), we identified 13,462 potentially colocalized eGene-CpG pairs (posterior probability for a shared casual variant > 0.8), where a single genetic locus is likely acting as both a *cis*-eQTL and a *cis*-meQTL on the paired eGene and CpG, respectively. These colocalized pairs consisted of 4,475 eGenes, and 10,067 *cis*-CpGs, with 3,695 underlying genetic variants (Figure 3A). Altogether, a substantial proportion of genes (44%) and CpGs (13.2%) that are under genetic influence, showed evidence for a shared genetic basis.

To validate the twin-based whole-skin colocalization findings, we also carried out colocalization analyses of the whole-skin meQTLs, with *cis*-eQTLs generated from 517 suprapubic skin samples in version 8 of the GTEx project.[72] The colocalization procedure was the same, but with a $p12$ value of 1.97×10^{-3}. Overall, we observed a similar proportion of eGenes being under the influence of an eQTL effect that is colocalized with an meQTL effect (5,448, 35.2%) in this new suprapubic skin analysis (Figure S16). Furthermore, if we consider only eGenes identified in both the twin-based whole-skin eQTL analysis and in the GTEx suprapubic skin eQTL analysis ($n = 5{,}973$), then we find that 55% (3,321) and 52.8% (3,155) of genes are under an eQTL effect that is also a meQTL effect, respectively (posterior probability for a shared casual variant > 0.8). Altogether, 17.9% (1,070 genes) of eGenes are under a colocalized eQTL effect in both the twin-based and GTEx datasets (Figure S17).

Next, we performed mediation analyses of colocalized pairs, by following the approach of Pierce et al.[14] For each eGene-CpG pair, we tested two hypotheses; first, the SNP effect on methylation mediates expression (SME), and second, the SNP effect on expression mediates methylation (SEM) (Figure 3D). After accounting for co-methylated CpGs (see material and methods), there were 142 SNP-CpG-gene triplets with evidence for eQTL effects mediated via SME, and these included 133 genes. Similarly, 129 SNP-CpG-gene triplets showed evidence for mediation via SEM, including 114 genes. There were 55 SNP-CpG-gene triplets that showed evidence for mediation via both SME and SEM effects. Pierce et al.[14] suggests that when there is evidence for mediation under both the SEM and SME models, the proportion of effect mediated will be higher when the correct model is specified. Under this assumption, 32 of these 55 triplets showed greater evidence for the SME model. Therefore, in total we have evidence for 119 SNP-CpG-gene triplets (in 114 genes) mediated via SME and 97 SNP-CpG-gene triplets (in 87 genes) mediated via SEM (Figures 3B and 3C).

We characterized the distributions of colocalizing and mediating meQTL effects across regulatory regions compared to all *cis*-meQTL effects using genomic annotations as previously described. As expected, *cis*-CpGs under meQTL effects that colocalized with eQTL effects, and the SNPs underlying these colocalized effects, were enriched in regulatory and genic regions compared to all *cis*-CpGs. Additionally, CpGs in mediating SNP-CpG-gene triplets

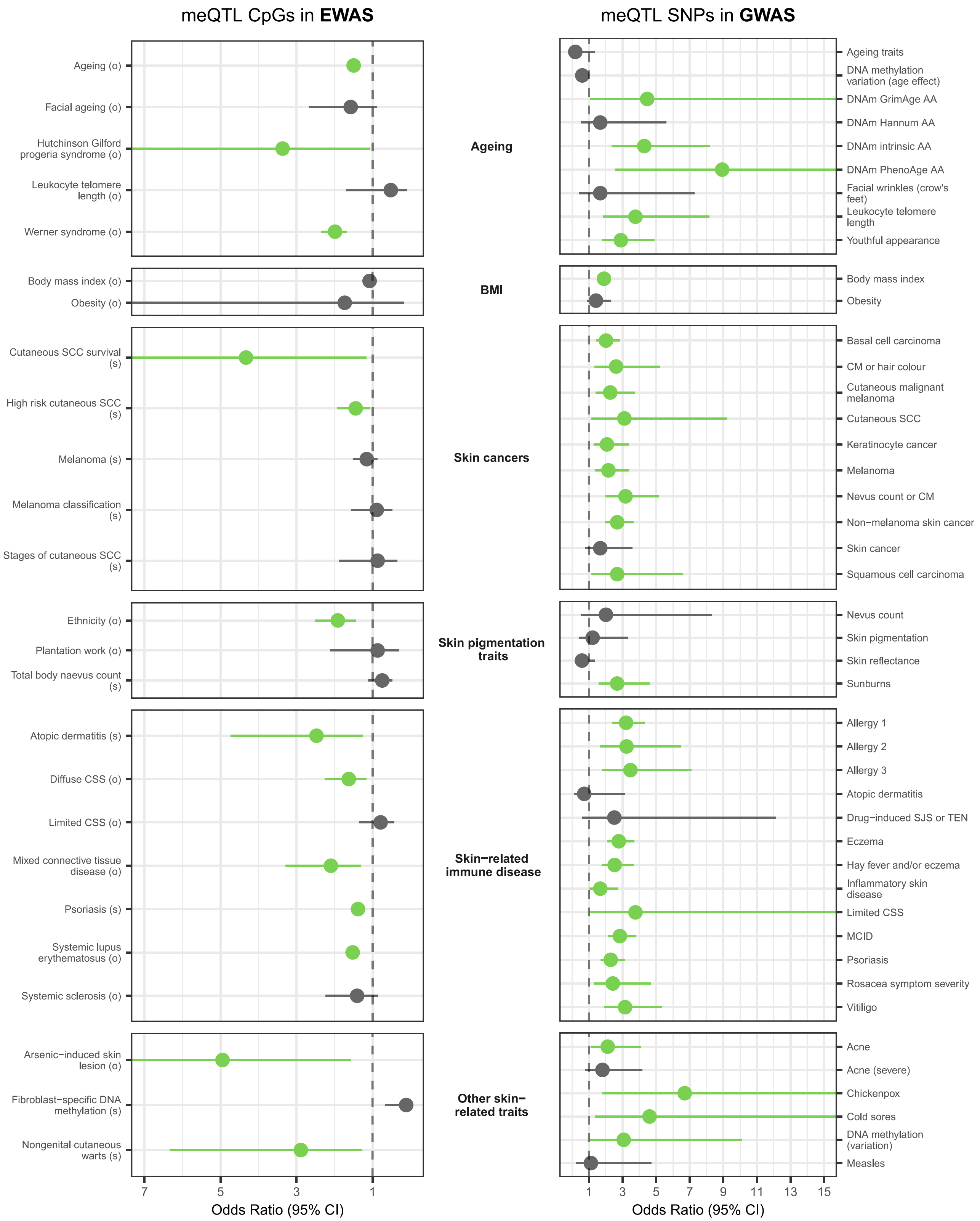

Figure 4. Enrichment of skin meQTL-CpG effects in loci from published epi(genome)-wide association studies of skin-related traits
Left panel shows enrichment of *cis*-CpGs (CpG sites associated with a meQTL in *cis*) to overlap signals from skin-phenotype EWASs. Right panel shows enrichment of *cis*-meQTLs to be in GWAS signals for skin-related phenotypes. Green indicates significant (FDR < 0.05) enrichment. GWAS enrichment analyses are performed using all SNPs associated with a CpG site in *cis*. Studies of the same phenotype have been pooled together. Biological sample types for EWASs is denoted in the EWAS trait label (s, skin; o, other). SCC, squamous cell carcinoma; CSS, cutanous systemic sclerosis; PhenoAge AA, PhenoAge age acceleration; DNAm intrinsic AA, DNAm intrinisic age

(legend continued on next page)

were significantly enriched in gene bodies (odds ratio [OR] = 1.75, $p = 2.9 \times 10^{-5}$) and CpG islands (OR = 1.5, $p = 5 \times 10^{-4}$) for both SME and SEM models. SNPs underling these mediated/mediating meQTL effects were also enriched in regions of gene regulation, including regions within 200 bp of gene TSSs (OR = 9.8, $p = 9.5 \times 10^{-9}$), POL2RA binding sites in leg skin (OR = 9.1, $p = 4.5 \times 10^{-6}$), and CpG islands (OR = 5.9, $p = 5.2 \times 10^{-10}$).

Relevance of skin meQTL effects to skin traits and disease

To explore the relevance of skin meQTL effects to skin diseases and phenotypes, we compared our results to association statistics from the EWAS catalog[61] (Table S2), EWAS atlas[62,63] (Table S3), and GWAS catalog[64] (Table S4).

Altogether, *cis*- or *trans*-CpGs (CpG sites associated with a meQTL in *cis* or *trans*, respectively) were tested for enrichment to be in EWASs of 25 skin-related traits (Tables S2 and S3). *Cis*-CpGs were significantly (FDR < 0.05) enriched to also be EWAS signals for 13 of these skin-related traits (Figure 4).

The trait for which *cis*-CpGs were most significantly enriched for was aging with 63,794 (83.5%) skin *cis*-CpGs compared to 255,187 (77.1%) background CpGs (OR = 1.5, $p < 2.2 \times 10^{-16}$), though aging has the largest number of EWAS signals reported, thus increasing power to detect enrichment. In line with this result, we also observe a significant enrichment of EWAS signals for Werner syndrome and Hutchinson-Gilford progeria syndrome in *cis*-CpGs, both characterized by accelerated aging.[77] These results provide robust evidence that meQTL effects may be related to age-related changes in the skin DNA methylome. Additionally, *cis*-CpGs were enriched for EWAS signals for multiple skin-related immune disease traits. These included diseases with moderate to high heritability such as systemic lupus erythematosus, psoriasis, and atopic dermatitis[78–80] (Figure 4).

At the meQTL level, we compared variants associated with DNA methylation in *cis* or *trans* (i.e., located in *cis*- or *trans*-meQTLs) to GWAS loci for 44 skin-related phenotypes. GWAS signals for 31 of the 44 traits were enriched to be *cis*-meQTLs (FDR < 0.05, with at least five GWAS signals that are also *cis*-meQTL variants) (Figure 4) while none were enriched in *trans*-meQTLs.

Notably, nine of ten tested skin cancer traits were enriched in meQTL effects, including melanoma, substantiating previous findings that meQTL effects play a role in melanoma risk.[4,24] Our results also suggest that meQTLs may play a role in squamous cell carcinoma and keratinocyte cancer risk. Additionally, the meQTL GWAS enrichment results were also in line with *cis*-CpG EWAS enrichment results for aging and skin immune related traits. For example, meQTL enrichment was observed at GWAS loci for 11 of 13 tested skin-related immune traits, including psoriasis, as seen for *cis*-CpGs, as well as eczema and several allergy phenotype clusters (Figure 4).

We also explored the relevance to skin phenotypes of meQTL effects showing evidence of mediation with eQTL effects. Altogether, 280 *cis*-CpGs were previously identified in the mediation set of SNP-CpG-gene triplets. Aging EWAS signals were enriched in these mediation *cis*-CpGs relative to all tested CpGs (OR = 1.76, $p = 6.2 \times 10^{-4}$) but not relative to *cis*-CpGs only. No other EWAS phenotypes were significantly enriched or depleted in these mediating *cis*-CpGs regardless of the selection of background CpGs. SNPs in SNP-CpG-gene mediating triplets were enriched for GWAS signals for six traits, including cutaneous lupus erythematosus, rosacea symptom severity, melanoma, cold sores, vitiligo, and non-melanoma skin cancers, relative to all *cis*-meQTL SNPs. This suggests that mediation via SEM and SME pathways could play a role in genetic susceptibility for a variety of skin phenotypes.

Examples of genetic methylation and expression mediating effects relevant to skin disease

We next focused on specific examples where colocalized genetic, methylation, and expression signals with evidence for mediation effects (SME or SEM) involved previously identified genomic regions and gene transcripts relevant to skin disease. We observed a number of GWAS loci and gene transcripts relevant to skin disease in mediating SNP-CpG-gene triplets. One example is *ALOX12*, which encodes a lipoxygenase enzyme that plays an important role in arachidonic acid metabolism and has been associated with multiple skin diseases. *ALOX12* expression is a biomarker of melanoma and malignancy[81] and a potential therapeutic target for vitiligo.[82] Additionally, the excretion of its metabolite 12(S)-hydroxyeicosatetraenoic acid in urine is significantly increased in individuals with psoriasis,[83] and most recently, an increase in *Alox12* expression has been observed in mouse models of atopic dermatitis.[84] Furthermore, an increase in expression of *Alox12* induced by the transcription factor p63 binding to the *Alox12* promoter is known to play an important role in the formation of the epidermal barrier during development,[85] highlighting its strong association with skin physiology.

In our data, *ALOX12* expression is under the influence of an eQTL within the first ~6 kbp of the gene. This eQTL SNP also acts as a *cis*-meQTL for cg05215272 (posterior probability of colocalisation = 0.95), a CpG site located in a CpG island 289 bp upstream of the *ALOX12* TSS. Mediation analysis indicates that this meQTL mediates the eQTL association (Sobel $p = 0.0007$), explaining approximately

acceleration; GrimAge AA, GrimAge age acceleration; DNAm Hannum, Hannum DNAm age acceleration; CM, cutaneous melanoma; MCID; multiple chronic inflammatory disease; SJS, Stevens-Johnson syndrome; TEN, toxic epidermal necrolysis; Allergy 1, asthma, hay fever, or eczema; Allergy 2, age of onset of asthma, hay fever, and/or eczema; Allergy 3, multivariate analysis of asthma, hay fever, and/or eczema.

73% of the eQTL effect. Interestingly, after adjusting for covariates, methylation at cg05215272 and expression of *ALOX12* are positively correlated (r = 0.3, after adjusting for covariates), an effect previously observed at *ALOX12* in atherosclerotic plaques by Kim et al.[86] Traditionally, DNA methylation near the TSS of a gene is associated with down regulation of gene expression, but the opposite effect has also been documented in multiple studies.[87,88] Our results indicate that the *cis*-meQTL effect is a key regulator of *ALOX12* expression in skin (Figure 5A).

Another example of a skin-disease-relevant SNP-CpG-gene SME mediation involves *CSPG4*, or melanoma-associated chondroitin sulfate proteoglycan,. *CSPG4* is selectively up regulated in melanoma, is essential to melanoma growth, and has been identified as a target for chimeric antigen receptor T cell therapy for individuals with melanoma.[89,90] We find that an eQTL effect of rs4886456 on *CSPG4* is mediated by DNA methylation at the rs4886456 associated cg21460582 (Sobel $p = 0.0001$). rs4886456 is both an eQTL for *CSPG4* and an meQTL for cg21460582 (posterior probability of colocalization = 0.95) and is located in an intergenic region 10.6 kbp upstream of the TSS of *CSPG4* and 2 kbp upstream of *ODF3L1*. Interestingly, rs4886456 is located within a region predicted to have higher levels of H3K27AC in suprapubic skin,[91,92] which is a marker of increased transcriptional activity. cg21460582 is located 672 bp upstream of the *CSPG4* TSS. Unlike the *ALOX12* example, here cg21460582 methylation and *CSPG4* expression are negatively correlated (r = −0.39), indicating a putative role of cg21460582 in down regulation of *CSPG4* expression (Figure 5B). Regulation of *CSPG4* by this pathway could be relevant to the up regulation of the gene in melanoma cells.

Another example of a mediation effect related to skin disease involves *CDK10* with rs1805008 (c.478C> T [GenBank: NM_002386.4] [p.Arg160Trp]), a missense variant in an exon of *MC1R*. *MC1R* variants, and rs1805008 in particular, have been identified as important risk variants for melanoma, keratinocyte carcinoma, and sunburn.[29,93,94] *CDK10* is an important gene in several cancers, although its specific functional effect (i.e., tumor suppression or up regulation) is unclear.[95]

We find that the rs1805008 risk allele for melanoma, keratinocyte cancer, and sunburn (C > T) is associated with reduced expression of *CDK10* and also reduced DNA methylation of cg00001687 (posterior probability of colocalization = 0.96) located within one of the last introns of *MC1R*. Mediation analysis shows putative mediation via both SME or SEM pathways (Sobel $p = 0.00017$ and 0.00011, respectively), although SEM is more likely (26% mediation in the SEM model vs. 18% mediation in the SME model). The location of cg00001687 in the gene body of *CDK10* provides a possible mechanism to explain SEM mediation (Figure 5C). Baubec et al.[96] showed that DNMT3B binds to the bodies of transcribed genes, promoting DNA methylation of CpG sites in gene bodies, which could explain our observation that decreased expression of *CDK10* results in decreased methylation of cg0000 1687. A previous study by Bonilla et al.[97] used summary-based Mendelian randomization to show that *CDK10* expression is reduced in self-diagnosed melanoma and in sun-exposed skin (compared to non-sun-exposed skin) via a pleiotropic effect with rs1805008, reinforcing our findings. Despite this, further work is needed to determine if DNA methylation at cg00001687 is involved in the association between *CDK10* expression and melanoma and the precise mechanisms underlying this link.

Discussion

In this study, we report genome-wide genetic analysis of DNA methylation profiles in human whole skin, identifying skin meQTL effects in multiple GWAS loci for skin conditions and diseases. As expected, most meQTL effects were local, rather than distal, which likely in part reflects differences in statistical power. We observed lower methylation heritability in skin compared to other tissues, suggesting a stronger impact of environmental factors on the skin methylome relative to previously studied tissues and cell types. We find robust evidence for coordinated genetic effects on the skin methylome and transcriptome and identify over two hundred putative mediation effects that underlie this shared genetic basis. The results give insights into the genetic regulation of skin DNA methylation, the regulation of skin gene expression, and human skin disease pathways.

The twin-based, narrow-sense heritability of the skin methylome is lower than previously reported in blood[1] and adipose tissue.[9] The results indicate that the skin methylome is more susceptible to environmental influences, and by its nature, skin is a barrier exposed to a number of physical and chemical environmental factors. We find that a smaller proportion of Illumina 450K CpG sites are under meQTL effects in skin compared to blood where Min et al.[2] identified over twice as many *cis*- or *trans*-CpGs. However, there are significant differences in power to detect meQTLs between the two studies. Regardless, we find that skin and blood meQTL effects reported in both datasets correlate strongly, with an r_b of 0.825 for *cis*-meQTL effects, and 0.964 for *trans*-meQTL effects. Our results suggest that the majority of whole-skin meQTLs identified in the current study are not predominantly skin specific. However, because Min et al.[2] only report SNP-CpG associations that reached nominal significance in blood, we expect that our cross-tissue comparison will be biased toward increased tissue-shared rates. It is unclear if whole-skin meQTL effects that are not reported by Min et al.[2] were never tested or were tested but were not nominally significant.

Previous work has identified genetic effects on DNA methylation levels in human melanocytes.[4] We estimated a replication rate of 43.3% of whole-skin local meQTL

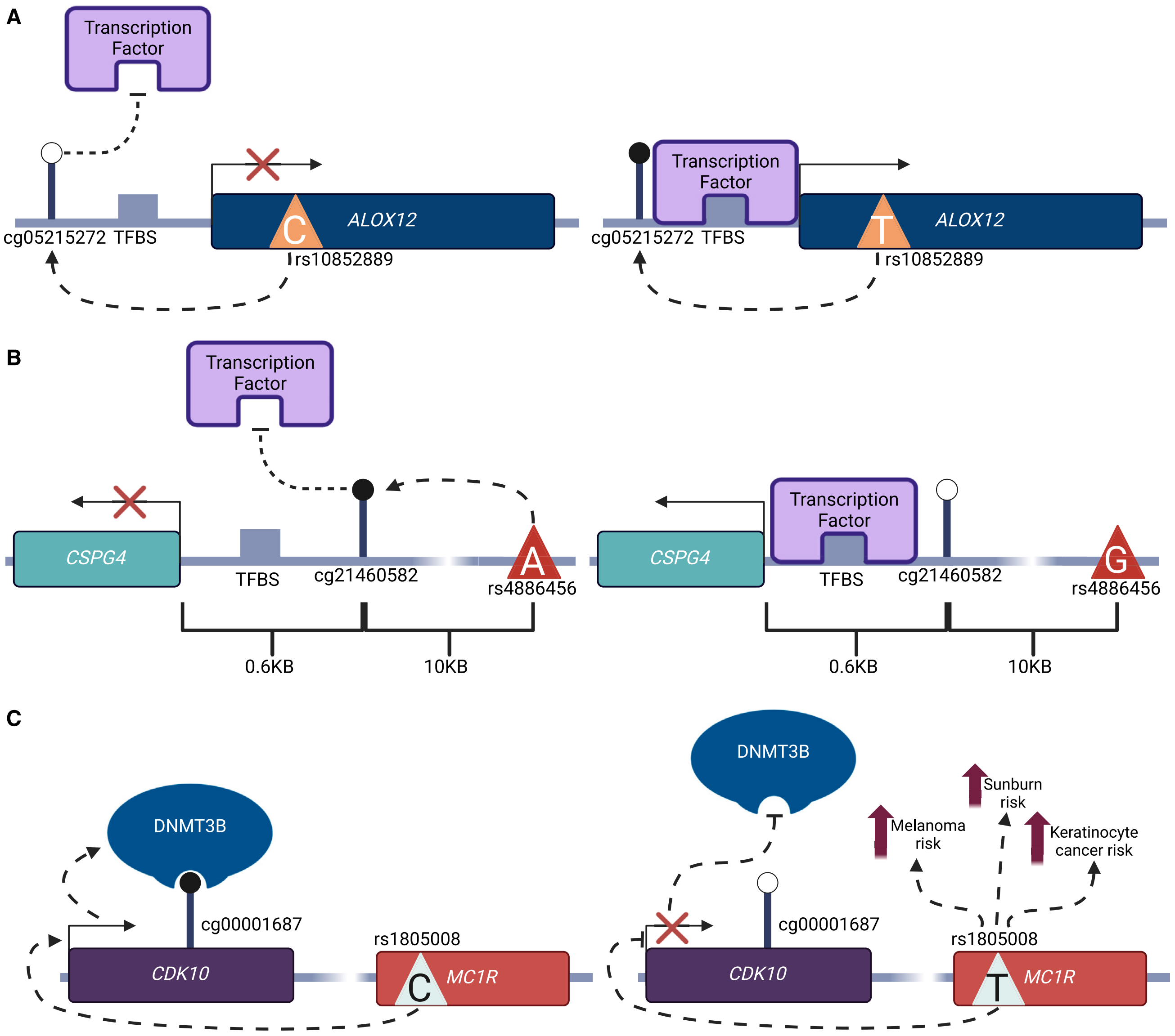

Figure 5. Three examples of mediating SNP-CpG-gene triplets
Methylated CpG sites are represented by a filled black circle, and un-methylated CpG sites are represented by an empty circle.
(A) Hypothetical mechanism by which cg05215272 mediates an eQTL effect on *ALOX12* expression where *ALOX12* has been implicated in melanoma, vitiligo, psoriasis, and atopic dermatitis.
(B) Hypothetical mechanism by which cg21460582 mediates an eQTL effect on *CSPG4* expression, which is strongly associated in melanoma.
(C) Hypothetical mechanism by which *CDK10* expression mediates a meQTL effect on cg00001687.

effects in melanocytes. This replication rate is likely affected by several differences between the two studies. Zhang et al.[4] used cultured melanocytes from newborn males while our study used whole-skin samples that were not cultured and originated from older females. These study differences likely contribute toward the low meQTL replication rate.

Our genomic and functional annotation analyses show distinct patterns for CpGs under genetic control of local and distal meQTL effects. This suggests that *cis*- and *trans*-meQTL effects likely operate through distinct molecular mechanisms, as previously discussed in blood.[2,3,12,19] It has previously been suggested that *trans*-CpGs may operate through local eQTL effects on genes that produce DNA-binding proteins, which might then bind to DNA at or near to distal *trans*-CpGs, resulting in methylation changes in these distal genetic regions.[2,3,98] In line with this hypothesis, skin *trans*-CpGs are strongly enriched to fall in regions of gene regulation and transcription factor binding. On the other hand, skin *cis*-CpGs were depleted in these regulatory and transcription factor binding regions, suggesting that they operate under a different mechanism. One such mechanism previously discussed is that *cis*-meQTLs may affect local chromatin organization,

changing the activity of DNMT and TET proteins, thus impacting DNA methylation at nearby CpG sites.[98] Therefore, our genomic annotation results in skin further reinforce similar findings in blood and other tissues.[2,3,12]

Previous studies have reported that a substantial proportion of meQTLs exhibit coordinated effects on gene expression.[14,16] To explore functional consequences of whole-skin meQTLs, we carried out meQTL-eQTL colocalization analyses, observing evidence for shared genetic effects affecting over 10,000 CpGs (13.2% of *cis*-CpGs) and 4,000 genes (43.7% of *cis*-eGenes) in skin. Of these, we identified mediation effects in 217 pairs, showing that at least 114 genes have eQTL effects mediated at least partially by an meQTL effect, and 87 genes have eQTL effects mediating local meQTL effects. Banovich et al.[16] estimated that perhaps 25% of eQTL effects identified in a lymphoblastoid cell line (LCL) were also in meQTLs, while only 7% of skin eQTLs were colocalized with an meQTL in our analysis. Partly this is attributable to the small number of eQTLs (595) identified and thus tested for meQTL effects by Banovich et al.,[16] but differences between our study and that of Banovich et al.,[16] such as the use of immortalized LCL cultures, are also likely to contribute. Pierce et al.[14] reported 2,913 potentially colocalized eQTL-meQTL pairs in blood while we identified 13,462 potentially colocalized eQTL-meQTL pairs in skin at the same threshold (posterior probability for a shared casual variant > 0.8), using similar sample sizes (Pierce et al.[14] eQTL sample size $= 992$, meQTL sample size $= 337$) and numbers of identified *cis*-CpGs (Pierce et al.[14] identified 77,664 blood *cis*-CpGs, we identified 76,442 skin *cis*-CpGs). One explanation for this is our use of coloc-SuSIE, which, in contrast to the method used by Pierce et al.,[14] relaxes the assumption of a single causal variant for each phenotype. Results from our conditional analysis of skin eQTLs indicate that this assumption is often invalid, and as such using coloc-SuSIE gives us greater power to detect colocalized eQTL-meQTL pairs. In addition, colocalization analyses using our whole-skin meQTL data and suprapubic skin *cis*-eQTL data from the GTEx project,[72] validated our whole-skin eQTL-meQTL colocalization analyses. In line with results from both Pierce et al.[14] and Gutierrez-Arcelus et al.,[17] we find that eQTL-meQTL mediation effects can occur under both SME and SEM pathways. While we detect more mediated SNP-CpG-gene pairs via the SME pathway than via the SEM pathway, this highlights the complexity of effects linking the genome, epigenome, and transcriptome.

We observed an enrichment of whole-skin meQTL effects in loci previously associated with multiple skin diseases and traits in genetic and epigenetic studies. We find that *cis*-CpGs are enriched in many published EWASs of skin-related diseases and phenotypes. Of the eight traits with EWASs that were entirely performed in skin (total body nevus count, melanoma classification, melanoma, high-risk cutaneous squamous cell carcinoma (cSCC), stages of cSCC, cSCC survival, non-genital cutaneous warts, and psoriasis), four were enriched for *cis*-CpGs (high-risk cSCC, cSCC survival, non-genital cutaneous warts, and psoriasis). *Cis*-CpGs identified in EWASs of these traits included a CpG in the gene body of *ARRB2* (involved in the Hedgehog signaling pathway, which has been linked to SCC[99]) for high-risk cSCC, a CpG in the 3′ UTR of *CSK* (involved in Src kinase signaling, which has been linked to squamous cell carcinoma [SCC][100]) for cSCC survival, a CpG in the gene body of *RAP1GAP* (which has been previously linked to human papillomavirus infection[101]) for non-genital cutaneous warts, and four CpGs near the TSS of *PSORS1C1* (psoriasis susceptibility 1 candidate 1) for psoriasis. Furthermore, an enrichment of skin meQTL effects was observed in many more skin phenotype GWAS. In particular, skin cancer GWASs, where we observed significant enrichment for nine out of ten cancer phenotypes (basal cell carcinoma, cutaneous melanoma or hair color, cutaneous malignant melanoma, melanoma, nevus count or cutaneous melanoma, cutaneous SCC, SCC, keratinocyte cancer, and non-melanoma skin cancer). These enrichments were attributable to skin cancer GWAS loci such as rs7705526, a variant located in an intron of *TERT*, and rs1805008, a missense variant of *MC1R*. In addition, a similar pervasive enrichment of meQTL effects was observed for GWAS loci of skin-related immune traits and diseases, with 11 of 13 considered phenotypes showing enrichment effects. These were attributed to disease-risk loci including loci in or near *NOS2* (psoriasis), *TLR1* (eczema and allergic disease phenotypes), and *FADS2* (vitiligo). Psoriasis was one of the few phenotypes for which we observed an enrichment of skin *cis*-meQTLs in GWAS loci and an enrichment of skin *cis*-CpGs in EWAS loci. These enrichments are driven at least partly by CpG-meQTL pairs where both the CpG under the meQTL effect and a SNP or SNPs within the meQTL are associated with psoriasis. For example, the intergenic *cis*-CpG cg23904955 has been identified as differentially methylated in psoriatic skin compared to healthy skin from individuals with psoriasis ($n = 24$),[102] and the lead SNP of its associated meQTL, rs10748781, has been identified as a risk marker for multiple immune traits, including psoriasis (6,530 psoriasis cases, 34,213 controls).[103]

We also observed that meQTL effects may mediate eQTL effects in skin, including in genes relevant to skin disease. Two specific examples highlighted here focused on meQTL effects mediating eQTL effects on genes linked to melanoma. The first was in *ALOX12*, a biomarker of melanoma, which has an eQTL in skin that is likely mediated via an meQTL effect on a CpG island near the *ALOX12* TSS. In addition to being a biomarker of melanoma malignancy,[81] *ALOX12* expression has also been linked to vitiligo,[82] psoriasis,[83] and atopic dermatitis.[84] On the other hand, *CSPG4*, which is essential for melanoma growth, is under an eQTL effect mediated by an meQTL effect on a CpG site 672 bp upstream of the *CSPG4* TSS. *CSPG4* is essential to melanoma growth and may even represent a therapeutic target for treatment of melanoma.[89,90] In addition, the findings here are even more notable, given that the meQTL

effects are influencing gene activity within healthy skin, indicating the mediation effect might be conferring susceptibility of skin to these different diseases (rather than the presence thereof).

This study characterizes the genetic basis of whole-skin DNA methylation at a genome-wide level; however, there are a number of limitations. One limitation is that our analyses included individuals of European ancestry, and genetic effects on skin DNA methylation might differ in other genetic ancestry groups. Another limitation is the relatively modest sample size, with 394 skin DNA methylation profiles and 664 skin gene expression profiles used for meQTL and eQTL detection, respectively. Although we detect a large number of *cis*-meQTLs, power to detect *cis* effects with smaller effect sizes, as well as *trans*-meQTL effects, is limited. A related point is that the sample size is limited for undertaking mediation analyses of meQTLs and eQTLs ($n = 346$). The mediation analyses also make two prior assumptions, of no measurement error, and a linear effect of the SNP on both mediator and outcome variable. To address these, we adjusted both gene expression and DNA methylation levels for multiple covariates.

Another limitation relates to the uncertainty in estimating cell-type proportions within whole skin. We used EpiSCORE[41,42] for cell-type prediction and validated these by combining our DNA methylation data with the human skin dermis and epidermis methylome data from Vandiver et al.[25] A principal component analysis (PCA) of the combined data clearly differentiates dermis and epidermis samples, and our whole-skin samples skew toward dermis (Figure S1). The PC loadings strongly correlate with the EpiSCORE-predicted skin-cell-type proportions, in particular those most representative of dermis and epidermis (fibroblasts and keratinocytes, respectively) (Figure S2). Despite this validation, there is still uncertainty, particularly for the melanocyte estimates, which were null, while we expect that a small number of melanocytes to be present. Therefore, we did not include these EpiSCORE cell-type estimates as covariates in the QTL analyses, but instead adjusted for PCs. Nonetheless, the PC adjustment may remove cell-type or dermis-/epidermis-specific meQTL effects that may be important to skin disease, such as the melanocyte-specific effects identified by Zhang et al.[4] Therefore, our approach may tend toward identification of meQTL effects shared across skin cell types, which are more likely to be shared across tissues than cell-specific effects.

Lastly, our study identified that skin *cis*-CpGs are enriched in many signals from published EWASs of skin-related diseases and phenotypes. However, many published EWASs were performed using DNA methylation data from non-skin tissues, predominantly blood (Tables S2 and S3). It is unknown if these skin trait DNA methylation effects from other tissues are also present in skin; however, a large proportion of the identified skin meQTL effects were shared with blood meQTLs. This might partly be due to presence of immune cells in skin and highlights how persistent genetic effects on DNA methylation are in cells across blood and skin.

In summary, our study identified meQTL effects in whole skin. The skin meQTL effects tend to be predominantly tissue shared and replicate previous melanocyte specific effects. A substantial proportion of signals show evidence for a shared genetic basis between skin methylation and expression and are located in genomic regions previously associated with skin-related phenotypes and disease. The results show the utility of identifying these genetic effects on the skin methylome for investigating the regulatory genomics of skin-related diseases and phenotypes.

Data and code availability

The skin methylome dataset in this manuscript is deposited in the EGA: EGAS00001007816. Summary statistics for whole-skin meQTLs are available from https://epicmeqtl.kcl.ac.uk/.

Supplemental information

Supplemental information can be found online at https://doi.org/10.1016/j.ajhg.2024.07.010.

Acknowledgments

We thank the TwinsUK twin research volunteers for their participation in this research study. We thank Dr. Johanna Sandling for help on generating the DNA methylation profiles and original data checks. We acknowledge use of the research computing facility at King's College London, CREATE (https://doi.org/10.18742/rnvf-m076). This project was funded by a UKRI BBSRC iCASE studentship with Unilever (BB/V509656/1). The TwinsUK study is funded by the Wellcome Trust, Medical Research Council, Versus Arthritis, European Union Horizon 2020, Chronic Disease Research Foundation (CDRF), ZOE LIMITED, and the National Institute for Health Research (NIHR) Clinical Research Network (CRN) and Biomedical Research Center based at Guy's and St Thomas' NHS Foundation Trust in partnership with King's College London.

Author contributions

C.J.S. and J.T.B. conceived and designed the study. C.J.S. conducted the analysis. S.V., J.S.E.-S.M., and A.L.R. contributed to the methods and analysis. J.T.B. provided study oversight. J.T.B., K.S.S., T.D.S., P.D., V.B., and D.A.G. contributed data or helped with results interpretation. C.J.S. and J.T.B. wrote the paper. All authors approved the final manuscript prior to submission.

Declaration of interests

DG is an employee of Unilever.

Received: December 5, 2023
Accepted: July 15, 2024
Published: August 12, 2024

References

1. Van Dongen, J., Nivard, M.G., Willemsen, G., Hottenga, J.-J., Helmer, Q., Dolan, C.V., Ehli, E.A., Davies, G.E., van Iterson, M., Breeze, C.E., et al. (2016). Genetic and Environmental Influences Interact with Age and Sex in Shaping the Human Methylome. Nat. Commun. *7*, 11115. https://doi.org/10.1038/ncomms11115.
2. Min, J.L., Hemani, G., Hannon, E., Dekkers, K.F., Castillo-Fernandez, J., Luijk, R., Carnero-Montoro, E., Lawson, D.J., Burrows, K., Suderman, M., et al. (2021). Genomic and Phenotypic Insights from an Atlas of Genetic Effects on DNA Methylation. Nat. Genet. *53*, 1311–1321. https://doi.org/10.1038/s41588-021-00923-x.
3. Huan, T., Joehanes, R., Song, C., Peng, F., Guo, Y., Mendelson, M., Yao, C., Liu, C., Ma, J., Richard, M., et al. (2019). Genome-Wide Identification of DNA Methylation QTLs in Whole Blood Highlights Pathways for Cardiovascular Disease. Nat. Commun. *10*, 4267. https://doi.org/10.1038/s41467-019-12228-z.
4. Zhang, T., Choi, J., Dilshat, R., Einarsdóttir, B.Ó., Kovacs, M.A., Xu, M., Malasky, M., Chowdhury, S., Jones, K., Bishop, D.T., et al. (2021). Cell-Type-Specific meQTLs Extend Melanoma GWAS Annotation beyond eQTLs and Inform Melanocyte Gene-Regulatory Mechanisms. Am. J. Hum. Genet. *108*, 1631–1646. https://doi.org/10.1016/j.ajhg.2021.06.018.
5. Hawe, J.S., Wilson, R., Schmid, K.T., Zhou, L., Lakshmanan, L.N., Lehne, B.C., Kühnel, B., Scott, W.R., Wielscher, M., Yew, Y.W., et al. (2022). Genetic Variation Influencing DNA Methylation Provides Insights into Molecular Mechanisms Regulating Genomic Function. Nat. Genet. *54*, 18–29. https://doi.org/10.1038/s41588-021-00969-x.
6. Shi, J., Marconett, C.N., Duan, J., Hyland, P.L., Li, P., Wang, Z., Wheeler, W., Zhou, B., Campan, M., Lee, D.S., et al. (2014). Characterizing the Genetic Basis of Methylome Diversity in Histologically Normal Human Lung Tissue. Nat. Commun. *5*, 3365. https://doi.org/10.1038/ncomms4365.
7. Schulz, H., Ruppert, A.-K., Herms, S., Wolf, C., Mirza-Schreiber, N., Stegle, O., Czamara, D., Forstner, A.J., Sivalingam, S., Schoch, S., et al. (2017). Genome-Wide Mapping of Genetic Determinants Influencing DNA Methylation and Gene Expression in Human Hippocampus. Nat. Commun. *8*, 1511. https://doi.org/10.1038/s41467-017-01818-4.
8. Volkov, P., Olsson, A.H., Gillberg, L., Jørgensen, S.W., Brøns, C., Eriksson, K.-F., Groop, L., Jansson, P.-A., Nilsson, E., Rönn, T., et al. (2016). A Genome-Wide mQTL Analysis in Human Adipose Tissue Identifies Genetic Variants Associated with DNA Methylation, Gene Expression and Metabolic Traits. PLoS One *11*, e0157776. https://doi.org/10.1371/journal.pone.0157776.
9. Grundberg, E., Meduri, E., Sandling, J.K., Hedman, Å.K., Keildson, S., Buil, A., Busche, S., Yuan, W., Nisbet, J., Sekowska, M., et al. (2013). Global Analysis of DNA Methylation Variation in Adipose Tissue from Twins Reveals Links to Disease-Associated Variants in Distal Regulatory Elements. Am. J. Hum. Genet. *93*, 876–890. https://doi.org/10.1016/j.ajhg.2013.10.004.
10. Yang, Y., Wu, L., Shu, X., Lu, Y., Shu, X.-O., Cai, Q., Beeghly-Fadiel, A., Li, B., Ye, F., Berchuck, A., et al. (2019). Genetic Data from Nearly 63,000 Women of European Descent Predicts DNA Methylation Biomarkers and Epithelial Ovarian Cancer Risk. Cancer Res. *79*, 505–517. https://doi.org/10.1158/0008-5472.CAN-18-2726.
11. Dai, J.Y., Wang, X., Wang, B., Sun, W., Jordahl, K.M., Kolb, S., Nyame, Y.A., Wright, J.L., Ostrander, E.A., Feng, Z., and Stanford, J.L. (2020). DNA Methylation and Cis-Regulation of Gene Expression by Prostate Cancer Risk SNPs. PLoS Genet. *16*, e1008667. https://doi.org/10.1371/journal.pgen.1008667.
12. Villicaña, S., Castillo-Fernandez, J., Hannon, E., Christiansen, C., Tsai, P.-C., Maddock, J., Kuh, D., Suderman, M., Power, C., Relton, C., et al. (2023). Genetic Impacts on DNA Methylation Help Elucidate Regulatory Genomic Processes. Genome Biol. *24*, 176. https://doi.org/10.1186/s13059-023-03011-x.
13. Hannon, E., Gorrie-Stone, T.J., Smart, M.C., Burrage, J., Hughes, A., Bao, Y., Kumari, M., Schalkwyk, L.C., and Mill, J. (2018). Leveraging DNA-Methylation Quantitative-Trait Loci to Characterize the Relationship between Methylomic Variation, Gene Expression, and Complex Traits. Am. J. Hum. Genet. *103*, 654–665. https://doi.org/10.1016/j.ajhg.2018.09.007.
14. Pierce, B.L., Tong, L., Argos, M., Demanelis, K., Jasmine, F., Rakibuz-Zaman, M., Sarwar, G., Islam, M.T., Shahriar, H., Islam, T., et al. (2018). Co-Occurring Expression and Methylation QTLs Allow Detection of Common Causal Variants and Shared Biological Mechanisms. Nat. Commun. *9*, 804. https://doi.org/10.1038/s41467-018-03209-9.
15. Shang, L., Zhao, W., Wang, Y.Z., Li, Z., Choi, J.J., Kho, M., Mosley, T.H., Kardia, S.L.R., Smith, J.A., and Zhou, X. (2023). meQTL Mapping in the GENOA Study Reveals Genetic Determinants of DNA Methylation in African Americans. Nat. Commun. *14*, 2711. https://doi.org/10.1038/s41467-023-37961-4.
16. Banovich, N.E., Lan, X., McVicker, G., van de Geijn, B., Degner, J.F., Blischak, J.D., Roux, J., Pritchard, J.K., and Gilad, Y. (2014). Methylation QTLs Are Associated with Coordinated Changes in Transcription Factor Binding, Histone Modifications, and Gene Expression Levels. PLoS Genet. *10*, e1004663. https://doi.org/10.1371/journal.pgen.1004663.
17. Gutierrez-Arcelus, M., Lappalainen, T., Montgomery, S.B., Buil, A., Ongen, H., Yurovsky, A., Bryois, J., Giger, T., Romano, L., Planchon, A., et al. (2013). Passive and Active DNA Methylation and the Interplay with Genetic Variation in Gene Regulation. Elife *2*, e00523. https://doi.org/10.7554/eLife.00523.
18. Xiong, X., Hou, L., Park, Y., Molinie, B., Gregory, R.I., Kellis, M., and Kellis, M. (2021). Genetic Drivers of m6A Methylation in Human Brain, Lung, Heart and Muscle. Nat. Genet. *53*, 1156–1165. https://doi.org/10.1038/s41588-021-00890-3.
19. Smith, A.K., Kilaru, V., Kocak, M., Almli, L.M., Mercer, K.B., Ressler, K.J., Tylavsky, F.A., and Conneely, K.N. (2014). Methylation Quantitative Trait Loci (meQTLs) Are Consistently Detected across Ancestry, Developmental Stage, and Tissue Type. BMC Genom. *15*, 145. https://doi.org/10.1186/1471-2164-15-145.
20. Gibbs, J.R., van der Brug, M.P., Hernandez, D.G., Traynor, B.J., Nalls, M.A., Lai, S.-L., Arepalli, S., Dillman, A., Rafferty, I.P., Troncoso, J., et al. (2010). Abundant Quantitative Trait Loci Exist for DNA Methylation and Gene Expression in Human Brain. PLoS Genet. *6*, e1000952. https://doi.org/10.1371/journal.pgen.1000952.

21. Oliva, M., Demanelis, K., Lu, Y., Chernoff, M., Jasmine, F., Ahsan, H., Kibriya, M.G., Chen, L.S., and Pierce, B.L. (2023). DNA Methylation QTL Mapping across Diverse Human Tissues Provides Molecular Links between Genetic Variation and Complex Traits. Nat. Genet. *55*, 112–122. https://doi.org/10.1038/s41588-022-01248-z.
22. Lin, D., Chen, J., Perrone-Bizzozero, N., Bustillo, J.R., Du, Y., Calhoun, V.D., and Liu, J. (2018). Characterization of Cross-Tissue Genetic-Epigenetic Effects and Their Patterns in Schizophrenia. Genome Med. *10*, 13. https://doi.org/10.1186/s13073-018-0519-4.
23. De Araújo, É.S.S., Pramio, D.T., Kashiwabara, A.Y., Pennacchi, P.C., Maria-Engler, S.S., Achatz, M.I., Campos, A.H.J.F.M., Duprat, J.P., Rosenberg, C., Carraro, D.M., and Krepischi, A.C.V. (2015). DNA Methylation Levels of Melanoma Risk Genes Are Associated with Clinical Characteristics of Melanoma Patients. BioMed Res. Int. *2015*, 376423. https://doi.org/10.1155/2015/376423.
24. Roos, L., Sandling, J.K., Bell, C.G., Glass, D., Mangino, M., Spector, T.D., Deloukas, P., Bataille, V., and Bell, J.T. (2017). Higher Nevus Count Exhibits a Distinct DNA Methylation Signature in Healthy Human Skin: Implications for Melanoma. J. Invest. Dermatol. *137*, 910–920. https://doi.org/10.1016/j.jid.2016.11.029.
25. Vandiver, A.R., Irizarry, R.A., Hansen, K.D., Garza, L.A., Runarsson, A., Li, X., Chien, A.L., Wang, T.S., Leung, S.G., Kang, S., and Feinberg, A.P. (2015). Age and Sun Exposure-Related Widespread Genomic Blocks of Hypomethylation in Nonmalignant Skin. Genome Biol. *16*, 80. https://doi.org/10.1186/s13059-015-0644-y.
26. Bormann, F., Rodríguez-Paredes, M., Hagemann, S., Manchanda, H., Kristof, B., Gutekunst, J., Raddatz, G., Haas, R., Terstegen, L., Wenck, H., et al. (2016). Reduced DNA Methylation Patterning and Transcriptional Connectivity Define Human Skin Aging. Aging Cell *15*, 563–571. https://doi.org/10.1111/acel.12470.
27. Boroni, M., Zonari, A., Reis de Oliveira, C., Alkatib, K., Ochoa Cruz, E.A., Brace, L.E., and Lott de Carvalho, J. (2020). Highly Accurate Skin-Specific Methylome Analysis Algorithm as a Platform to Screen and Validate Therapeutics for Healthy Aging. Clin. Epigenet. *12*, 105. https://doi.org/10.1186/s13148-020-00899-1.
28. Debrabant, B., Soerensen, M., Christiansen, L., Tan, Q., McGue, M., Christensen, K., and Hjelmborg, J. (2018). DNA Methylation Age and Perceived Age in Elderly Danish Twins. Mech. Ageing Dev. *169*, 40–44. https://doi.org/10.1016/j.mad.2017.09.004.
29. Landi, M.T., Bishop, D.T., MacGregor, S., Machiela, M.J., Stratigos, A.J., Ghiorzo, P., Brossard, M., Calista, D., Choi, J., Fargnoli, M.C., et al. (2020). Genome-Wide Association Meta-Analyses Combining Multiple Risk Phenotypes Provide Insights into the Genetic Architecture of Cutaneous Melanoma Susceptibility. Nat. Genet. *52*, 494–504. https://doi.org/10.1038/s41588-020-0611-8.
30. Duffy, D.L., Zhu, G., Li, X., Sanna, M., Iles, M.M., Jacobs, L.C., Evans, D.M., Yazar, S., Beesley, J., Law, M.H., et al. (2018). Novel Pleiotropic Risk Loci for Melanoma and Nevus Density Implicate Multiple Biological Pathways. Nat. Commun. *9*, 4774. https://doi.org/10.1038/s41467-018-06649-5.
31. Liu, F., Hamer, M.A., Deelen, J., Lall, J.S., Jacobs, L., van Heemst, D., Murray, P.G., Wollstein, A., de Craen, A.J.M., Uh, H.-W., et al. (2016). The MC1R Gene and Youthful Looks. Curr. Biol. *26*, 1213–1220. https://doi.org/10.1016/j.cub.2016.03.008.
32. Law, M.H., Medland, S.E., Zhu, G., Yazar, S., Viñuela, A., Wallace, L., Shekar, S.N., Duffy, D.L., Bataille, V., Glass, D., et al. (2017). Genome-Wide Association Shows That Pigmentation Genes Play a Role in Skin Aging. J. Invest. Dermatol. *137*, 1887–1894. https://doi.org/10.1016/j.jid.2017.04.026.
33. Laville, V., Le Clerc, S., Ezzedine, K., Jdid, R., Taing, L., Labib, T., Coulonges, C., Ulveling, D., Galan, P., Guinot, C., et al. (2019). A Genome Wide Association Study Identifies New Genes Potentially Associated with Eyelid Sagging. Exp. Dermatol. *28*, 892–898. https://doi.org/10.1111/exd.13559.
34. Le Clerc, S., Taing, L., Ezzedine, K., Latreille, J., Delaneau, O., Labib, T., Coulonges, C., Bernard, A., Melak, S., Carpentier, W., et al. (2013). A Genome-Wide Association Study in Caucasian Women Points Out a Putative Role of the STXBP5L Gene in Facial Photoaging. J. Invest. Dermatol. *133*, 929–935. https://doi.org/10.1038/jid.2012.458.
35. Verdi, S., Abbasian, G., Bowyer, R.C.E., Lachance, G., Yarand, D., Christofidou, P., Mangino, M., Menni, C., Bell, J.T., Falchi, M., et al. (2019). TwinsUK: The UK Adult Twin Registry Update. Twin Res. Hum. Genet. *22*, 523–529. https://doi.org/10.1017/thg.2019.65.
36. Andrew, T., Hart, D.J., Snieder, H., Lange, M. de, Spector, T.D., and MacGregor, A.J. (2001). Are Twins and Singletons Comparable? A Study of Disease-related and Lifestyle Characteristics in Adult Women. Twin Res. *4*, 464–477. https://doi.org/10.1375/twin.4.6.464.
37. Buil, A., Brown, A.A., Lappalainen, T., Viñuela, A., Davies, M.N., Zheng, H., Richards, J., Glass, D., Small, K.S., Durbin, R., et al. (2015). Gene-Gene and Gene-Environment Interactions Detected by Transcriptome Sequence Analysis in Twins. Nat. Genet. *47*, 88–91. https://doi.org/10.1038/ng.3162.
38. Zhou, W., Laird, P.W., and Shen, H. (2017). Comprehensive Characterization, Annotation and Innovative Use of Infinium DNA Methylation BeadChip Probes. Nucleic Acids Res. *45*, e22. https://doi.org/10.1093/nar/gkw967.
39. Xu, Z., Niu, L., Li, L., and Taylor, J.A. (2016). ENmix: A Novel Background Correction Method for Illumina HumanMethylation450 BeadChip. Nucleic Acids Res. *44*, e20. https://doi.org/10.1093/nar/gkv907.
40. Sala, C., Di Lena, P., Fernandes Durso, D., Prodi, A., Castellani, G., and Nardini, C. (2020). Evaluation of Pre-Processing on the Meta-Analysis of DNA Methylation Data from the Illumina HumanMethylation450 BeadChip Platform. PLoS One *15*, e0229763. https://doi.org/10.1371/journal.pone.0229763.
41. Teschendorff, A.E., Zhu, T., Breeze, C.E., and Beck, S. (2020). EPISCORE: Cell Type Deconvolution of Bulk Tissue DNA Methylomes from Single-Cell RNA-Seq Data. Genome Biol. *21*, 221. https://doi.org/10.1186/s13059-020-02126-9.
42. Zhu, T., Liu, J., Beck, S., Pan, S., Capper, D., Lechner, M., Thirlwell, C., Breeze, C.E., and Teschendorff, A.E. (2022). A Pan-Tissue DNA Methylation Atlas Enables in Silico Decomposition of Human Tissue Methylomes at Cell-Type Resolution. Nat. Methods *19*, 296–306. https://doi.org/10.1038/s41592-022-01412-7.
43. El-Sayed Moustafa, J.S., Jackson, A.U., Brotman, S.M., Guan, L., Villicaña, S., Roberts, A.L., Zito, A., Bonnycastle, L., Erdos, M.R., Narisu, N., et al. (2022). ACE2 Expression in Adipose Tissue Is Associated with Cardio-Metabolic Risk Factors and

Cell Type Composition—Implications for COVID-19. Int. J. Obes. *46*, 1478–1486. https://doi.org/10.1038/s41366-022-01136-w.
44. Dobin, A., Davis, C.A., Schlesinger, F., Drenkow, J., Zaleski, C., Jha, S., Batut, P., Chaisson, M., and Gingeras, T.R. (2013). STAR: Ultrafast Universal RNA-seq Aligner. Bioinformatics *29*, 15–21. https://doi.org/10.1093/bioinformatics/bts635.
45. Delaneau, O., Ongen, H., Brown, A.A., Fort, A., Panousis, N.I., and Dermitzakis, E.T. (2017). A Complete Tool Set for Molecular QTL Discovery and Analysis. Nat. Commun. *8*, 15452. https://doi.org/10.1038/ncomms15452.
46. Frankish, A., Diekhans, M., Ferreira, A.-M., Johnson, R., Jungreis, I., Loveland, J., Mudge, J.M., Sisu, C., Wright, J., Armstrong, J., et al. (2019). GENCODE Reference Annotation for the Human and Mouse Genomes. Nucleic Acids Res. *47*, D766–D773. https://doi.org/10.1093/nar/gky955.
47. Hysi, P.G., Mangino, M., Christofidou, P., Falchi, M., Karoly, E.D., Mohney, R.P., Valdes, A.M., Spector, T.D., Menni, C., and Menni, C. (2022). Metabolome Genome-Wide Association Study Identifies 74 Novel Genomic Regions Influencing Plasma Metabolites Levels. Metabolites *12*, 61. https://doi.org/10.3390/metabo12010061.
48. Teo, Y.Y., Inouye, M., Small, K.S., Gwilliam, R., Deloukas, P., Kwiatkowski, D.P., and Clark, T.G. (2007). A Genotype Calling Algorithm for the Illumina BeadArray Platform. Bioinformatics *23*, 2741–2746. https://doi.org/10.1093/bioinformatics/btm443.
49. Neale, M.C., Hunter, M.D., Pritikin, J.N., Zahery, M., Brick, T.R., Kirkpatrick, R.M., Estabrook, R., Bates, T.C., Maes, H.H., and Boker, S.M. (2016). OpenMx 2.0: Extended Structural Equation and Statistical Modeling. Psychometrika *81*, 535–549. https://doi.org/10.1007/s11336-014-9435-8.
50. Gogarten, S.M., Sofer, T., Chen, H., Yu, C., Brody, J.A., Thornton, T.A., Rice, K.M., and Conomos, M.P. (2019). Genetic Association Testing Using the GENESIS R/Bioconductor Package. Bioinformatics *35*, 5346–5348. https://doi.org/10.1093/bioinformatics/btz567.
51. Aulchenko, Y.S., Ripke, S., Isaacs, A., and Van Duijn, C.M. (2007). GenABEL: An R Library for Genome-Wide Association Analysis. Bioinformatics *23*, 1294–1296. https://doi.org/10.1093/bioinformatics/btm108.
52. Shabalin, A.A. (2012). Matrix eQTL: Ultra Fast eQTL Analysis via Large Matrix Operations. Bioinformatics *28*, 1353–1358. https://doi.org/10.1093/bioinformatics/bts163.
53. Yang, J., Lee, S.H., Goddard, M.E., and Visscher, P.M. (2011). GCTA: A Tool for Genome-Wide Complex Trait Analysis. Am. J. Hum. Genet. *88*, 76–82. https://doi.org/10.1016/j.ajhg.2010.11.011.
54. Yang, J., Ferreira, T., Morris, A.P., Medland, S.E., Madden, P.A.F., Heath, A.C., Martin, N.G., Montgomery, G.W., Weedon, M.N., Loos, R.J., et al. (2012). Conditional and Joint Multiple-SNP Analysis of GWAS Summary Statistics Identifies Additional Variants Influencing Complex Traits. Nat. Genet. *44*, 369–S3. https://doi.org/10.1038/ng.2213.
55. Quinlan, A.R., and Hall, I.M. (2010). BEDTools: A Flexible Suite of Utilities for Comparing Genomic Features. Bioinformatics *26*, 841–842. https://doi.org/10.1093/bioinformatics/btq033.
56. Karolchik, D., Hinrichs, A.S., Furey, T.S., Roskin, K.M., Sugnet, C.W., Haussler, D., and Kent, W.J. (2004). The UCSC Table Browser Data Retrieval Tool. Nucleic Acids Res. *32*, D493–D496. https://doi.org/10.1093/nar/gkh103.
57. Brenet, F., Moh, M., Funk, P., Feierstein, E., Viale, A.J., Socci, N.D., and Scandura, J.M. (2011). DNA Methylation of the First Exon Is Tightly Linked to Transcriptional Silencing. PLoS One *6*, e14524. https://doi.org/10.1371/journal.pone.0014524.
58. Sheffield, N.C., and Bock, C. (2016). LOLA: Enrichment Analysis for Genomic Region Sets and Regulatory Elements in R and Bioconductor. Bioinformatics *32*, 587–589. https://doi.org/10.1093/bioinformatics/btv612.
59. Kundaje, A., Meuleman, W., Ernst, J., Bilenky, M., Yen, A., Kheradpour, P., Zhang, Z., Heravi-Moussavi, A., Liu, Y., Amin, V., et al. (2015). Integrative Analysis of 111 Reference Human Epigenomes. Nature *518*, 317–330. https://doi.org/10.1038/nature14248.
60. Boix, C.A., James, B.T., Park, Y.P., Meuleman, W., and Kellis, M. (2021). Regulatory Genomic Circuitry of Human Disease Loci by Integrative Epigenomics. Nature *590*, 300–307. https://doi.org/10.1038/s41586-020-03145-z.
61. Battram, T., Yousefi, P., Crawford, G., Prince, C., Sheikhali Babaei, M., Sharp, G., Hatcher, C., Vega-Salas, M.J., Khodabakhsh, S., Whitehurst, O., et al. (2022). The EWAS Catalog: A Database of Epigenome-Wide Association Studies. Wellcome Open Res. *7*, 41. https://doi.org/10.12688/wellcomeopenres.17598.2.
62. Li, M., Zou, D., Li, Z., Gao, R., Sang, J., Zhang, Y., Li, R., Xia, L., Zhang, T., Niu, G., et al. (2019). EWAS Atlas: A Curated Knowledgebase of Epigenome-Wide Association Studies. Nucleic Acids Res. *47*, D983–D988. https://doi.org/10.1093/nar/gky1027.
63. Xiong, Z., Yang, F., Li, M., Ma, Y., Zhao, W., Wang, G., Li, Z., Zheng, X., Zou, D., Zong, W., et al. (2022). EWAS Open Platform: Integrated Data, Knowledge and Toolkit for Epigenome-Wide Association Study. Nucleic Acids Res. *50*, D1004–D1009. https://doi.org/10.1093/nar/gkab972.
64. Sollis, E., Mosaku, A., Abid, A., Buniello, A., Cerezo, M., Gil, L., Groza, T., Güneş, O., Hall, P., Hayhurst, J., et al. (2023). The NHGRI-EBI GWAS Catalog: Knowledgebase and Deposition Resource. Nucleic Acids Res. *51*, D977–D985. https://doi.org/10.1093/nar/gkac1010.
65. Storey, J.D., and Tibshirani, R. (2003). Statistical Significance for Genomewide Studies. USA *100*, 9440–9445. https://doi.org/10.1073/pnas.1530509100.
66. Qi, T., Wu, Y., Zeng, J., Zhang, F., Xue, A., Jiang, L., Zhu, Z., Kemper, K., Yengo, L., Zheng, Z., et al. (2018). Identifying Gene Targets for Brain-Related Traits Using Transcriptomic and Methylomic Data from Blood. Nat. Commun. *9*, 2282. https://doi.org/10.1038/s41467-018-04558-1.
67. Stegle, O., Parts, L., Piipari, M., Winn, J., and Durbin, R. (2012). Using Probabilistic Estimation of Expression Residuals (PEER) to Obtain Increased Power and Interpretability of Gene Expression Analyses. Nat. Protoc. *7*, 500–507. https://doi.org/10.1038/nprot.2011.457.
68. Aguet, F., Brown, A.A., Castel, S.E., Davis, J.R., He, Y., Jo, B., Mohammadi, P., Park, Y., Parsana, P., Segrè, A.V., et al. (2017). Genetic Effects on Gene Expression across Human Tissues. Nature *550*, 204–213. https://doi.org/10.1038/nature24277.
69. Giambartolomei, C., Vukcevic, D., Schadt, E.E., Franke, L., Hingorani, A.D., Wallace, C., and Plagnol, V. (2014). Bayesian Test for Colocalisation between Pairs of Genetic Association

Studies Using Summary Statistics. PLoS Genet. *10*, e1004383. https://doi.org/10.1371/journal.pgen.1004383.
70. Wallace, C. (2021). A More Accurate Method for Colocalisation Analysis Allowing for Multiple Causal Variants. PLoS Genet. *17*, e1009440. https://doi.org/10.1371/journal.pgen.1009440.
71. Guo, H., Fortune, M.D., Burren, O.S., Schofield, E., Todd, J.A., and Wallace, C. (2015). Integration of Disease Association and eQTL Data Using a Bayesian Colocalisation Approach Highlights Six Candidate Causal Genes in Immune-Mediated Diseases. Hum. Mol. Genet. *24*, 3305–3313. https://doi.org/10.1093/hmg/ddv077.
72. THE GTEX CONSORTIUM (2020). The GTEx Consortium Atlas of Genetic Regulatory Effects across Human Tissues. Science *369*, 1318–1330. https://doi.org/10.1126/science.aaz1776.
73. Mancuso, P., Tricarico, R., Bhattacharjee, V., Cosentino, L., Kadariya, Y., Jelinek, J., Nicolas, E., Einarson, M., Beeharry, N., Devarajan, K., et al. (2019). Thymine DNA Glycosylase as a Novel Target for Melanoma. Oncogene *38*, 3710–3728. https://doi.org/10.1038/s41388-018-0640-2.
74. Li, H., Liefke, R., Jiang, J., Kurland, J.V., Tian, W., Deng, P., Zhang, W., He, Q., Patel, D.J., Bulyk, M.L., et al. (2017). Polycomb-like Proteins Link the PRC2 Complex to CpG Islands. Nature *549*, 287–291. https://doi.org/10.1038/nature23881.
75. Kerimov, N., Hayhurst, J.D., Peikova, K., Manning, J.R., Walter, P., Kolberg, L., Samoviča, M., Sakthivel, M.P., Kuzmin, I., Trevanion, S.J., et al. (2021). A Compendium of Uniformly Processed Human Gene Expression and Splicing Quantitative Trait Loci. Nat. Genet. *53*, 1290–1299. https://doi.org/10.1038/s41588-021-00924-w.
76. Wang, G., Sarkar, A., Carbonetto, P., and Stephens, M. (2020). A Simple New Approach to Variable Selection in Regression, with Application to Genetic Fine Mapping. J. R. Stat. Soc. Series B Stat. Methodol. *82*, 1273–1300. https://doi.org/10.1111/rssb.12388.
77. Oshima, J., Sidorova, J.M., and Monnat, R.J. (2017). Werner Syndrome: Clinical Features, Pathogenesis and Potential Therapeutic Interventions. Ageing Res. Rev. *33*, 105–114. https://doi.org/10.1016/j.arr.2016.03.002.
78. Lønnberg, A.S., Skov, L., Skytthe, A., Kyvik, K.O., Pedersen, O.B., and Thomsen, S.F. (2013). Heritability of Psoriasis in a Large Twin Sample. Br. J. Dermatol. *169*, 412–416. https://doi.org/10.1111/bjd.12375.
79. Block, S.R., Winfield, J.B., Lockshin, M.D., D'Angelo, W.A., and Christian, C.L. (1975). Studies of Twins with Systemic Lupus Erythematosus. A Review of the Literature and Presentation of 12 Additional Sets. Am. J. Med. *59*, 533–552. https://doi.org/10.1016/0002-9343(75)90261-2.
80. Thomsen, S.F., Ulrik, C.S., Kyvik, K.O., Hjelmborg, J.v.B., Skadhauge, L.R., Steffensen, I., and Backer, V. (2007). Importance of Genetic Factors in the Etiology of Atopic Dermatitis: A Twin Study. Allergy Asthma Proc. *28*, 535–539. https://doi.org/10.2500/aap2007.28.3041.
81. Metri, R., Mohan, A., Nsengimana, J., Pozniak, J., Molina-Paris, C., Newton-Bishop, J., Bishop, D., and Chandra, N. (2017). Identification of a Gene Signature for Discriminating Metastatic from Primary Melanoma Using a Molecular Interaction Network Approach. Sci. Rep. *7*, 17314. https://doi.org/10.1038/s41598-017-17330-0.
82. Wang, J.Y., Chen, H., Wang, Y.Y., Wang, X.Q., Chen, H.Y., Zhang, M., Tang, Y., and Zhang, B. (2017). Network Pharmacological Mechanisms of Vernonia Anthelmintica (L.) in the Treatment of Vitiligo: Isorhamnetin Induction of Melanogenesis via up-Regulation of Melanin-Biosynthetic Genes. BMC Syst. Biol. *11*, 103. https://doi.org/10.1186/s12918-017-0486-1.
83. Setkowicz, M., Mastalerz, L., Gielicz, A., Wojas-Pelc, A., and Sanak, M. (2015). Lack of Association of ALOX12 and ALOX15B Polymorphisms with Psoriasis despite Altered Urinary Excretion of 12(S)-Hydroxyeicosatetraenoic Acid. Br. J. Dermatol. *172*, 337–344. https://doi.org/10.1111/bjd.13225.
84. Li, G., Gu, L., Zhao, F., Hu, Y., Wang, X., Zeng, F., Yu, J., Yue, C., Zhou, P., Li, Y., et al. (2023). WFDC12-overexpressing Contributes to the Development of Atopic Dermatitis via Accelerating ALOX12/15 Metabolism and PAF Accumulation. Cell Death Dis. *14*. https://doi.org/10.1038/s41419-023-05686-3.
85. Kim, S., Choi, I.F., Quante, J.R., Zhang, L., Roop, D.R., and Koster, M.I. (2009). P63 Directly Induces Expression of Alox12, a Regulator of Epidermal Barrier Formation. Exp. Dermatol. *18*, 1016–1021. https://doi.org/10.1111/j.1600-0625.2009.00894.x.
86. Kim, J.Y., Choi, B.-G., Jelinek, J., Kim, D.H., Lee, S.H., Cho, K., Rha, S.H., Lee, Y.H., Jin, H.S., Choi, D.-K., et al. (2020). Promoter Methylation Changes in ALOX12 and AIRE1: Novel Epigenetic Markers for Atherosclerosis. Clin. Epigenet. *12*, 66. https://doi.org/10.1186/s13148-020-00846-0.
87. Wan, J., Oliver, V.F., Wang, G., Zhu, H., Zack, D.J., Merbs, S.L., and Qian, J. (2015). Characterization of Tissue-Specific Differential DNA Methylation Suggests Distinct Modes of Positive and Negative Gene Expression Regulation. BMC Genom. *16*, 49. https://doi.org/10.1186/s12864-015-1271-4.
88. Rauluseviciute, I., Drabløs, F., and Rye, M.B. (2020). DNA Hypermethylation Associated with Upregulated Gene Expression in Prostate Cancer Demonstrates the Diversity of Epigenetic Regulation. BMC Med. Genom. *13*, 6. https://doi.org/10.1186/s12920-020-0657-6.
89. Price, M.A., Wanshura, L.E.C., Yang, J., Carlson, J., Xiang, B., Li, G., Ferrone, S., Dudek, A.Z., Turley, E.A., and McCarthy, J.B. (2011). CSPG4, a Potential Therapeutic Target, Facilitates Malignant Progression of Melanoma. Pigment Cell Melanoma Res. *24*, 1148–1157. https://doi.org/10.1111/j.1755-148X.2011.00929.x.
90. Harrer, D.C., Dörrie, J., and Schaft, N. (2019). CSPG4 as Target for CAR-T-Cell Therapy of Various Tumor Entities–Merits and Challenges. Int. J. Mol. Sci. *20*, 5942. https://doi.org/10.3390/ijms20235942.
91. ENCODE Project Consortium (2012). An Integrated Encyclopedia of DNA Elements in the Human Genome. Nature *489*, 57–74. https://doi.org/10.1038/nature11247.
92. Luo, Y., Hitz, B.C., Gabdank, I., Hilton, J.A., Kagda, M.S., Lam, B., Myers, Z., Sud, P., Jou, J., Lin, K., et al. (2020). New Developments on the Encyclopedia of DNA Elements (ENCODE) Data Portal. Nucleic Acids Res. *48*, D882–D889. https://doi.org/10.1093/nar/gkz1062.
93. Kichaev, G., Bhatia, G., Loh, P.-R., Gazal, S., Burch, K., Freund, M.K., Schoech, A., Pasaniuc, B., and Price, A.L. (2019). Leveraging Polygenic Functional Enrichment to Improve GWAS Power. Am. J. Hum. Genet. *104*, 65–75. https://doi.org/10.1016/j.ajhg.2018.11.008.
94. Liyanage, U.E., Law, M.H., Han, X., An, J., Ong, J.-S., Gharahkhani, P., Gordon, S., Neale, R.E., Olsen, C.M., et al.; 23andMe Research Team (2019). Combined Analysis of

Keratinocyte Cancers Identifies Novel Genome-Wide Loci. Hum. Mol. Genet. *28*, 3148–3160. https://doi.org/10.1093/hmg/ddz121.

95. Guen, V.J., Gamble, C., Lees, J.A., and Colas, P. (2017). The Awakening of the CDK10/Cyclin M Protein Kinase. Oncotarget *8*, 50174–50186. https://doi.org/10.18632/oncotarget.15024.
96. Baubec, T., Colombo, D.F., Wirbelauer, C., Schmidt, J., Burger, L., Krebs, A.R., Akalin, A., and Schübeler, D. (2015). Genomic Profiling of DNA Methyltransferases Reveals a Role for DNMT3B in Genic Methylation. Nature *520*, 243–247. https://doi.org/10.1038/nature14176.
97. Bonilla, C., Bertoni, B., Min, J.L., Hemani, G., Consortium, G.o.D.M., and Elliott, H.R. (2021). Investigating DNA Methylation as a Potential Mediator between Pigmentation Genes, Pigmentary Traits and Skin Cancer. Pigment Cell Melanoma Res. *34*, 892–904. https://doi.org/10.1111/pcmr.12948.
98. Villicaña, S., and Bell, J.T. (2021). Genetic Impacts on DNA Methylation: Research Findings and Future Perspectives. Genome Biol. *22*, 127. https://doi.org/10.1186/s13059-021-02347-6.
99. Schneider, S., Thurnher, D., Kloimstein, P., Leitner, V., Petzelbauer, P., Pammer, J., Brunner, M., and Erovic, B.M. (2011). Expression of the Sonic Hedgehog Pathway in Squamous Cell Carcinoma of the Skin and the Mucosa of the Head and Neck. Head Neck *33*, 244–250. https://doi.org/10.1002/hed.21437.
100. Xi, S., Zhang, Q., Dyer, K.F., Lerner, E.C., Smithgall, T.E., Gooding, W.E., Kamens, J., and Grandis, J.R. (2003). Src Kinases Mediate STAT Growth Pathways in Squamous Cell Carcinoma of the Head and Neck. J. Biol. Chem. *278*, 31574–31583. https://doi.org/10.1074/jbc.M303499200.
101. Wang, Y., Xie, Y., Sun, B., Guo, Y., Song, L., Mohammednur, D.E., and Zhao, C. (2021). The Degradation of Rap1GAP via E6AP-mediated Ubiquitin-Proteasome Pathway Is Associated with HPV16/18-Infection in Cervical Cancer Cells. Infect. Agents Cancer *16*, 71. https://doi.org/10.1186/s13027-021-00409-9.
102. Chandra, A., Senapati, S., Roy, S., Chatterjee, G., and Chatterjee, R. (2018). Epigenome-Wide DNA Methylation Regulates Cardinal Pathological Features of Psoriasis. Clin. Epigenet. *10*, 108. https://doi.org/10.1186/s13148-018-0541-9.
103. Ellinghaus, D., Jostins, L., Spain, S.L., Cortes, A., Bethune, J., Han, B., Park, Y.R., Raychaudhuri, S., Pouget, J.G., Hübenthal, M., et al. (2016). Analysis of Five Chronic Inflammatory Diseases Identifies 27 New Associations and Highlights Disease-Specific Patterns at Shared Loci. Nat. Genet. *48*, 510–518. https://doi.org/10.1038/ng.3528.

The American Journal of Human Genetics, Volume *111*

Supplemental information

Genetic effects on the skin methylome in healthy older twins

Christopher J. Shore, Sergio Villicaña, Julia S. El-Sayed Moustafa, Amy L. Roberts, David A. Gunn, Veronique Bataille, Panos Deloukas, Tim D. Spector, Kerrin S. Small, and Jordana T. Bell

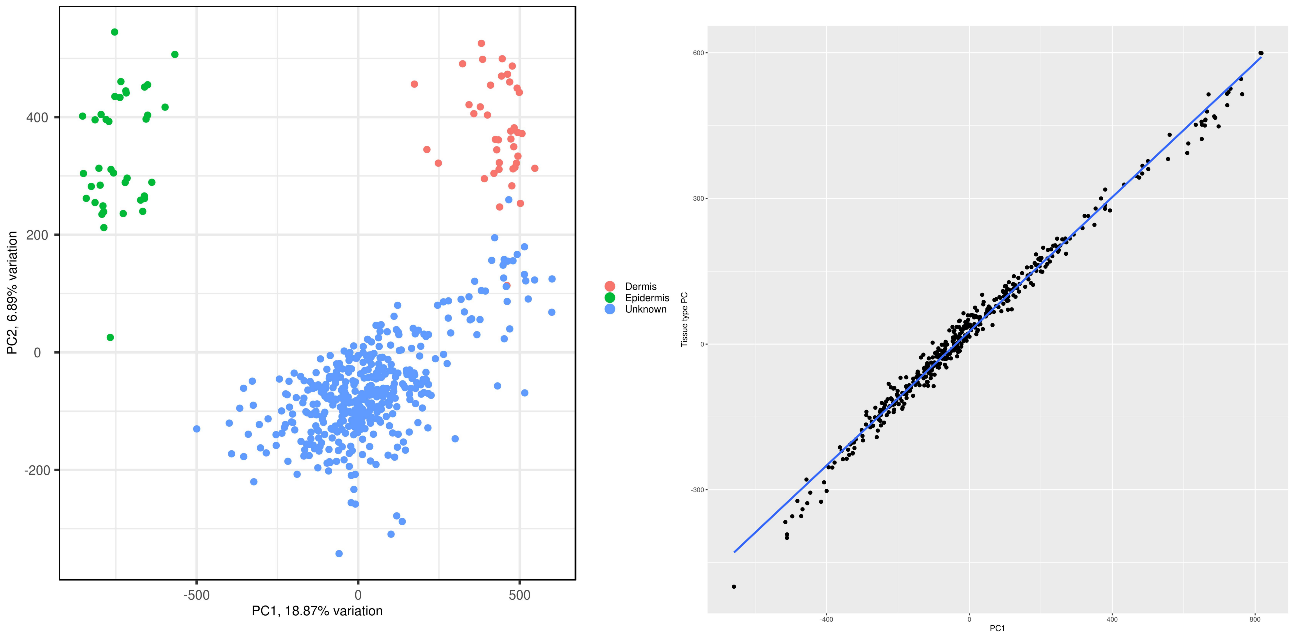

Figure S1. (a) Biplot of PC1 and PC2 from PCA of whole skin DNA methylation data from 414 samples, with dermis and epidermis DNA methylation data from 92 samples from Vandiver et al.[1] (b) Scatter plot of PC1 from S1a ("Tissue Type PC") with PC1 from PCA on just the 414 whole skin DNA methylation data.

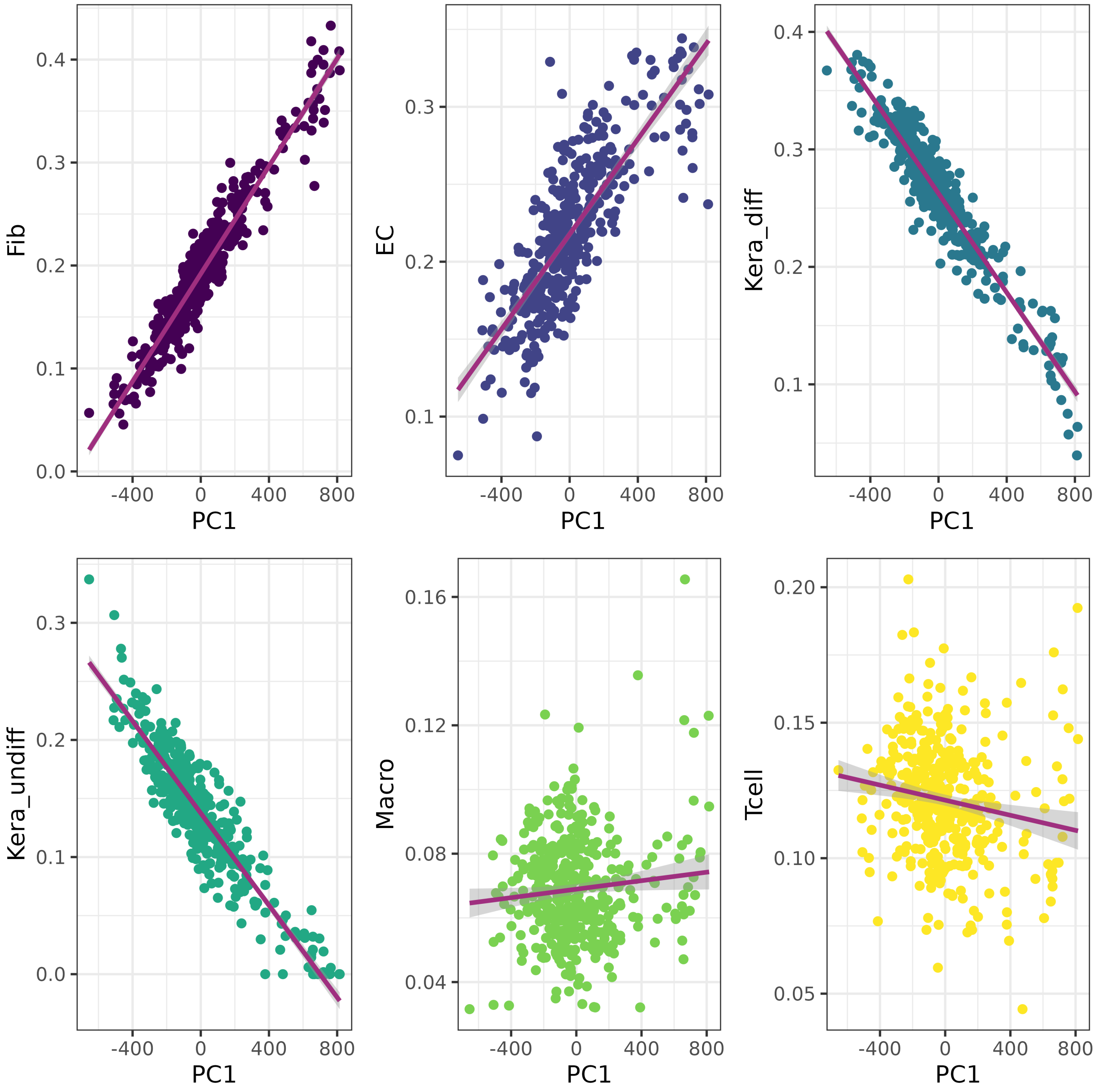

Figure S2. Scatter plots of PC1 from PCA of whole skin DNA methylation data from 414 samples vs EpiSCORE cell-type estimates for each sample.

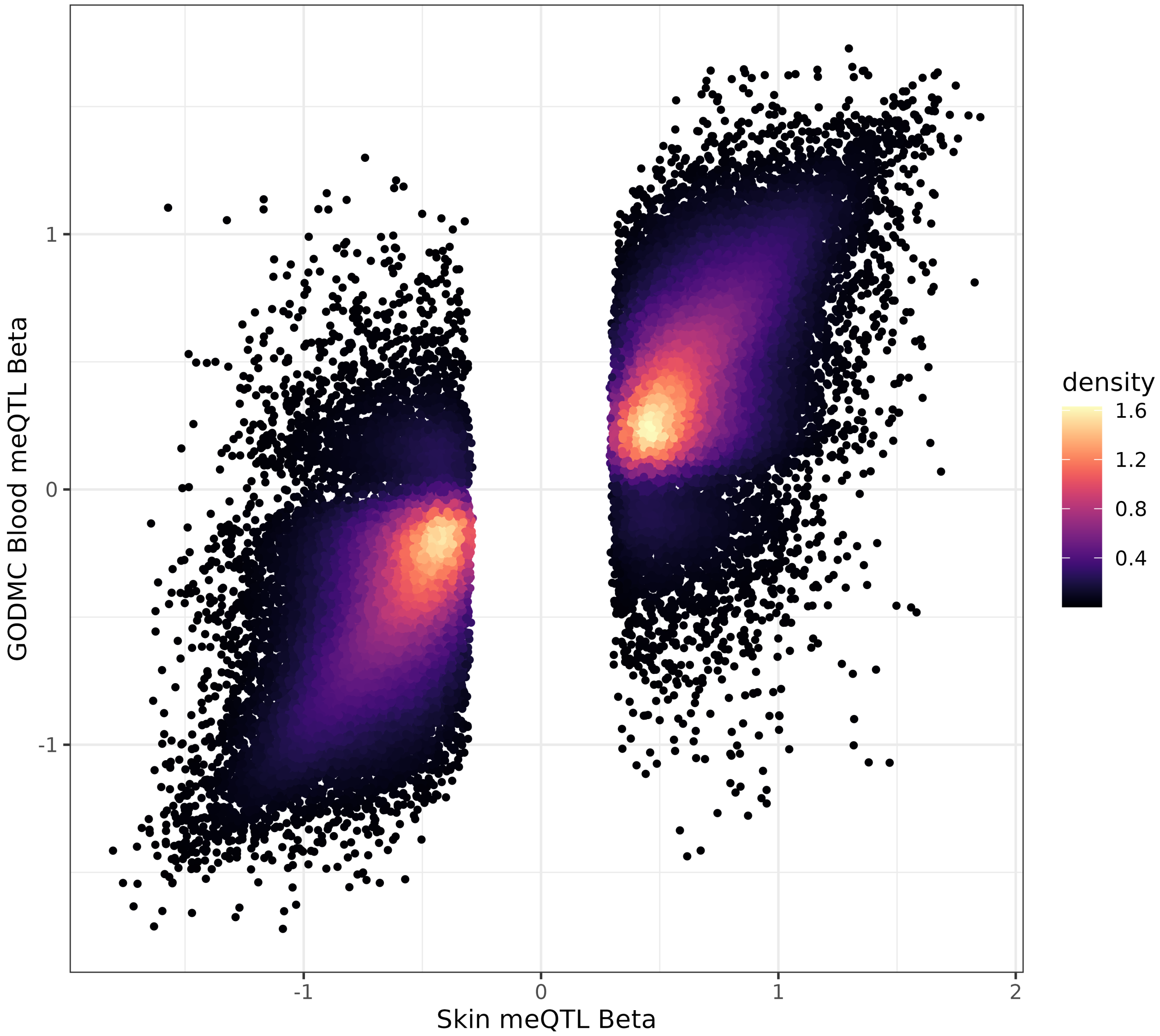

Figure S3. Scatter plot of significant whole skin *cis*-meQTL effect betas against blood *cis*-meQTL effect betas from Min et al.,[2], coloured by the density of points.

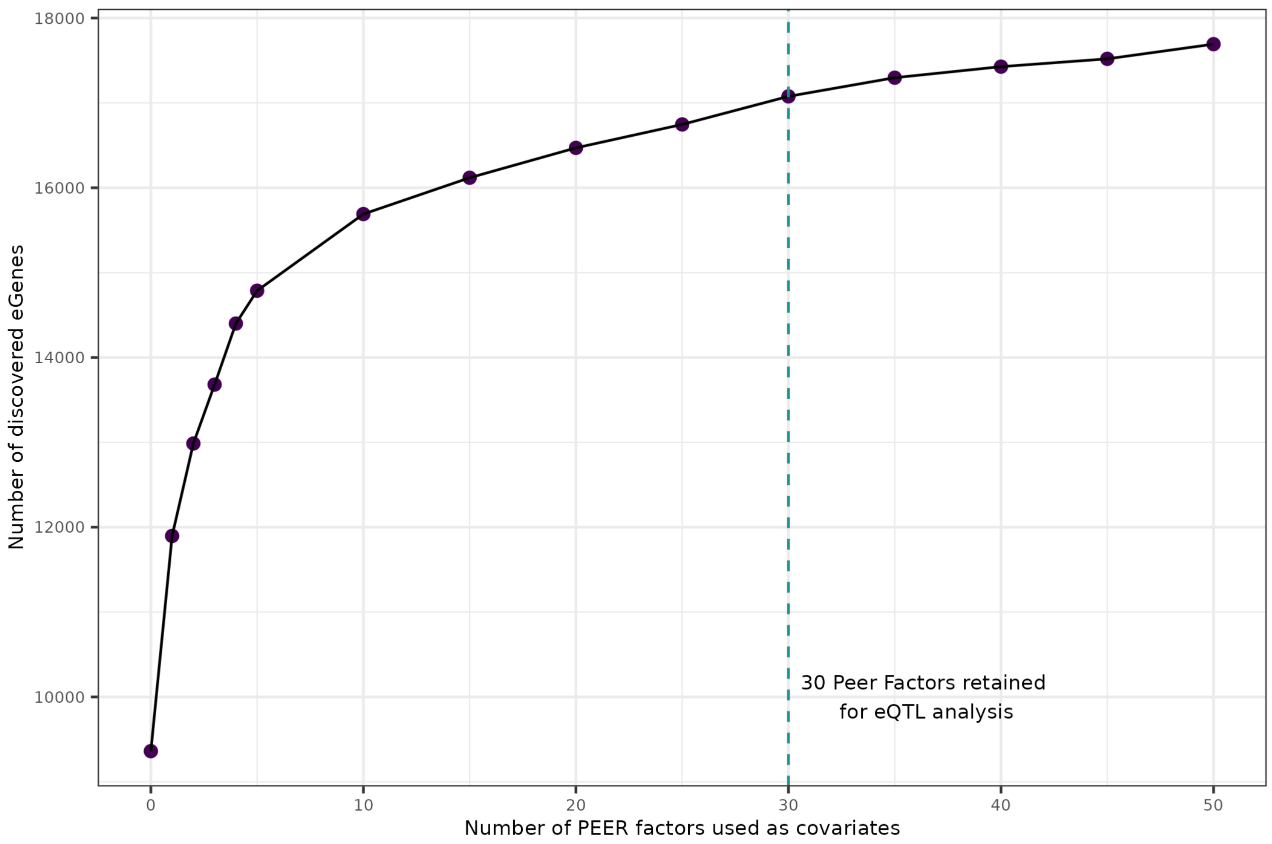

Figure S4. Number of discovered eGenes in whole skin at a Benjamini-Hochberg FDR of 5% in cis-eQTL analyses with different numbers of PEER factors included as covariates. 30 PEER factors were retained for our primary cis-eQTL analysis, to maximise eGene discovery whilst avoiding over-fitting.

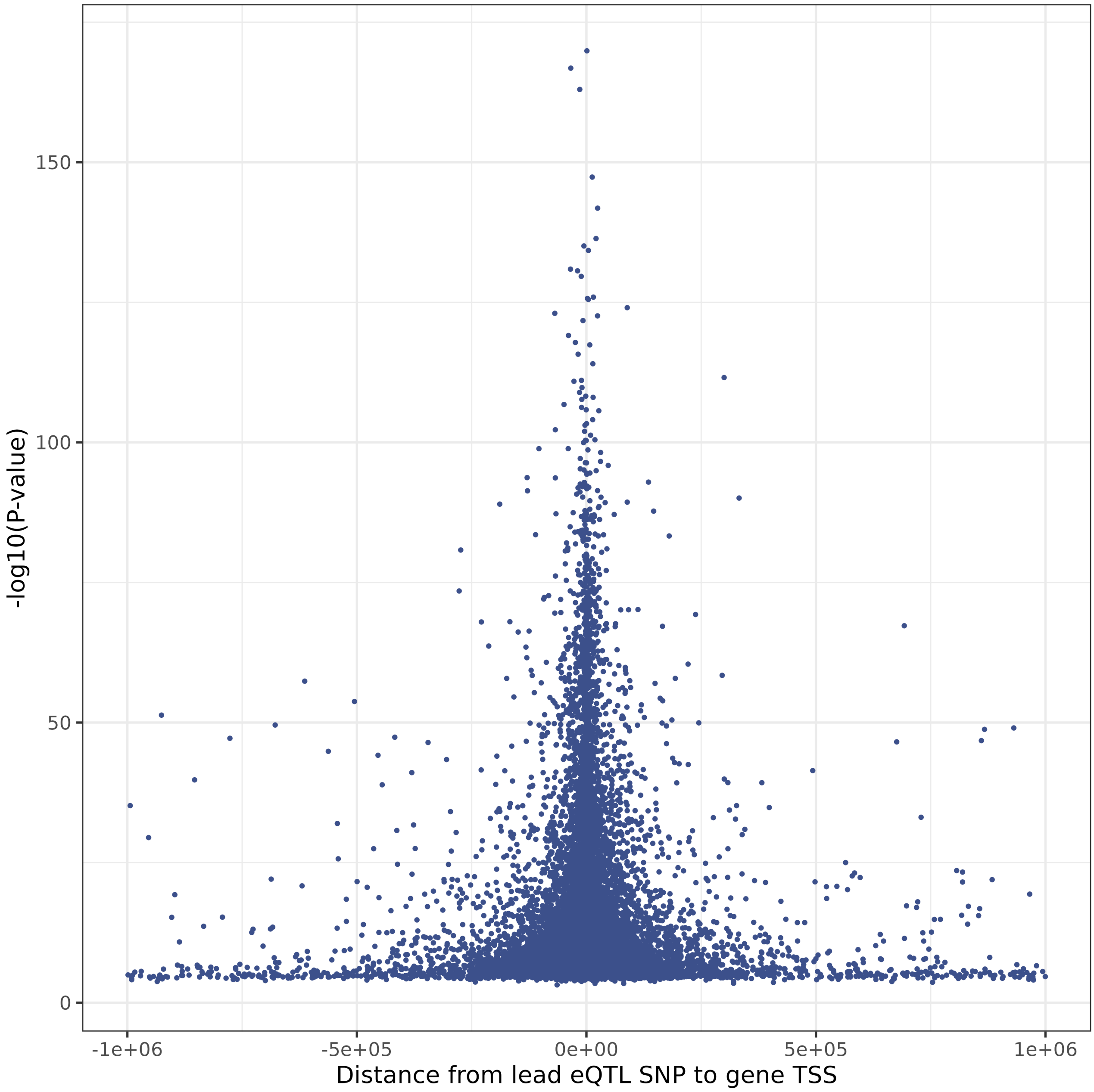

Figure S5. Distance from lead eQTL SNP from conditional analysis to TSS of its eGene vs -log10(P-value) of the eQTL association.

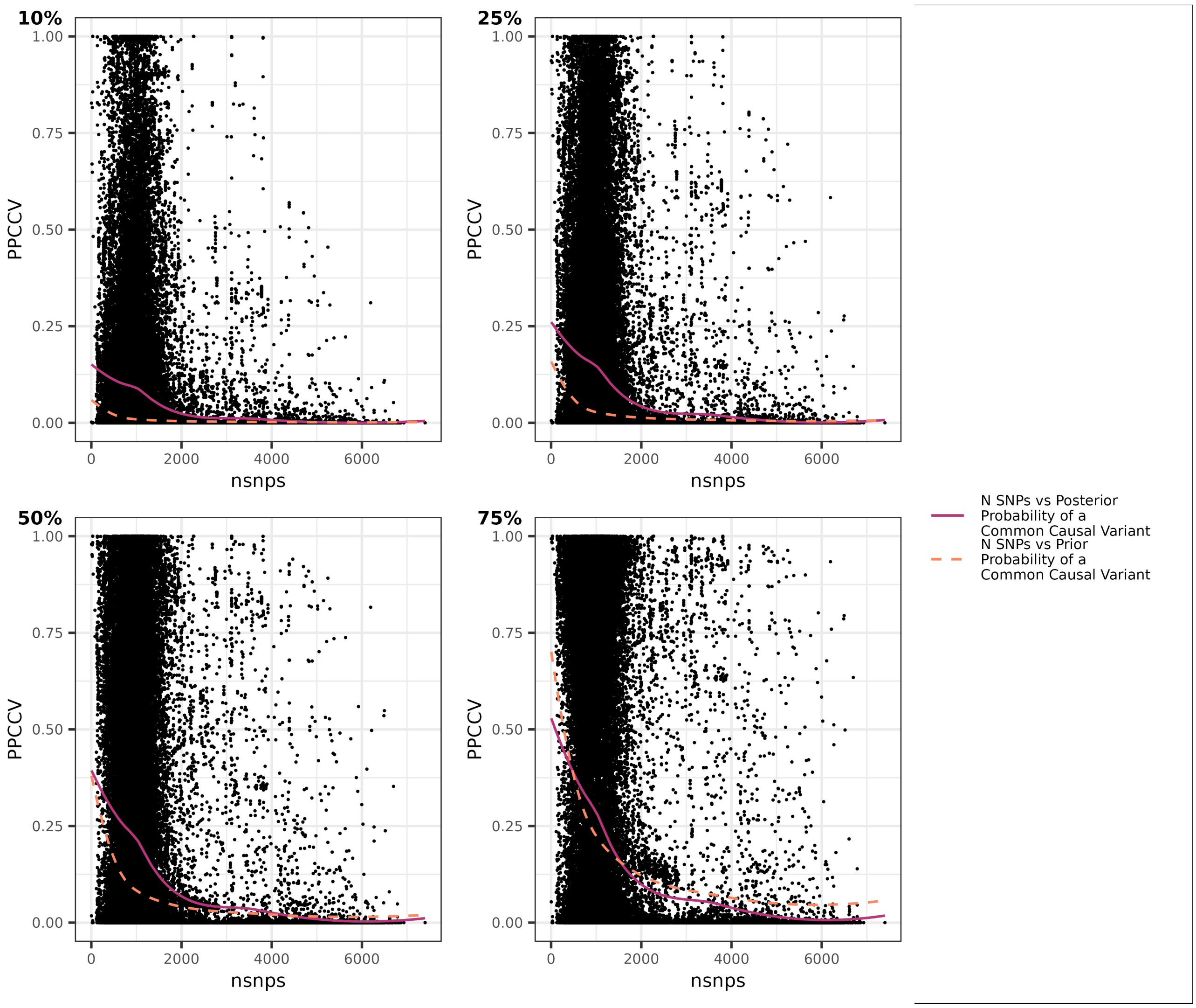

Figure S6. Plots used to find the optimal value of p12 in eQTL-meQTL co-localisation analysis. NSNPs vs observed Posterior Probability of a Common Causal Variant for p12 values corresponding to 10%, 25%, 50%, and 75% probability of a causal cis-eQTL SNP also being a causal cis-meQTL SNP. The purple curve is the loess smoothed curve of these points. The orange dashed line is the loess smoothed curve of NSNPs vs the Prior Probability of a Common Causal Variant (not plotted), calculated using the formula described by Guo *et al.*,[3].

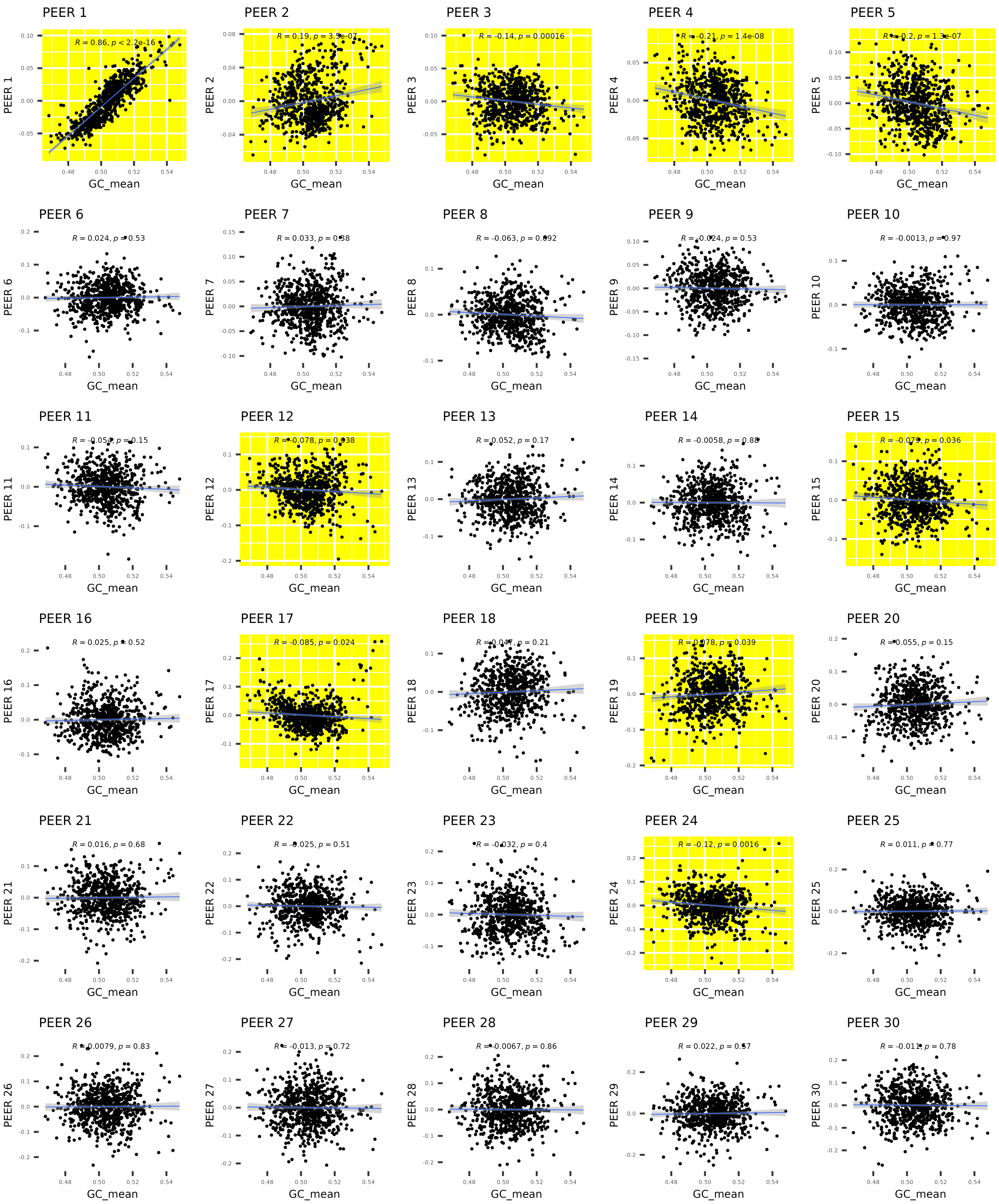

Figure S7. Scatter plots showing the correlation between the 30 PEER factors used as covariates in the skin eQTL analysis and the mean GC content per RNA sample. A yellow background indicates a nominally significant correlation (Pearson P<0.05).

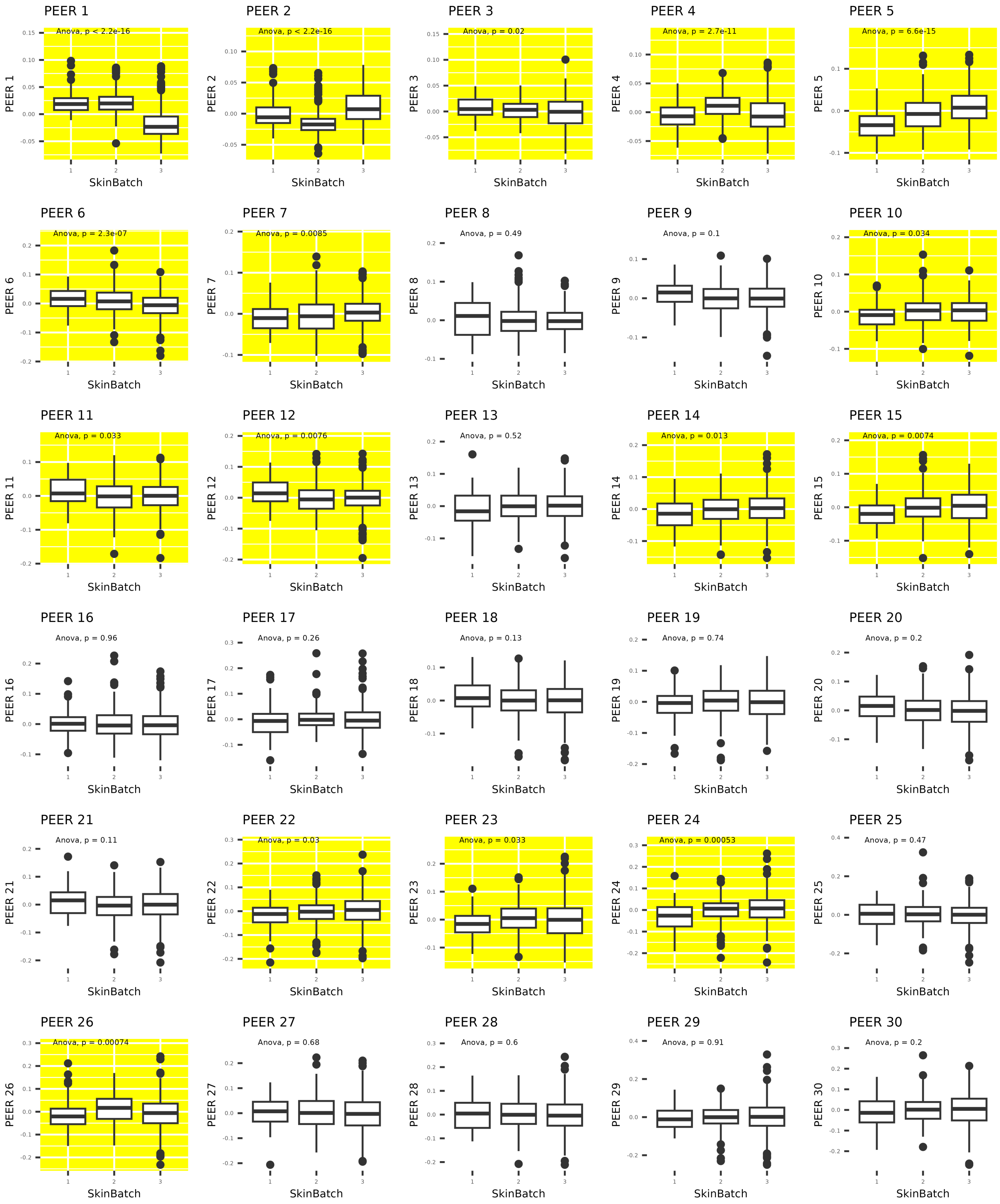

Figure S8. Box plots showing the correlation between the 30 PEER factors used as covariates in the skin eQTL analysis and the RNAseq batch. A yellow background indicates a nominally significant difference between groups (Anova P<0.05).

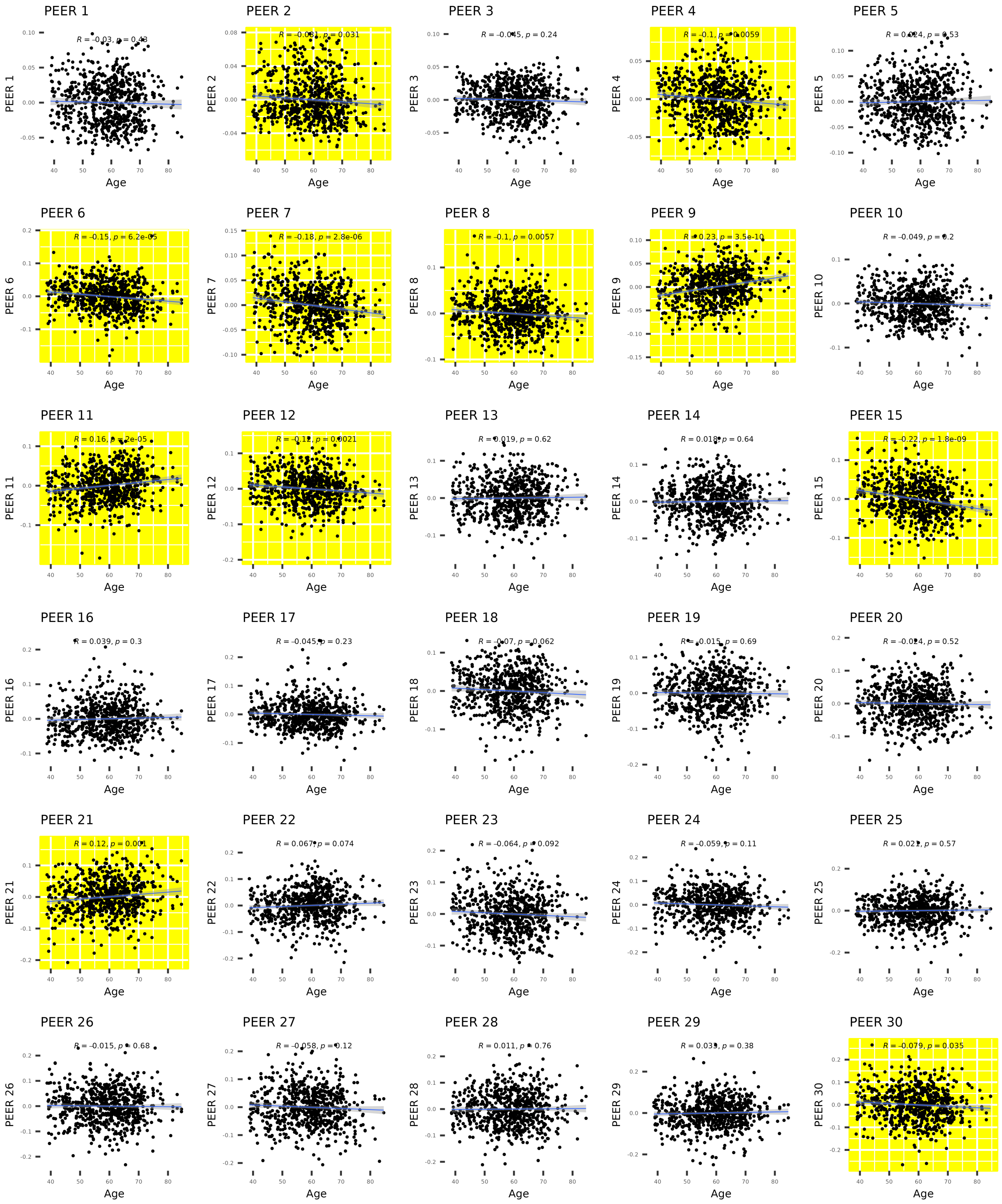

Figure S9. Scatter plots showing the correlation between the 30 PEER factors used as covariates in the skin eQTL analysis and chronological age. A yellow background indicates a nominally significant correlation (Pearson P<0.05).

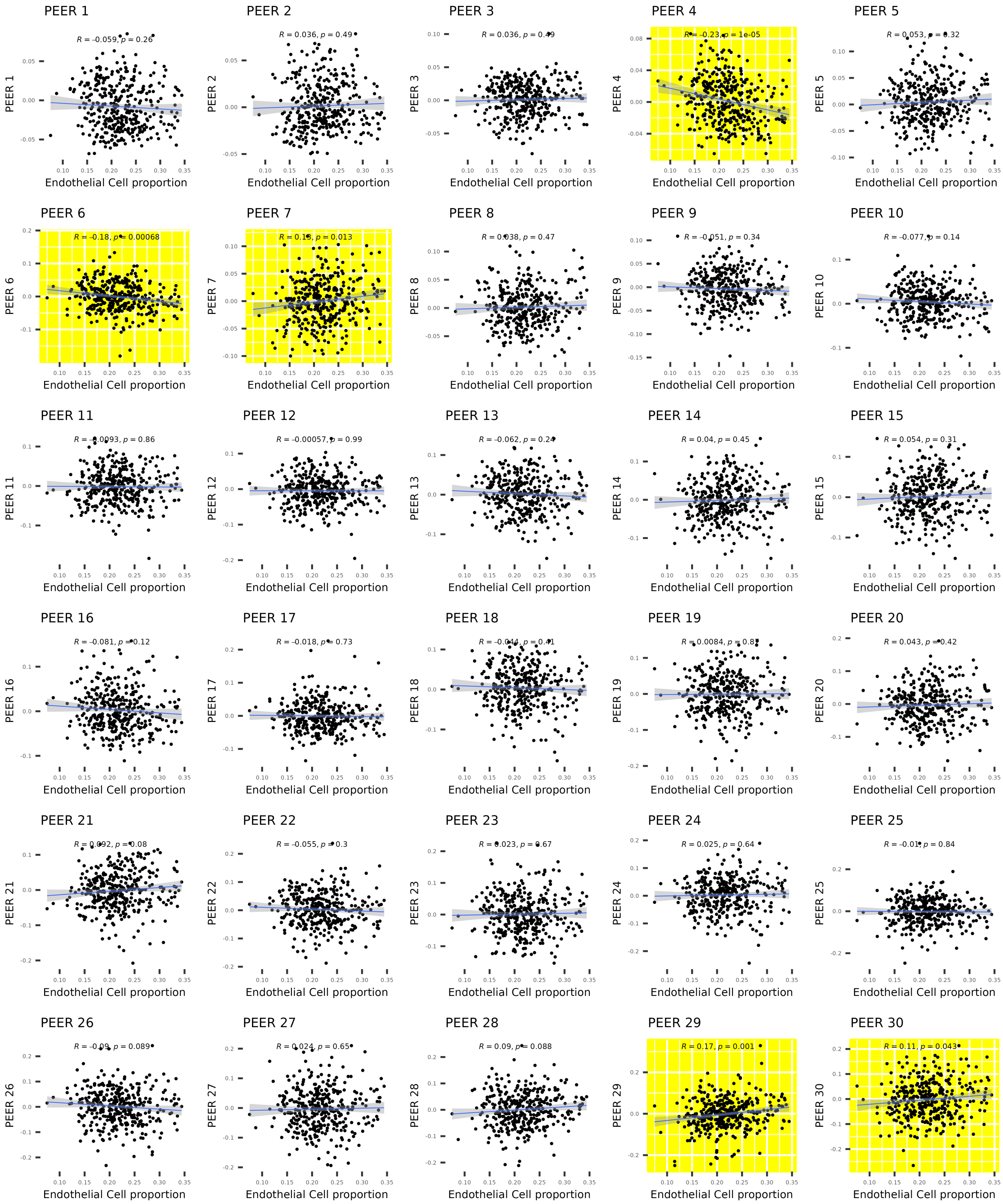

Figure S10. Scatter plots showing the correlation between the 30 PEER factors used as covariates in the skin eQTL analysis and Endothelial Cell proportion as estimated from DNA methylation data. A yellow background indicates a nominally significant correlation (Pearson P<0.05).

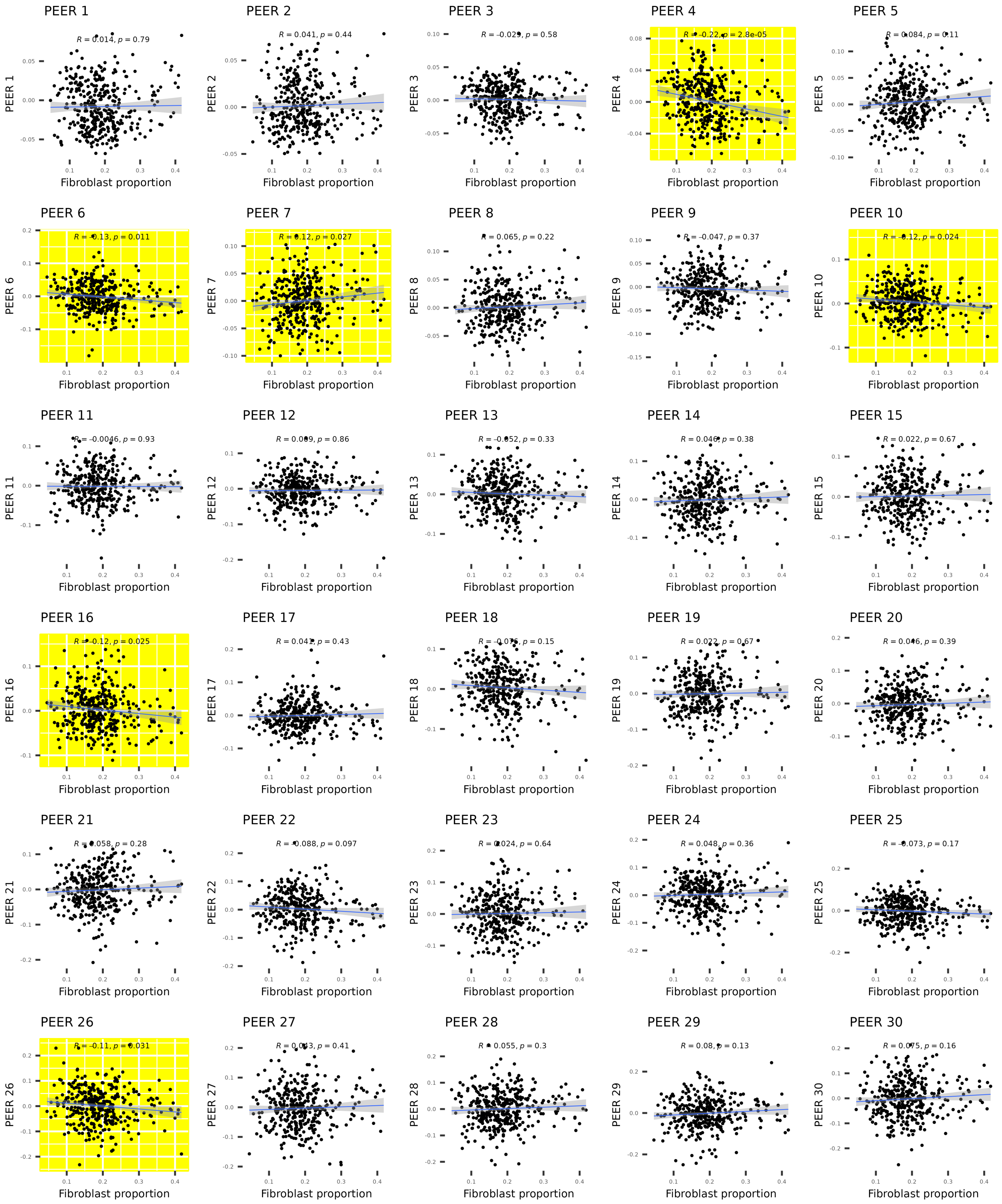

Figure S11. Scatter plots showing the correlation between the 30 PEER factors used as covariates in the skin eQTL analysis and Fibroblast Cell proportion as estimated from DNA methylation data. A yellow background indicates a nominally significant correlation (Pearson P<0.05).

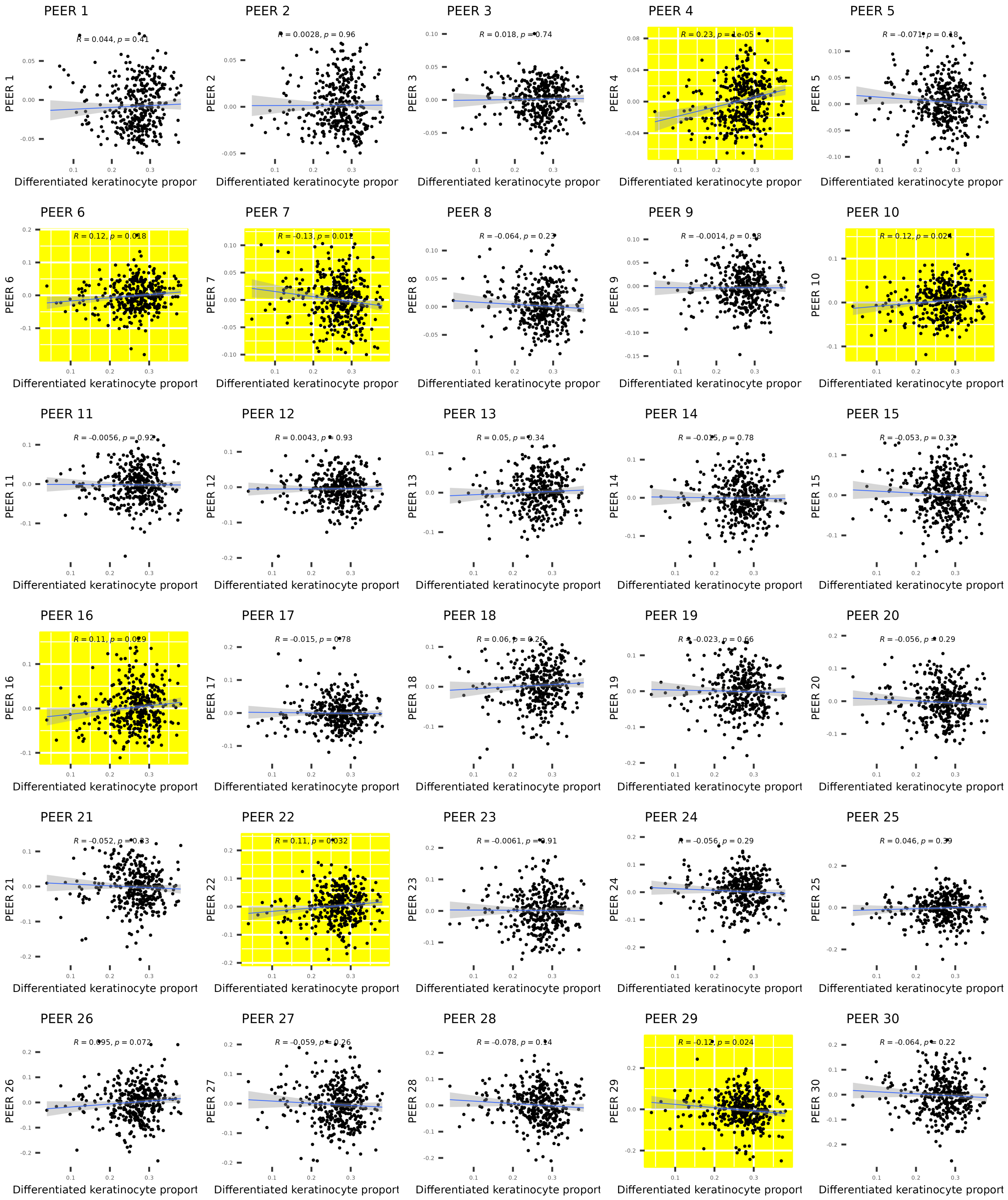

Figure S12. Scatter plots showing the correlation between the 30 PEER factors used as covariates in the skin eQTL analysis and Differentiated Keratinocyte Cell proportion as estimated from DNA methylation data. A yellow background indicates a nominally significant correlation (Pearson P<0.05).

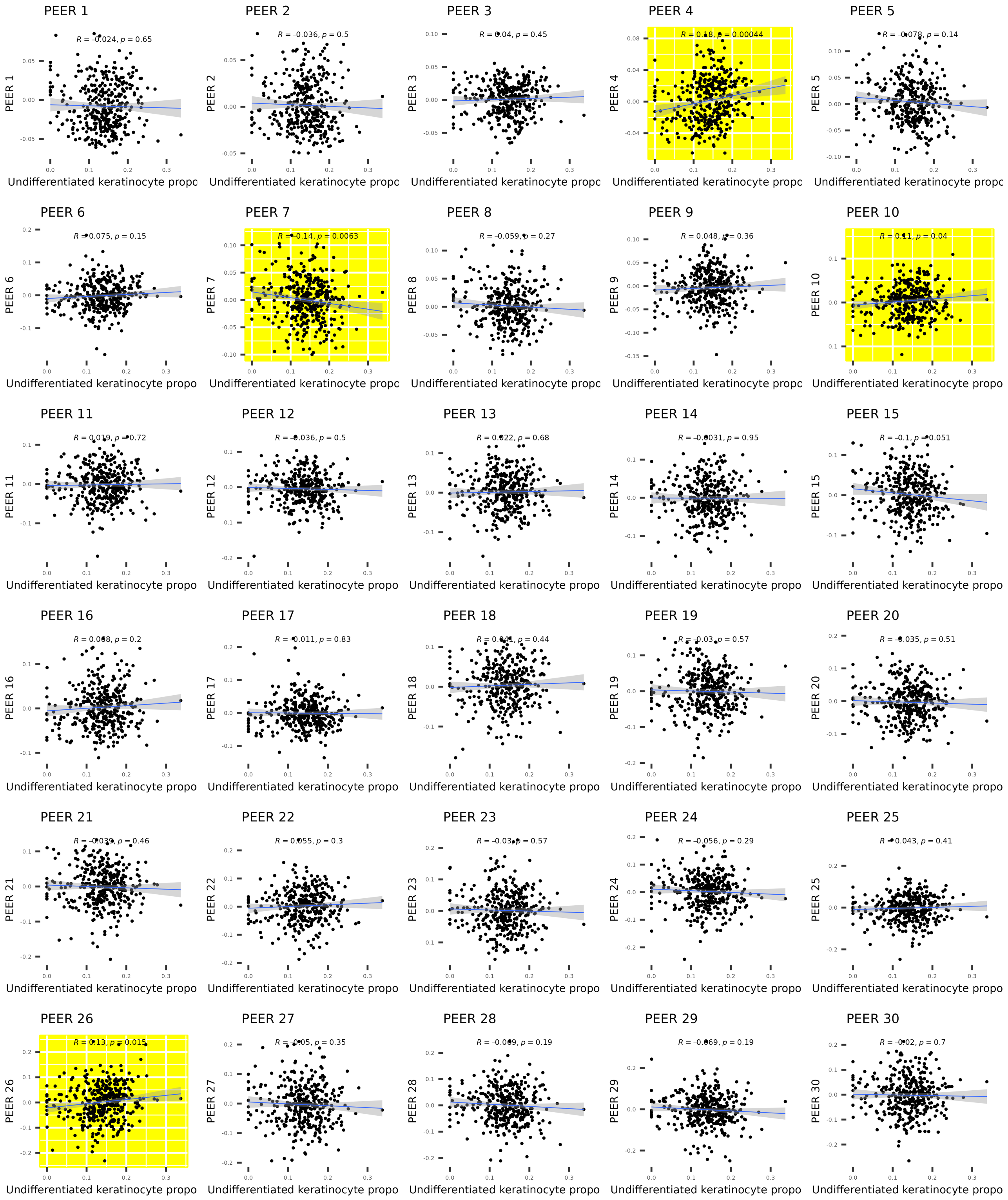

Figure S13. Scatter plots showing the correlation between the 30 PEER factors used as covariates in the skin eQTL analysis and Undifferentiated Keratinocyte Cell proportion as estimated from DNA methylation data. A yellow background indicates a nominally significant correlation (Pearson P<0.05).

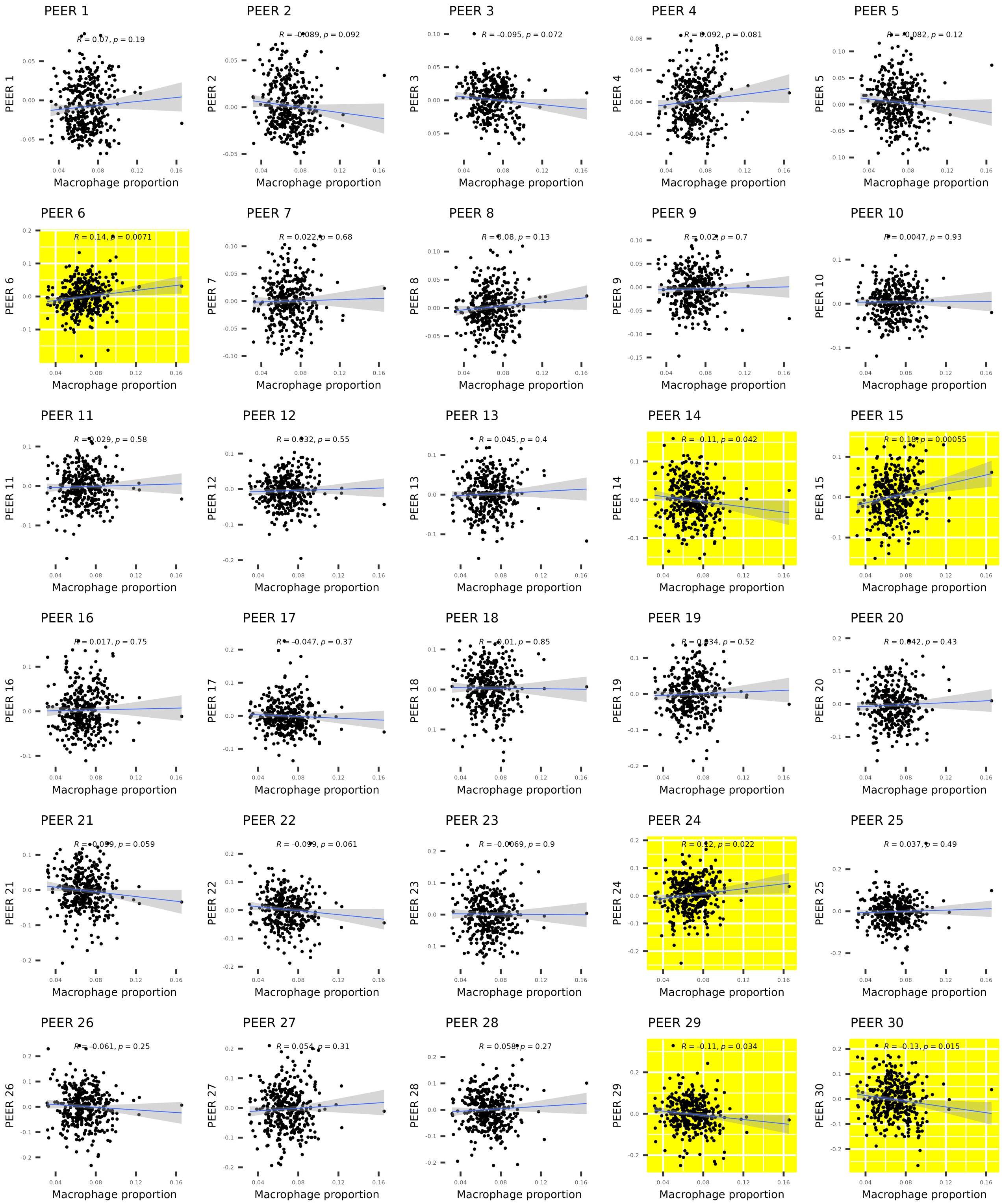

Figure S14. Scatter plots showing the correlation between the 30 PEER factors used as covariates in the skin eQTL analysis and Macrophage Cell proportion as estimated from DNA methylation data. A yellow background indicates a nominally significant correlation (Pearson P<0.05).

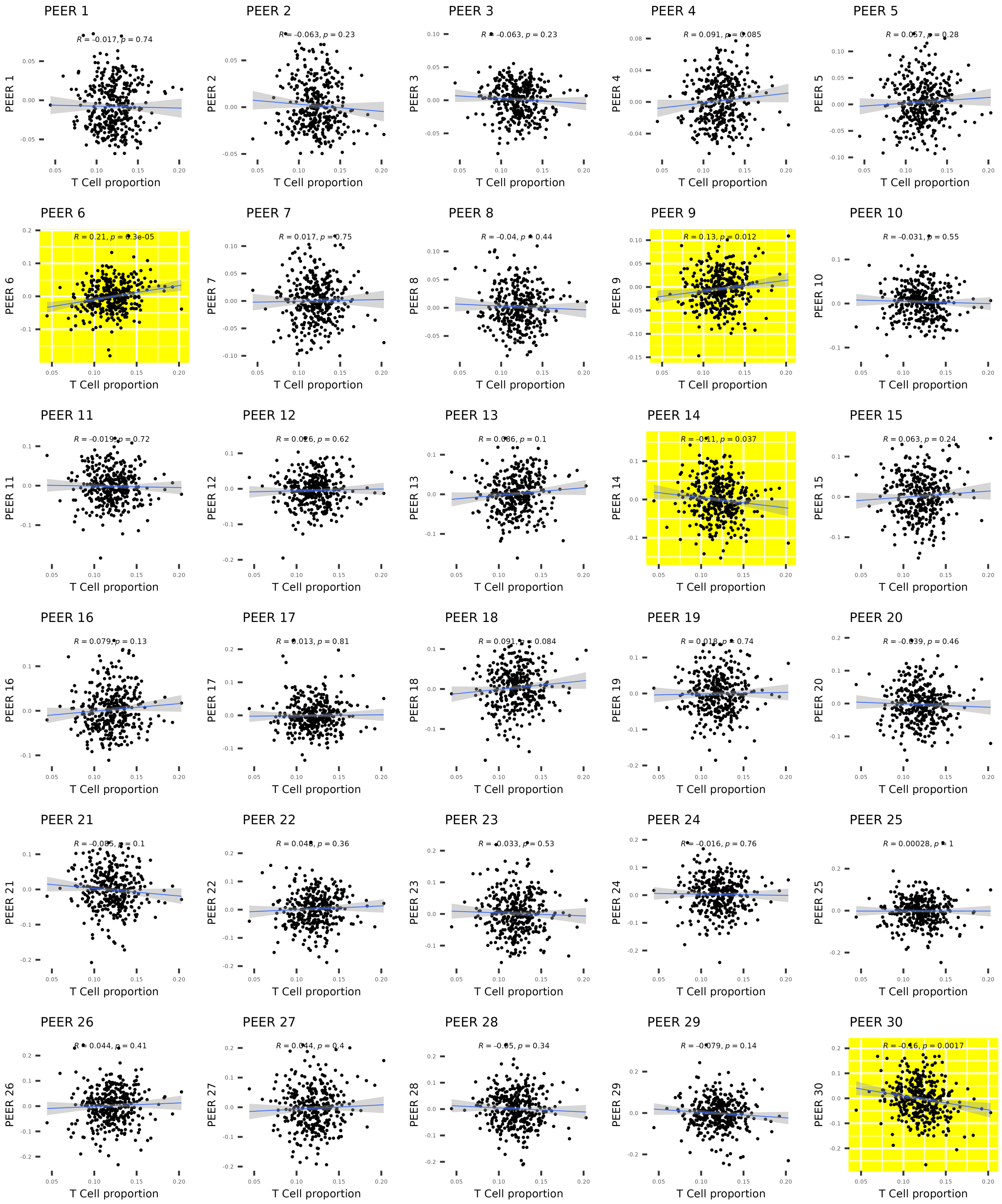

Figure S15. Scatter plots showing the correlation between the 30 PEER factors used as covariates in the skin eQTL analysis and T-Cell proportion as estimated from DNA methylation data. A yellow background indicates a nominally significant correlation (Pearson P<0.05).

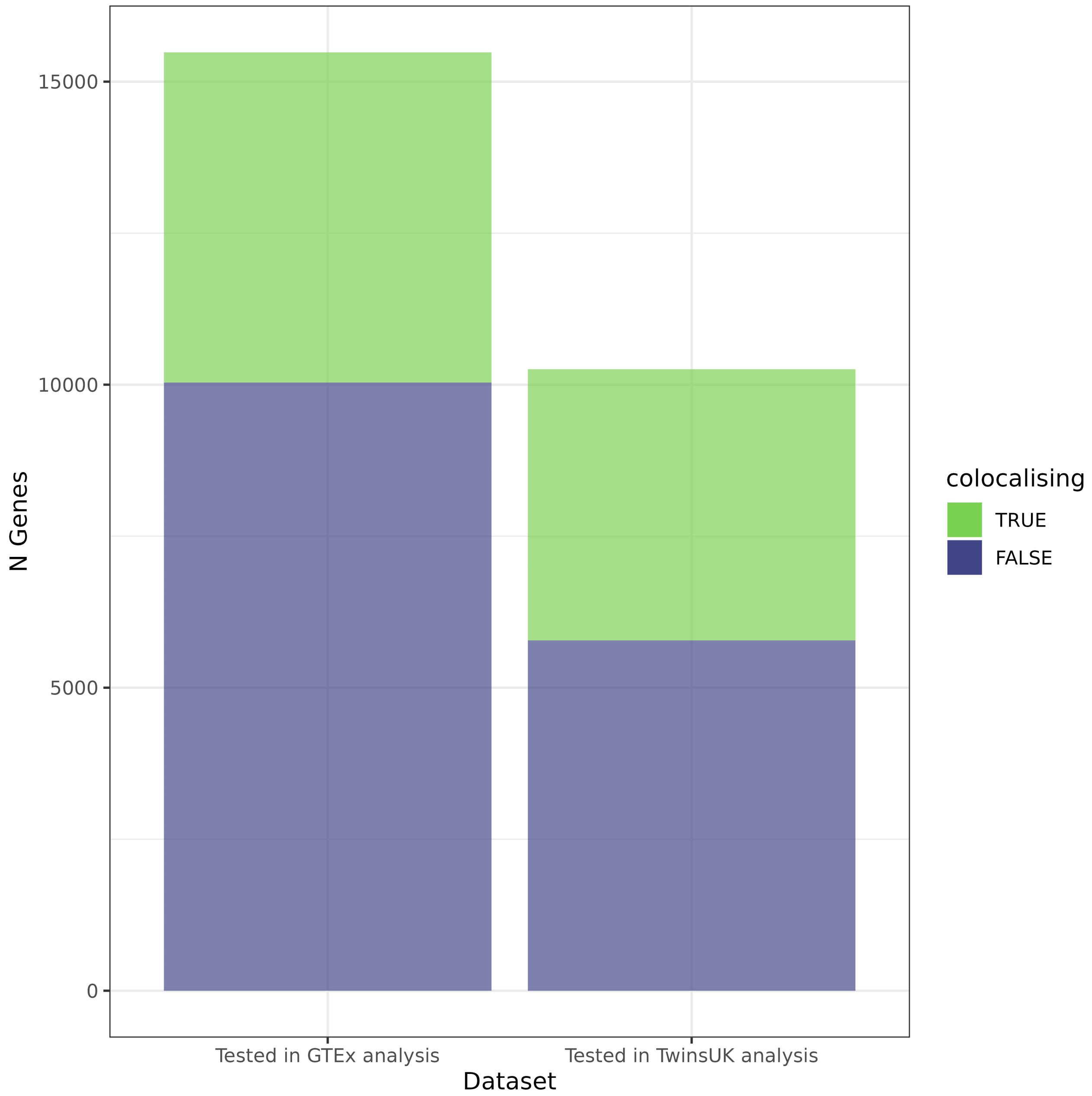

Figure S16. Proportion of eGenes colocalised in both the original meQTL-eQTL colocalisation analysis using TwinsUK data, and the validation analysis using the suprapubic skin GTEx eQTL data with TwinsUK meQTL data.

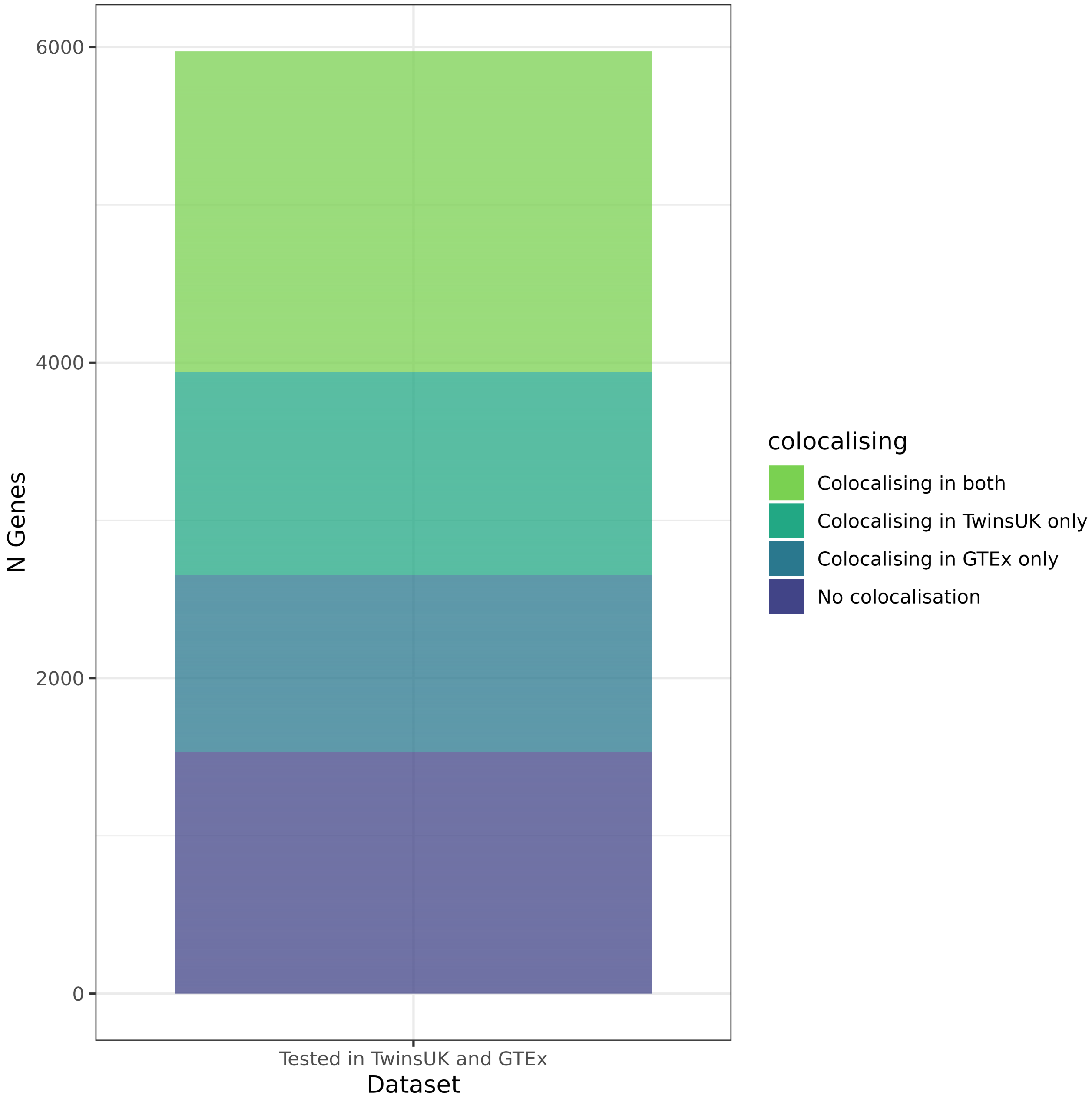

Figure S17. Proportion of eGenes tested in both the original meQTL-eQTL colocalisation analysis using TwinsUK data, and the validation analysis using the suprapubic skin GTEx eQTL data with TwinsUK meQTL data, which colocalise in each analysis.

Supplemental References

[1] Vandiver, A. R., Irizarry, R. A., Hansen, K. D., Garza, L. A., Runarsson, A., Li, X., Chien, A. L., Wang, T. S., Leung, S. G., Kang, S., et al. (2015). Age and Sun Exposure-Related Widespread Genomic Blocks of Hypomethylation in Nonmalignant Skin. Genome Biology. 16, 80.10.1186/s13059-015-0644-y.

[2] Min, J. L., Hemani, G., Hannon, E., Dekkers, K. F., Castillo-Fernandez, J., Luijk, R., Carnero-Montoro, E., Lawson, D. J., Burrows, K., Suderman, M., et al. (2021). Genomic and Phenotypic Insights from an Atlas of Genetic Effects on DNA Methylation. Nature Genetics. 53, 1311–1321.750.10.1038/s41588-021-00923-x.

[3] Guo, H., Fortune, M. D., Burren, O. S., Schofield, E., Todd, J. A., and Wallace, C. (2015). Integration of Disease Association and eQTL Data Using a Bayesian Colocalisation Approach Highlights Six Candidate Causal Genes in Immune-Mediated Diseases. Human Molecular Genetics. 24, 3305–3313.10.1093/hmg/ddv077.
